# Reconstruction and analysis of the genetic and metabolic regulatory networks of the central metabolism of *Bacillus subtilis*

**DOI:** 10.1186/1752-0509-2-20

**Published:** 2008-02-26

**Authors:** Anne Goelzer, Fadia Bekkal Brikci, Isabelle Martin-Verstraete, Philippe Noirot, Philippe Bessières, Stéphane Aymerich, Vincent Fromion

**Affiliations:** 1 Unité Mathématique, Informatique et Génomes, Institut National Recherche Agronomique, UR1077, F-78350 Jouy-en-Josas, France; 2 Unité de Génétique des Génomes Bactériens, Institut Pasteur, URA CNRS 2171, 28, rue du Docteur Roux, 75724 Paris Cedex 15, France; 3 Unité de Formation et de Recherche de Biochimie, Université Paris 7-Denis Diderot, 2 place Jussieu, 75251 Paris, France; 4 Unité Génétique Microbienne, Institut National Recherche Agronomique, F-78350 Jouy-en-Josas, France; 5 Microbiologie et Génétique Moléculaire, Institut National Recherche Agronomique Paris-Grignon, CNRS (UMR2585) INRA (UMR1238), F-78850 Thiverval-Grignon, France

## Abstract

**Background:**

Few genome-scale models of organisms focus on the regulatory networks and none of them integrates all known levels of regulation. In particular, the regulations involving metabolite pools are often neglected. However, metabolite pools link the metabolic to the genetic network through genetic regulations, including those involving effectors of transcription factors or riboswitches. Consequently, they play pivotal roles in the global organization of the genetic and metabolic regulatory networks.

**Results:**

We report the manually curated reconstruction of the genetic and metabolic regulatory networks of the central metabolism of *Bacillus subtilis *(transcriptional, translational and post-translational regulations and modulation of enzymatic activities). We provide a systematic graphic representation of regulations of each metabolic pathway based on the central role of metabolites in regulation. We show that the complex regulatory network of *B. subtilis *can be decomposed as sets of locally regulated modules, which are coordinated by global regulators.

**Conclusion:**

This work reveals the strong involvement of metabolite pools in the general regulation of the metabolic network. Breaking the metabolic network down into modules based on the control of metabolite pools reveals the functional organization of the genetic and metabolic regulatory networks of *B. subtilis*.

## Background

Recently, the reconstruction and analysis of genome-scale genetic and metabolic regulatory networks has become an area of active research. It requires the integration of existing knowledge as a first step towards systems biology, which we define as the study of the interactions between the components of biological systems to understand how these interactions give rise to the function and behavior of the system. Genetic and metabolic networks have been constructed for *Helicobacter pylori *[1], *Haemophilus influenzae *[2], *Lactococcus lactis *[3], *Escherichia coli *[4], and *Homo sapiens *[5], by incorporating the description of chemical reactions into a stoichiometric matrix. Transcriptional regulation by regulatory proteins has been included in the last update of the model of *Saccharomyces cerevisiae *[6,7]. However, most of these models do not explicitly include regulation mediated by metabolites, with the exception of the most recent version of the EcoCyc database [8]. Metabolites can modulate the activity of transcription factors and enzymes and are therefore key regulators.

Reconstruction of the metabolic network and its associated regulatory network contributes to resolving various problems such as unravelling how the bacterium coordinates its genetic and metabolic networks to adapt to environmental changes [9,10] and elucidating the global organization of the regulatory network [11,12]. Such work will also help develop tools and concepts to handle and to analyze the inherent complexity of biological functions. As this represents one of the central issues of the systems biology approach, metabolic networks have been the focus of in depth studies [13,14], and various tools have been developed to unravel the organization of these highly complex networks [15,16].

Here, we report the reconstruction of the genetic and metabolic regulatory network for the Gram-positive bacterium *Bacillus subtilis*. *B. subtilis *has been studied for over 40 years and is one of the best-characterized bacteria, easily amenable to genetic and physiological studies. The publication of the *B. subtilis *genome sequence [17] and subsequent international programmes of systematic gene disruption, functional analysis and regulatory network studies [18], makes this microorganism a prime candidate to develop systems biology. Our model includes the biochemical reactions of the metabolic network and all the known levels of regulation involved in metabolic pathways: transcriptional, translational, post-translational and modulation of enzymatic activities. To obtain the most complete view of the interplay between the metabolic network and genetic regulation, the description of each regulatory mechanism includes known roles of metabolite concentrations, ions, and any other quantities related to the state of the metabolic network. The entire model (reactions, enzymes, genes and regulations) has been curated manually, using published data and expert knowledge.

Using this model, we were able to examine various aspects of the general organization of metabolic regulation. We find that metabolite pools are strongly involved in regulation of the central metabolism of *Bacillus subtilis*, in agreement with the findings from an analysis of the genetic regulatory network of *Escherichia coli *[19]. Moreover, by introducing the notion of local and global regulation, we reveal that the complex regulatory network can be broken down into sets of locally regulated modules, which are coordinated by global regulators. Local regulations ensure that the control of elementary pathways through genetic and/or enzymatic regulation depends upon the level of key metabolites. The regulation of tryptophan synthesis by tryptophan through the TRAP regulatory protein is an example of such a local regulation. In contrast, global regulations ensure the coordination between these elementary pathways in response to environmental changes. For example, CcpA inhibits several pathways during glycolytic growth conditions. The integration of these local/global levels and the use of the classification of sensing signals in [19] lead to recover the main physiological aspects of metabolism of *Bacillus subtilis*. Finally, the notion of local and global regulation allows the graphic representation of any metabolic pathway and its various regulatory mechanisms.

## Results

### Model construction

Our aim was to study the general organization of the metabolic functions of *B. subtilis*, to identify the components involved in their regulations and to unravel how these regulations coordinate metabolism (involving various degradative and biosynthesis pathways of anabolism and catabolism). Pathways involved in the metabolic functions can be classified as "minor" or "major" according to the flux through the pathway. Typically, the "major" metabolic pathways are those involved in the production of energy, redox power, and metabolic precursors of proteins, RNA, DNA, membrane and cell wall. The "minor" metabolic pathways include the *de novo *synthesis of cofactors and secondary metabolites (including vitamins, coenzymes, and antibiotics). Most minor metabolic pathways are linear and poorly connected with other pathways, whereas major metabolic pathways are highly interconnected with other pathways, implying coordinated regulatory mechanisms. We focused on the major metabolic pathways of *B. subtilis*, and in particular: central carbon metabolism; aerobic and anaerobic respiration; fermentation; and amino acids, nucleotides and fatty-acids metabolism. We have not included the synthesis of precursors of cell wall components, such as peptidoglycan. The regulations of these pathways are poorly characterized, and not coupled with most of the major metabolic pathways.

The model was built from the reconstruction of these main metabolic pathways (see Methods). It corresponds to a so-called knowledge-based model describing the chemical reactions, known transcriptional, translational and post-translational regulations and controls of enzymatic activities. In contrast to classic representations of genetic regulatory networks that integrate only regulation by transcription factors [7], we have also incorporated transcriptional regulations by premature termination of transcription. The translational and post-translational regulations correspond to regulation (*i*) by sequestration of the Ribosome Binding Sequence (RBS); (*ii*) by small non-coding RNA; (*iii*) by sequestration of proteins and (*iv*) by proteolysis. We also systematically included (*i*) a description of enzymatic complexes, which are represented as a sum of genes, (*ii*) genetic organization in operons, and (*iii*) the number of promoters for each gene and the detailed conditions of transcription from each promoter (see Methods). The final model includes 534 genes, 563 reactions, 65 transcription factors (TF), 9 sigma factors and 21 other mechanisms of genetic regulation at the transcriptional or translational level (see Table 1). The model also includes 95 different genetic regulations involving either transcription factors (and sigma factors) or other mechanisms. This model provides a basis for future developments of dynamic models.

**Table 1:** Integration of information

Genes	534	organization in 186 operons
Metabolites	456	
Reactions	563	metabolic pathways, complexes, enzymatic regulation
Enzymatic regulations	79	including cofactors, ions and regulations by metabolites
Genetic regulations	65 TFs 9 Sigma factors 21 others*	with their metabolite effectors, distinction between activation and modulation
Functions	7	- central carbon pathway, overflow - fermentation, oxidative phosphorylation, - carbohydrate transport and degradation, - amino acid synthesis and degradation - nucleotide synthesis and salvage pathways - fatty-acid synthesis and degradation

*correspond to RtpA, S-box, L-box, A-box, G-box, Plead, SR_1 _and 14 T-boxes.

An essential aspect of this work is the manual curation of the information. We integrated the enzymatic reactions and all the regulation mechanisms which have been experimentally validated. The key literature references for each reaction and each regulation described are included in the model. Nevertheless, in a few instances, concerning fermentation and degradation of branched-chain amino acids (BCAA), the existence of a functional pathway is based on indirect evidence rather than on a detailed study; these pathways are annotated with a comment indicating that the level of knowledge is low. The complete set of data (see Additional file 1) and a review of the available information on *B. subtilis *(see Additional file 2 and 3) are presented in Additional files.

#### Comparison of our model with published phenotypes

Because our model integrates information that has been confirmed by experiments with the exception of fermentation and degradation of branched-chain amino acids, its validation and its biological relevance are intrinsic. However, it is interesting to assess whether our model recovers known phenotypes of *B. subtilis *mutants [18,20,21]. In particular, the effects of genetic regulations on prediction of the phenotypes of gene deletion can be evaluated. Using the methodology described in Methods, we have predicted whether a gene deletion is lethal or viable in two different growth conditions: a poor and a rich medium corresponding to a M9 glucose minimal medium and a LB medium, respectively (see Additional file 1 and Additional file 4 for their exact composition). Compared to the results obtained in [20], the introduction of genetic regulations in this analysis allowed the prediction of phenotypes associated to the deletion of genes encoding transcription factors (requirement of *gltC*, *cysL, rtpA *in minimal medium) and the recovery of so-called complex phenotypes resulting from the dysfunction of transcriptional regulation. One such example is the absence of growth of an *hprT *mutant on LB medium [18]. The *hprT *gene encodes the hypoxanthine-guanine phosphoribosyltransferase, which converts hypoxanthine and guanine in IMP and GMP, respectively (see Additional file 5, Figure S47), and avoids *de novo *synthesis of purines. Hypoxanthine and guanine are indeed the effectors of the G-box riboswitch [22] which inhibits the transcription of genes involved in *de novo *purine synthesis. In LB medium, the *hprT *mutant may accumulate hypoxanthine and guanine, which in turn may prevent the transcription of the *pur *operon via the G-box riboswitch. The absence of GMP synthesis would then prevent the growth of the *hprT *mutant even in LB medium. Of course, this prediction remains to be tested experimentally.

### Focus on the isoenzymes

Isoenzymes may be required in different physiological conditions or in different metabolic pathways, and their synthesis may be differentially regulated. To address this issue, we summarized in Additional file 6 all characterized isoenzymes in *B. subtilis *and their regulations. The regulation of synthesis and/or enzymatic activities is known for 56 of the 80 isoenzymes characterized. Most of these 56 isoenzymes fall into two categories: (*i*) they are active in different physiological conditions (vegetative growth, sporulation, etc.); (*ii*) they are involved in distinct metabolic pathways with the existence of differential enzymatic and/or transcriptional regulations. Two detailed examples that typify these two different categories are illustrated.

The asparagine synthases encoded by the *asnO*, *asnB *and *asnH *genes are an example of the first category of isoenzymes. In a rich sporulation medium, *asnB *is expressed only during exponential growth. *asnH *transcription increases during the transition between exponential growth and stationary phase while *asnO *is expressed only later in sporulation. In a minimal medium, both *asnB *and *asnH *are expressed constitutively during exponential growth and in stationary phase, but the expression of *asnO *is not detected in either phase [23]. The mechanism of regulation of *asnB *expression is still unknown whereas *asnH *belongs to the CodY regulon [24]. The release of CodY repression during the transition between exponential growth and stationary phase is responsible for the derepression of *asnH *transcription. The *asnO *gene is transcribed from a *σ*^E^-specific promoter, which explains its induction only during sporulation.

The second category can be illustrated with the carbamoyl-phosphate-transferase. This two-subunit enzyme produces carbamoyl-P from CO_2_, glutamine and ATP. The carbamoyl-phosphate-transferase is involved in two pathways: arginine biosynthesis and pyrimidine synthesis. In *B. subtilis*, this enzyme is encoded by two sets of genes: *carA *and *carB*, or *pyrAA *and *pyrAB*. *carA *and *carB *belong to an operon with other genes involved in arginine synthesis. The expression of this operon is repressed by AhrC using arginine as corepressor [25]. *pyrAA *and *pyrAB *belong to the pyrimidine operon, which is regulated by PyrR in response to the availability of metabolites of the pathway (UMP, UTP and PRPP) [26]. Each enzyme belongs to a different pathway, and is synthesized under particular physiological conditions thereby avoiding functional redundancy. By contrast, only the *carAB *operon is present in *E. coli*. This operon is transcribed from two promoters, one controlled by arginine through the transcription factor ArgR, and the second controlled by pyrimidine. In addition, the enzyme is activated by ornithine and IMP, and inhibited by UMP [27]. These two organisms have developed different regulatory solutions to achieve the same goal: the production of carbamoyl-P for the syntheses of arginine and pyrimidine.

Few isoenzymes appear to be controlled by the same regulation (see Additional file 6). The pairs of amino-acyl tRNA synthetases (*tyrS/tyrZ, thrS/thrZ*) both seem to be transcribed under the control of T-boxes specific for tyrosine or threonine, but are active in different physiological conditions [28,29]. Thus, they could belong to the first category. Other isoenzymes are known to be regulated in the same way and synthesized in the same physiological conditions: (*i*) the OAS thiol-lyases (*cysK, mccA*) [30], (*ii*) the cysteine desulfydrases (*cysK, mccB*) [31], (*iii*) enzymes involved in fatty acid metabolism (the *α*-keto-acyl synthase, acyl-CoA dehydrogenase, and 3-hydroxyacyl-CoA dehydrogenase/enoyl-CoA hydratase complex) [32-34,9]. These isoenzymes can catalyze different chemical reactions *in vitro *and present different and complementary activities with respect to their substrates.

### Structure of the genetic regulatory network: key role of metabolic pools in general control

Our model allows a detailed analysis of the interplay between metabolic pathways and genetic regulation through a large set of metabolic pools (and others effectors). Among the 79 genetic regulators whose effector is known, 70% (59) have a metabolite as effector (see Table 2). Furthermore, eight additional regulators respond to an ion or to a component of the PTS, and are thus strongly coupled to the metabolic network as they are linked to the availability of particular glycolic sugars through a component of the PTS. The remaining 13 genetic regulators have no effectors: 4 genetic regulators, AbrB, RtpA, YtlI and SR_1_, which are all under the control of other(s) genetic regulator(s) (resp. Spo0A, T-box and Plead, CymR and Spx, CcpN and CcpA), and nine sigma factors. The effectors for 15 genetic regulators are still unknown. However, indirect evidences from the literature suggest that at least eight of these may respond to a metabolite effector, because they are part of two-component systems (YycF, ResD), are involved in carbohydrate catabolism (YtrA, FruR, RbsR) or regulate the synthesis of specific enzymes in response to the nutrient availability (CysL, CcpN, GlnR). The remaining genetic regulators are ArfM, Spo0A, SpoVT, SpoIIID, GerE, CcpB and Spx.

**Table 2:** Classification of genetic regulators according the nature of their effectors

Genetic regulators	TFs	Sigma factors	Others	Total
Metabolic effector	40	0	19	59
Effector related to metabolism (ion, PTS component)	8	0	0	8
No effector	2	9	2	13
Putative metabolic effector	8	0	0	8
Unknown effector	7	0	0	7

Total	65	9	21	95

#### Essential role of metabolites in genetic regulations

The interplay between the metabolic pathways and the genetic regulations is very strong: at least 13% of 456 metabolites are involved in genetic regulation and 53% of the genes involved in metabolic pathways are directly regulated by a genetic regulator under the control of a metabolite. Metabolites are also strongly involved in the control of enzymatic activities. Thus, metabolites are key actors in the general regulation of metabolic pathways through these two levels of regulation (genetic and enzymatic). Each metabolic pathway of *B. subtilis *for which the regulation is known, is under the direct control of a metabolite through a genetic or an enzymatic regulation (see Additional file 1): at least one gene (resp. one enzyme) of each pathway is controlled by a metabolite through a genetic (resp. enzymatic) regulation.

#### Hierarchical organization of the genetic regulatory network

We analyzed the hierarchical organization of the genetic regulatory network [35,36] by examining the cascade of regulations induced by each genetic regulator (GR). A GR of level 1 does not regulate other GRs. A GR will be on level *n *if the highest level of the GRs that it regulates is (*n *- 1). In *E. coli*, only 23 GRs are on level 3 or higher, and only two are level 5 (belong to the highest level) [36].

**Figure 1:**
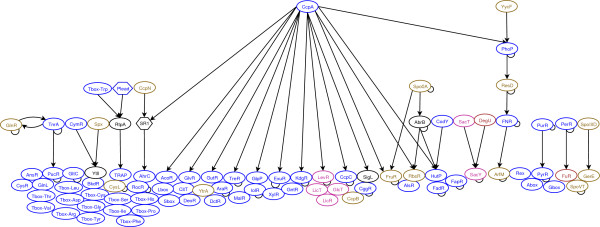
Hierarchical architecture of the genetic regulatory network. Each ellipse refers to a mechanism of transcriptional regulation, including: (*i*) transcription factors; (*ii*) early termination of transcription (T-box, L-box, G-box, A-box, S-box, TRAP); (*iii*) proteins involved in transcriptional regulation (RtpA). Each hexagon refers to a mechanism of translational regulation, including: (*i*) small RNA (SR_1_); (*ii*) peptide leaders (Plead). The arrow indicates the causality of the regulation. A regulator with a curved black arc indicates that it is subject to auto-regulation. Blue, magenta and dark red regulators have a metabolite, a PTS component, and an ion, respectively, as effector. The effector of brown regulators is unknown. Black regulators have no effector.

The hierarchical organization of the genetic regulatory network of *B. subtilis *is presented in Figure 1. This genetic regulatory network includes not only transcription regulators, but also mechanisms of control by premature termination of transcription (T-box, G-box, A-box, L-box, S-box) and translational regulations by small regulatory RNAs (SR_1_) and by peptide leaders (Plead). Sigma factors have not been included in Figure 1. Five levels of regulation were found in this analysis of the major metabolic pathways of *B. subtilis*.

CcpA and YycF are the regulators of the highest level in the genetic regulatory network (level 5). YycF is indirectly involved in the regulation of metabolic pathways (respiration and fermentation) via a cascade of regulations involving PhoP, ResD, FNR and ArfM. CcpA, which manages carbon uptake and catabolism, is connected to the PhoP, ResD, Fnr and ArfM cascade. 17% of genetic regulators are under the direct control of CcpA. Other known pleiotropic regulators, like DegU and Spo0A, are at level 2 or 3. Their observed level will presumably increase as genetic networks involved in other biological processes, such as sporulation or competence, are added to the model. However, DegU and Spo0A clearly modulate few different enzymes and they do not seem to make a large contribution to the direct regulation of metabolic pathways. This suggests a partial decoupling between the control of metabolic pathways and other biological processes. Metabolic pathways are mainly under the global control of CcpA, CodY and TnrA. Other pleiotropic transcription factors (DegU, Spo0A, AbrB) only modulate the expression of very few specific enzymes which seem to be mainly involved in other biological processes. For example, CitB is required during sporulation [37] and its synthesis is controlled by CodY and AbrB [38].

#### Identification of functional elementary modules through local and global regulations

All the genetic regulators of level 1 and some of level 2 regulate a specific pathway and their potential metabolite effectors belong to the controlled pathway (see Additional file 1). This control of a pathway as a function of the pool of effector metabolite was termed "local regulation". In turn, the set of enzymes associated with a local regulation and the local regulators define a functional "elementary module" (see details in the Discussion). For example, the enzymes TrpE, TrpG, TrpD, TrpF TrpC, TrpA, TrpB involved in the tryptophan synthesis together with the regulators TRAP, RtpA, the tryptophan-specific T-box and the tryptophan-rich peptide leader Plead define an elementary module (see Additional file 5, Figure S26). Indeed the enzymatic complex TrpE+TrpG is regulated by TRAP at the genetic level, and by tryptophan at the enzymatic level. This elementary module definition is based on the mathematical developments given in Additional file 7, which describes the key control properties associated to local regulations (according to our definition). Elementary modules have been manually identified during the curated reconstruction of the genetic and metabolic network of *B. subtilis *by a strict application of the local regulation definition. Global regulators of a metabolic pathway are defined as any non-local regulators involved in the control of this pathway. The set of global regulators in the metabolic network is composed by all the genetic regulators that have been identified at least once as global regulators of a metabolic pathway. They mainly correspond to level 2 to 5 regulators. Additional file 8 presents the local/global classification of all genetic regulators of the reconstructed regulatory network. We have previously identified CcpA, TnrA and CodY as the three most important regulators involved in the control of metabolic pathways, and the analysis of their local or global roles in regulation is summarized.

**CcpA **is the main regulator responsible for catabolite repression in *B. subtilis*, thus managing the carbon resources [39]. Fructose-1,6-biphosphate (FBP) is considered to be the main metabolite effector of CcpA. Although CcpA binds to DNA as a complex with HPr-Ser46-P, FBP is required both for the phosphorylation of HPr by the HPr kinase and for the formation of the CcpA/HPr-Ser46-P complex [40,41]. CcpA directly regulates carbohydrates, fatty-acids and amino acids degradation (see Additional file 6, Table S3 for details). As FBP is not an intermediate metabolite in these pathways, CcpA is a global regulator for these pathways.

**TnrA **is the main regulator involved in nitrogen management [42]. In excess of glutamine, glutamine binds to the glutamine synthase GlnA. This feedback-inhibited glutamine synthase sequestrates TnrA [43], so glutamine can be considered as the metabolite effector of TnrA. TnrA acts on various metabolic pathways, including asparagine and purine degradation [44,45] leading to NH_3 _production, where glutamine is not directly involved. Therefore, TnrA belongs to the set of global regulators.

**CodY **acts on various metabolic pathways, including BCAA synthesis, and histidine and asparagine degradation leading to glutamate production. CodY, which is also involved in the control of competence, mobility and sporulation [24], is a global regulator. However, CodY can be considered as a local regulator for BCAA synthesis. Indeed, GTP and branched-chain amino-acids are the signaling molecules sensed by CodY [46], but as the GTP effect on CodY-dependent regulation of BCAA synthesis is weak [47], BCAA can be considered as the main metabolite effector for CodY in this pathway.

Among the other global regulators with less important roles in the general control of metabolic pathways (see Additional file 8 and previous paragraph), two subsets can be distinguished. The first subset is composed of global regulators (*i*) managing the metabolic function according to the depletion of a critical resource, for example phosphate availability through the two-component system PhoPR [48], and (*ii*) adapting the metabolic network to increase/decrease the production of some metabolites, for example the stimulation of the conversion of cysteine from methionine by Spx when methionine is the sole sulphur source [49]. Clearly, the two global regulators chosen as examples are under the control of specific signals, although that sensed by Spx remains un-characterized. The second subset corresponds to global regulators involved in the control of complex biological processes such as sporulation (SpoIIID, SpoVT, Spo0A) and the transition from exponential to stationary phase (AbrB and DegU).

#### Remark on the identification of functional elementary modules

In some cases, the identification of elementary modules is controversial, because (*i*) it does not correspond to a classical metabolic pathway (e.g. glycine synthesis); (*ii*) isoenzymes often belong to different elementary modules (e.g. asparagine and proline syntheses); (*iii*) local regulators in an elementary module can also correspond to global regulators for other modules (e.g. CodY). Three examples of these ambiguities – glycine, proline and branched-chain amino acid syntheses – are presented below. Glycine is synthesized in one step from serine by GlyA (see Additional file 5, Figure S13). The expression of *glyA *is repressed by PurR, the global regulator involved in purine synthesis [50]. Glycine is also required in purine synthesis (see Additional file 5, Figure S43). As a consequence, GlyA belongs to the purine synthesis module, and is not an autonomous module.

The main difficulty for module identification in proline synthesis (see Additional file 5, Figure S23) is the presence of isoenzymes (ProB/ProJ and ProG/ProH/ProI). There are two different modules: one (*proA*, *proB *and *proI*) is probably controlled by the proline specific T-boxes located upstream from *proI *and *proBA*, and is required in anabolism; the second (*proJ *and *proH*) is induced during osmotic stress. The regulation of the remaining isoenzyme ProG is unknown, so it is difficult to assign it to these or a third module.

The branched-chain amino acid synthesis (see Additional file 5, Figure S15) is controlled by CcpA, CodY, TnrA [51] and a leucine-specific T-box [52]. CcpA and TnrA are two global regulators while the T-box-dependent control is a local regulation. Despite its global regulatory role, CodY, as explained previously, is a local regulator for this pathway.

### A graphic representation of metabolic pathways and their associated regulations

**Figure 2:**
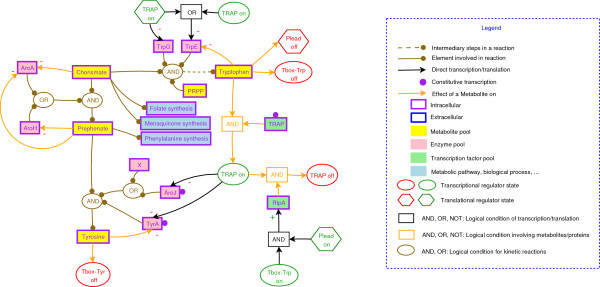
Graphic representation of the synthesis of tyrosine from chorismate. Yellow (resp. pink, green) boxes correspond to metabolites (resp. enzymes, transcription factors) pools. The black (resp. orange) arrows correspond to transcriptional/translational (resp. enzymatic) regulations. The effect of the regulation is indicated by the sign "-" in the case of a repression/inhibition, or by the sign "+" in the case of an induction/stimulation. A closed purple circle, associated with an enzyme, means that the gene(s) encoding the corresponding enzyme may be constitutively transcribed. An ellipse corresponds to a transcriptional regulator (transcription factor, T-box) that can be "on" (in green) or "off" (in red). The state "on" is defined as "able to bind to DNA". A hexagon represents a translational regulator that could also be "on" (in green) or "off" (in red). The translational regulator is defined as "on" when it is able to bind to mRNA.

We have developed a graphic representation of metabolic and genetic regulatory networks, which allows the visualization of different levels of regulation (transcriptional, translational, protein-protein interactions, enzymatic control). A graphic representation for each metabolic pathway is available in Additional file 5. The graph provides a view of metabolic pathways with the key regulatory players represented (Figure 2). The steps of a metabolic pathway catalyzed by enzymes whose regulations are unknown are not detailed. The graph also displays the metabolite pools, which are (*i*) precursors for the pathway, (*ii*) nodes of control, and (*iii*) involved in regulations through premature termination of transcription, transcriptional factors or enzymatic controls. The formalism used for the representation of pools of metabolites, enzymes and transcription factors is described in the legend of Figure 2. By convention, the level of the metabolite pools that are involved in regulations is high, and the level of the enzymes and transcription factors pools depends on their genetic regulations. The representation simplifies a complex genetic regulation (available in detail in Additional file 1) in a Boolean condition between the players (a metabolite effector, a sequestrated protein) involved in the regulation.

To illustrate exploitation of this representation, we used the biosynthesis of tyrosine from chorismate as an example because it combines all the types of control (Figure 2). The pathway is subject to diverse types of regulation: (*i*) enzymatic control, (*ii*) transcriptional and translational regulation involving the TRAP repressor, (*iii*) control by premature termination of transcription at the T-box, and (*iv*)protein-protein interactions with the sequestration of TRAP by RtpA.

Tyrosine is synthesized from chorismate in three steps, with intermediate production of prephenate. Chorismate and prephenate are also precursors of other metabolic pathways (folate and menaquinone synthesis for chorismate, and phenylalanine synthesis for prephenate). Prephenate is formed from chorismate by two chorismate mutases, encoded by the *aroA *and *aroH *genes [53]. AroA is a bi-functional enzyme, with chorismate mutase and 3-deoxy-D-arabino-heptolosonate-7-phosphate (DAHP) synthase activity [54]. The synthesis of prephenate from chorismate is summarized in Figure 2 as

prephenate = chorismate AND (AroA OR AroH).

Chorismate and one of AroA and AroH are necessary to produce prephenate. There is no information available concerning the transcription of *aroA *and *aroH*. However, AroA and AroH activities are inhibited by prephenate [53,54], and AroA activity is also inhibited by chorismate. The inhibition of AroA by prephenate and chorismate is represented by two orange arrows that link the prephenate and chorismate metabolite to the AroA enzyme, with a "-" sign. The AroH inhibition by prephenate is similarly represented.

Tyrosine is synthesized from prephenate in two steps by the sequential action of the prephenate dehydrogenase, TyrA, and aminotransferases, AroJ and an unknown protein (defined as *X *on Figure 2). TyrA activity is feedback-inhibited by tyrosine [55], and enzymatic control represented in Figure 2 by the orange arrow that links tyrosine to TyrA.

*aroJ *and *tyrA *belong to the tryptophan operon (*trp *operon), transcribed from a *σ*^A ^promoter. The transcription of the entire operon is modulated by termination/antitermination of transcription involving the TRAP protein. TRAP binds to the nascent mRNA and prevents elongation of transcription. The translation of two genes of the *trp *operon (*trpE *and *trpD*) is further modulated by TRAP (see below). *tyrA *and *aroJ *are also transcribed from an internal constitutive *σ*^A^-dependent promoter [56]. In our model, the control of *tyrA *transcription by the two promoters is described thus

$$\underset{TRAP\;regulated\;promoter}{\underset{︸}{\left\{[\sigma^{\text{A}}]\}ANDNOT(\text{TRAP}\right)}}OR\underset{Constitutive\;promoter}{\underset{︸}{[\sigma^{\text{A}}].}}$$

The transcriptional regulation of a gene is modelled by a black arrow, which links the regulator to the enzyme (or more generally the protein) that the gene encodes. The direction of the regulation is indicated by the sign "+" (induction) or "-" (repression). The transcriptional regulator, represented by an ellipse in Figure 2, may act on the transcription of a gene by any of the known mechanisms: classical control of the initiation of transcription by regulators and termination/antitermination mechanisms (T-boxes, S-boxes, G-boxes, etc). By default, the absence of a black arrow or a purple circle for an enzyme means that no information is available about the transcription of the corresponding gene. Several mechanisms can control an enzyme pool: AroJ is both linked to an arrow (control by TRAP at the first promoter) and to a purple circle (constitutive transcription of the internal promoter) in Figure 2.

In the presence of tryptophan (TRP), TRAP is active and binds to the nascent *trp*-mRNA (state "on"). The anti-TRAP protein (RtpA) can sequester active TRAP associated with TRP by protein-protein interactions. The TRAP-RtpA complex is unable to bind to mRNA, leading to the transcription of the TRAP regulon. The state "on" of TRAP, which is defined as the conditions that enable the binding on mRNA, is described by

TRAP_on _= TRAP AND TRP AND NOT(RtpA).

TRAP also modulates the translation of the *trpE *and *trpD *genes by binding to the complete *trp*-mRNA. The *trpE *ribosome-binding site (RBS) is then sequestrated in a RNA secondary structure preventing ribosome binding and *trpE *translation. The coding sequences of *trpE *and *trpD *overlap by 29 nucleotides thereby coupling the translation of these two genes. The translation of *trpD *is also affected by the binding of TRAP to the *trpE *RBS [57]. In our graphic representation, a mechanism of translational regulation is modelled by a hexagon, which is bound (state "on") or not (state "off") to the mRNA (Figure 2). TRAP is represented both by an ellipse for transcriptional control and by a hexagon for translational control. So, the repression of TrpE synthesis results in a combination of transcriptional- and translational-dependent modulations:

$$\underset{\begin{array}{c} transcriptional\\ TRAP\;regulation \end{array}}{\underset{︸}{{\text{TRAP}}_{\text{on}}}}\text{OR}\underset{\begin{array}{c} translational\\ TRAP\;regulation \end{array}}{\underset{︸}{{\text{TRAP}}_{\text{on}.}}}$$

Because the transcription of the *mtrB *gene encoding TRAP is constitutive, the free TRAP concentration is correlated only with the amount of anti-TRAP protein RtpA. *rtpA *transcription is regulated by a tryptophan-specific T-box, and its translation is modulated by the translation of a peptide leader RtpLP, located upstream from the *rtpA *gene. Three codons for tryptophan are present in the CDS of RtpLP. Complete translation of RtpLP prevents the translation of RtpA (for a review on TRAP regulation see [57]). The regulation of the translation of *rtpA-*mRNA by the peptide leader (Plead) is represented in Figure 2 by a hexagon, because it corresponds to a translational regulation. An "off" state for translational regulation is defined as the conditions that prevent the translation of the mRNA. In our example, the "off" state of the peptide leader corresponds to the presence of tryptophan:

Plead_off _= TRP

Finally, the activity of the anthranilate synthase (TrpE+TrpG), which catalyzes the first step of synthesis of tryptophan from chorismate, is also inhibited by tryptophan [55].

In conclusion, the synthesis of tryptophan, which proceeds in several steps from chorismate, is simplified and reduced to one step in the graph of the tyrosine synthesis pathway (Figure 2). The major points of regulation are indicated: (*i*) inhibition of the anthranilate synthase by tryptophan; (*ii*) TRAP-dependent modulation of the transcription of all the genes involved in the synthesis of tryptophan; (*iii*) TRAP-dependent modulation of the translation of *trpE *and *trpD*; (*iv*) TRAP sequestration by RtpA; and (*v*) conditions of transcription and translation of RtpA.

The graphic representation of tyrosine synthesis displays the main players involved in the regulation of this pathway (TRAP, prephenate, chorismate, tyrosine and tryptophan) and whether their action is enzymatic, transcriptional or translational.

## Discussion

### General organization of the regulatory network

**Figure 3:**
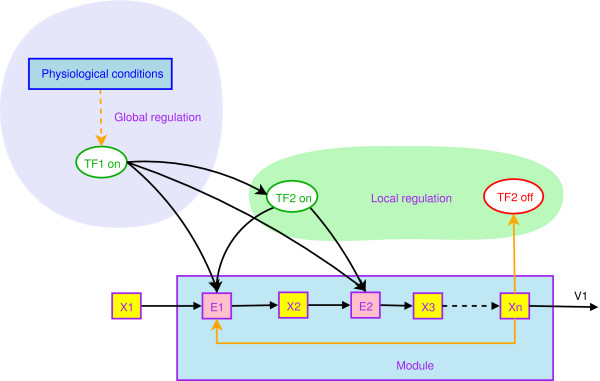
Global and local regulation of a metabolic pathway. Yellow (resp. pink) boxes represent metabolite (resp. enzyme) pools. The transcription factors are represented by ellipses. Green and red indicate that the transcription factor is "on" and "off", respectively, where "on" means that the transcription factor is able to bind to DNA. The local transcription factor (TF_2_) is sensitive to an intermediate metabolite (X_n_), and modulates the synthesis of enzyme(s) involved in the pathway. The global regulator TF_1_, sensitive to another signal, can modulate (*i*) the synthesis of intermediate enzymes; (*ii*) the synthesis of the local transcription factor TF_2_, or (*iii*) both.

We investigated the general organization of the *B. subtilis *metabolic pathways and searched for possible rules of regulations. The organization emerges when all the levels of regulations involved in a particular metabolic pathway are taken together. Two levels of regulation appear clearly (see Figure 3): one **local **and one **global**. The local regulation corresponds to any type of regulation (transcriptional, translational, termination/antitermination, inhibition/activation of enzymatic activity) which involves a metabolite of the pathway controlled. This specific metabolite will be refered to as the "key metabolite". Local regulation guarantees the activation or the inhibition of a pathway as a function of the pool of key metabolite(s) (see proposition 2 in Additional file 7). The notion of local regulation allows identification of functional elementary modules in the metabolic network.

Global regulation of a specific metabolic pathway is defined as a complementary set of the local regulation. Remarkably, we notice that our global regulation coincides with global regulation defined by biologists [10,58], which strengthens the relevance of the defined regulation levels.

### From local and global regulation to the emergence of functional elementary modules

As pointed out by several authors [59,60], no consensus on the definition of a regulatory module exists. Here, we propose a definition for the metabolic network based on the *in vivo *behaviour of regulations through metabolites: a functional "**elementary module" **corresponds to (*i*) a set of enzymes, which are either regulated by a local regulation, or constitutive (without direct local regulations) and located in the pathway between the key metabolite and the first locally controlled enzyme and (*ii*) the associated local regulator(s). This definition combines both the genetic and the metabolic levels through the feedback of metabolites on enzymes and genes via enzymatic regulations and effectors of genetic regulators, while other definitions of module are based solely on the genetic level and clustering [59-61].

The identification of the functional elementary modules (see Results) allows analysis of the impact of the local regulators on modules.

#### Local regulation for the induction/repression of functional elementary modules

Two classes of local regulation can be observed in the metabolic network of *B. subtilis*. In the first class, the metabolite effector is the end-product of the pathway; this is the case for almost all amino-acids biosyntheses. The local regulator induces enzymes synthesis of the pathway when the concentration of its effector decreases. In the second and more frequent class, the metabolite effector is an intermediate metabolite of the pathway like, for example the PRPP, which induces purine biosynthesis by preventing the binding of the repressor PurR to DNA [62]. Regulation of this type is mostly found in degradative pathways only induced if the first substrate is available, in the central carbon pathways with CggR in glycolysis [63] and CcpC in the TCA cycle [64], and in fatty-acids biosynthesis with FapR [65]. The local regulator of this class induces enzymes synthesis when the concentration of its effector (the first or an intermediate metabolite) increases. In both cases, the local regulation causes an increase of the metabolic flux through the pathway upstream (for the first class) or downstream (for the second class) the metabolite effector (see proposition 2 and 4 Additional file 7). This allows a qualitative prediction of the modulation of the various quantities attached to the elementary module (abundance of mRNA, enzyme activities, and fluxes) in response to changes of the concentration of metabolite effectors. To summarize, the local regulations lead to the emergence of functional elementary modules which can adapt the pathway capability according to the physiological needs of the cell and to the availability of precursor metabolites.

#### Global regulators for the general coordination of functional elementary modules

The global regulators, defined as non local regulators, are listed in Additional file 8; this group contains the well-known "pleiotropic" regulators [66,10,58]. Two main effects of global regulators on a module can be found: (*i*) turn on/off a module, (*ii*) modify/modulate the link between the metabolite effector of the local regulator and the module induction. The nature of the regulation associated with global regulators is summarized in Additional file 8.

#### Turn on/off effect

The effect of a genetic regulation is often simplified to an on/off view of the pathway induction in the modelling process [67]. In these conditions, the laws of pathway induction can be described by Boolean logic conditions, which are easy to manipulate and to simulate [68]. Although this description is often approximate, some global regulators clearly belong to this category. Such global regulation behaviour is often associated with a direct effect of the global regulator on local regulation (see Figure 3). In all these cases, the role of this kind of global regulator is to shape or reshape the metabolic network by allowing the induction of new elementary modules. However, the induction of a particular module usually remains under the control of its own local regulator. For example, the expression of the *iol *operon (involved in myo-inositol catabolism) is completely abolished by CcpA in the presence of glucose plus inositol, while in the presence of inositol alone, the local regulator IolR is required for its induction [69].

#### Modify/modulate local regulation rule

In this second category, a global regulator acts on an elementary module in a concerted fashion with the local regulator. The global regulator can modulate the local regulation by (*i*) binding directly to DNA, (*ii*) disturbing the local regulator binding. The aim of the global regulation is to modify the effect of the variation of the metabolite effector concentration of the local regulator on the module synthesis. The modulation of arginine degradation by CcpA through the control of *σ*^L ^production belongs to the second category, illustrating that these categories are not exclusive. Arginine can be used as sole nitrogen source when glucose is used as the carbon source [70]. The genes involved in the arginine degradation are transcribed from a *σ*^L^-dependent promoter, and induced by RocR in the presence of ornithine or citrulline [71], and AhrC in the presence of arginine [25]. CcpA, which inhibits the transcription of *sigL*, indirectly represses arginine degradation [72]. A *B. subtilis sigL *mutant cannot use arginine or ornithine as sole nitrogen source [73], so the *σ*^L ^concentration must be low but sufficient to allow arginine degradation in the presence of glucose.

The nature (turn on/off or modulation) of a global regulation in a metabolic pathway allows to build hypotheses on its physiological role, and to make predictions on the involvement of the pathway in the general organization of the metabolic function. For arginine degradation, the presence of CcpA-dependent regulation suggests that arginine or ornithine can also be used as sole carbon source. This study can be extended to all the metabolic pathways. In particular we can analyze the uptake and degradation of the different amino acids and predict if they can be used as carbon or nitrogen sources and also identify those that might have a "special" status with respect to the metabolic function (see Additional file 4).

### From functional elementary modules to signaling pathways

Since the genetic and metabolic regulatory network is mainly modulated by metabolites, we can focus on the status of these key compounds for the cell to link our elementary modules to cell physiology and environmental conditions. We applied and extended the internal/external/hybrid classification of sensing metabolites introduced by Martínez-Antonio and coauthors in [19] for transcription factors only to all kind of genetic regulators of *B. subtilis *considered in this study (T-boxes, etc.; see Additional file 8). A transcription factor is classified as "external" when the sensed signaling molecules is localized outside the cell, and "internal" when the sensed signal is an intracellular or endogenous molecule. A transcription factor classified as "hybride" senses a particular metabolite that, depending on growth conditions, is either synthetized in the cell or transported from outside.

Hybrid local regulators repress modules involved in anabolism while they activate modules involved in catabolism. External local regulators activate modules involved in the management of gluconeogenic carbon sources. Internal local regulators are involved both in anabolism and in catabolism. Those involved in carbohydrates catabolism activate their corresponding modules and have the same physiological role as hybrid local regulators. The remaining internal local regulators seem to be involved in anabolism and in the central carbon management.

Global regulators sense internal metabolite pool with the exception of CodY, which senses both internal and hybrid signals. Internal global regulators are mainly involved in catabolism as indicated in Results and in Additional file 8. Since an internal global regulator controls various modules, its metabolic effector should presumably be correlated to a physiological state, and allow the cell to take global decisions. The concentration of the CcpA effector FBP is correlated with the availability of glycolytic carbon sources [74] and indirectly manages the catabolite repression through CcpA. The glutamine concentration, sensed by TnrA, corresponds to the availability of a good ammonium source [42]. TnrA regulates the utilization of secondary nitrogen sources. Branched-chain amino acids, sensed by CodY, may reflect the availability of amino acids in the medium while GTP is strongly involved in ribosomes synthesis [75], sporulation and the so-called "stringent response" mechanism [76]. Moreover, the involvement of the three main global regulators (CcpA, TnrA, CodY) in the control of branched-chain amino acids synthesis reveals the putative critical role of these amino acids in the general management of the cell.

**Figure 4:**
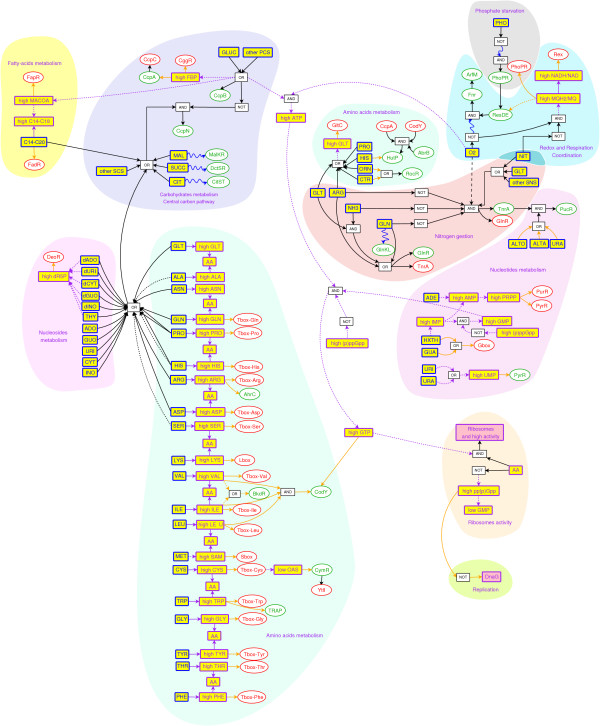
Regulation of the genetic network by metabolite pools of the following biological functions (central carbon pathway, redox and respiration coordination, phosphate starvation, nitrogen gestion, carbohydrates, nucleotide and amino acids metabolism, ribosome activity). SCS refers to secondary carbon source, PCS to primary carbon source, and SNS to secondary nitrogen source. The convention for the graphic representation is the same as for Figure 2.

The decomposition of the genetic and metabolic regulatory networks into functional modules associated to the nature of the sensed signals reveals how the cell coordinates the metabolic pathways in response to environmental changes as summarized in Figure 4. The graph highlights the conditions of activation of genetic regulators, according to both the availability of nutrients in the medium and the state (low/high) of internal or hybrid sensing metabolite.

More generally, the metabolic network and its associated complex regulations are key components of cell physiology. However, the metabolic modules have to be included in more general and complex regulation schemes (see Figure 4) where specific metabolite pools (like ATP, GTP, (p)ppGpp, etc.) play a key role in the coordination of the metabolic function with other essential biological processes. Perhaps the most interesting examples are the regulation of translation through the GTP pool and the stringent response [76] controlling both translation [75] and DNA replication [77] indirectly through the ppGpp signaling molecule.

## Conclusion

We presented the manually curated reconstruction of the genetic and metabolic regulatory network of the central metabolism of *Bacillus subtilis*. By introducing the notion of local and global regulation, we showed that the metabolic network can be broken down into sets of elementary functional modules. In addition, we developed a graphic representation of metabolic pathways based on these modules. Finally, we demonstrated that the structural analysis of metabolic network associated with the internal/external/hybrid classification of sensing genetic regulators could help to explain how the cell coordinates its metabolic network according to environmental changes. Basically, the genetic regulation represses the metabolic pathways involved in anabolism when the resource is available in the medium. The metabolic pathways involved in catabolism are induced by the availability of the resources in the medium only if a preffered resource is not available. These strategies minimize the cost of proteins production associated to the metabolic function.

## Methods

### Model reconstruction

There are few automatic tools allowing the reconstruction of genetic interaction networks and metabolic pathways, and they mostly build the metabolic network from the annotation of the genome and known metabolic pathways [78]. Manual validation is nevertheless necessary because of the discrepencies between the state of annotation and published knowledge. No such tools are available for the reconstruction of regulatory networks, which are mainly built from reports in the literature [79] and organism-specific databases.

The metabolic and genetic network was reconstructed from KEGG [80], PAREO (a relational database version of KEGG), SubtiList [81], and data in the literature. The regulatory networks were reconstructed from data in the literature, BRENDA for the control of enzymatic activities [82] and specific databases for *Bacillus subtilis*: DBTBS [83] and SPID [84]. SPID is a protein-protein interactions database, and DBTBS covers the transcription factors of *B. subtilis *and their regulons. However, DBTBS does not contain the metabolite effectors of transcription factors and some transcriptional regulation by early termination of transcription such as riboswitches. Therefore, some of the information can only be obtained from the literature.

The graphic representation of metabolic pathways has been developed with the DIA software [85].

### Description of regulatory networks

We have integrated the metabolic and genetic regulatory networks through (*i*) detailed conditions of transcription and translation of each gene, based on Boolean logic and modulation; (*ii*) detailed enzymatic control; (*iii*) conditions of activation of transcription factors, also based on Boolean logic. The Additional file 1 contains six layers. The first describes the organization of genes into operons. The second presents the abbreviations used both in this article and in the Excel file. We predicted the phenotypes of mutants in layer 3. Layer 4 describes the conditions of transcription of the transcription factors that have been included, and also the condition of activation of each transcription factor. A transcription factor is considered as "activated", when it is able to bind DNA. In layer 5, are described (*i*) the kinetic reactions of metabolic pathways; (*ii*) the genes (and operons) that encode the enzymes involved in reactions; (*iii*) the condition of transcription and translation of these genes; (*iv*) the regulation of enzymes; (*v*) the references for these regulations. The last layer contains the references. The description of transcriptional, translational and enzymatic regulations is presented below, through two examples: the anthranilate synthase (encoded by *trpE *and *trpG*) involved in the tryptophan synthesis, and glutamine phosphoribosylpyrophosphate amidotransferase (encoded by *purF*) involved in the purine synthesis.

#### Level of metabolite pools involved in regulation

By convention, the metabolite pools that are involved in either transcriptional, translational, enzymatic regulation or in the activation condition of a genetic regulator, are supposed to be high.

#### Transcription from a single promoter

A condition of transcription of a gene is associated to a promoter, through the following formalism based on Boolean logic and modulation:

**{ [ **RNAP binding]*modulation **} AND **(early termination of transcription)

The sequence between brackets describes the conditions for RNA polymerase binding. The modulation corresponds to transcription factors that modulate the transcription of a gene, and finally the conditions after the brackets describe the control by premature termination of transcription.

The *purF *gene belongs to the *pur *operon (see Additional file 5, Figure S43 and S50), which is transcribed from a *σ*^A ^promoter, and is repressed both by the transcriptional repressor PurR [86], and by early termination of transcription in the presence of guanine (GUA) or hypoxanthine (HXTH) [22]. The conditions of transcription are thus

**{ [*σ***^A^]*modI(PurR**) } AND NOT(**GUA OR HXTH**)**

where **modI **is an inhibitive modulation (**modA **indicates an activating modulation). In this expression, the modulation by PurR occurs when PurR is bound to DNA. PRPP prevents PurR binding to DNA [62]. PurR is active in the presence of PRPP, which is described as:

**PurR**_on _= PurR **AND NOT**(PRPP)

There is no known regulation of *purF *at the translational level. So, the corresponding column is empty. The gene *purF *encodes glutamine phosphoribosylpyrophosphate amidotransferase, which is inhibited by AMP, ADP+GMP, ADP+GDP and GMP+GDP [87]. Enzymatic regulations by metabolites are described as modulations, like those involving transcription (**modI, modA**). When several metabolites regulate an enzyme activity, they are separated by a comma.

**modI(**AMP, ADP+GMP, ADP+GDP, GMP+GDP**)**

When a cofactor is required for the enzymatic activity, it is indicated between brackets. For example, the succinate dehydrogenase requires two hemeB and [Fe-S] clusters, and is strongly inhibited by oxaloacetate (OAA), and weakly by fumarate (FUM), malate (MAL) and ATP. This regulation is written as

**[**Fe-S+2 HemeB]*modI(OAA, FUM, MAL, ATP**)**

#### Transcription of a gene from several promoters and description of complexes

If a gene is transcribed from several promoters, several conditions of transcription are included:

**[**condition 1**]OR [ **condition 2**]**

A complex, encoded by several genes, is modelled as a sum of genes

*gene1+ gene2+ gene3*

For example, the succinate dehydrogenase, encoded by *sdhA*, *sdhB *and *sdhC *is designated *sdhA*+*sdhB*+*sdhC *in the gene column. The *sdh *genes belong to the same operon, transcribed from a single promoter. Only one condition of transcription is included when genes that encode a complex are in operon. By contrast, *trpE *and *trpG *that encode the two subunits of the anthranilate synthase do not belong to the same operon. *trpE *belongs to the *trp *operon (Op92), and *trpG *belongs to an operon involved in folate synthesis (Op98). The *trpG *gene is also transcribed from a second constitutive promoter leading to the production of a monocistronic *trpG *transcript (indicated as trpG). The operons are also presented as a sum of operons in this case. Each part of the sum corresponds to one gene. For the anthranilate synthase:

$$\underset{trpE}{\underset{︸}{\text{Op}92}}+\underset{trpG}{\underset{︸}{\left(\text{Op}98,\text{trpG}\right)}}$$

The conditions of transcription for Op92, Op98, and the constitutive promoter upstream from *trpG*, are described as a sum of conditions

$$\underset{trpE}{\underset{︸}{\left\{\sigma^{\text{A}}\}ANDNOT(\text{TRAP}\right)}}+\underset{trpG}{\underset{︸}{\left([\sigma^{\text{A}}]OR[\sigma^{A}]\right)}}$$

When post-transcriptional regulations occur, they are indicated and represented as a sum of conditions. The TRAP protein prevents the translation of both Op92 (*trpE*) and Op98 (*trpG*), and translation from the trpG constitutive promoter always occurs (TRUE condition).

$$\underset{trpE}{\underset{︸}{NOT(\text{TRAP})}}+\underset{trpG}{\underset{︸}{\left(NOT(TRAP)ORTRUE\right)}}$$

### Predictions of gene deletion phenotypes

We used the constraints-based framework with Flux Balance Analysis (FBA, [88]) to predict effects of gene deletion on metabolism, with the incorporation of the genetic regulatory constraints proposed in [89] and applied in [90] on *Escherichia coli*. We determined, as proposed in [89] which enzymes are present according to their genetic regulation, and deduced the corresponding stoichiometry matrix to further apply FBA. However, slight modifications were introduced to integrate in the methodology the notions of: (*i*) "modulation" in genes transcription; and (*ii*) activation of genetic regulators (switch from "off" to "on") according to the presence of external metabolite pools.

(*i*) A gene whose transcription is only modulated by a transcription factor is considered present for the FBA analysis. For example, during growth on glucose, both CcpA and CcpN transcription factors are active. The *sucC-sucD *genes are kept for the FBA analysis since CcpA only modulates their transcription, while the *pckA *gene is not considered since CcpN strongly inhibits its transcription (see Additional file 1). Moreover, a gene whose transcriptional regulation is unknown is considered to be constitutively expressed (present) for the FBA analysis.

(*ii*) The concentration of intracellular metabolite pools cannot be quantitatively predicted since the model is only qualitative. A simplified and qualitative description for the evolution of metabolite effectors has been used, according to their hybrid/internal/external status [19]. Intracellular metabolite pools can be set to three levels: high if the metabolite is present in the medium and can be imported inside the cell (hybrid signal), medium if an intracellular synthesis flux exists (internal signal), null if the metabolite cannot be produced. The level of hybrid or external me-tabolite effectors leads to determine the state of hybrid or external genetic regulators given in Additional file 1 and 8, and so the presence of the controlled gene for the FBA analysis. Genes under the control of internal genetic regulators are systematically included in the analysis. Genetic regulators whose synthesis conditions are fulfilled and whose metabolite effectors are unknown are supposed to be in state "on".

As the model does not cover the complete metabolic network, we only consider for the predictions that all amino acids, branched-chain fatty-acids, nucleotides and deoxy-nucleotides are required to fullfil growth requirements.

The prediction of a gene deletion phenotype is determined in three steps.

1. All genes are first taken into account, except for the one whose deletion is tested. When a metabolite is present in the medium, set its maximal uptake flux to a non-zero value, and to zero otherwise.

2. Run the FBA analysis. Determine the null/medium/high level of metabolite pools involved in genetic regulations, according to the rules defined previously.

3. Evaluate the transcriptional rule for genes whose transcription condition is given by Boolean conditions (**AND**/**NOT**) and keep or eliminate genes in consequence. If some genes are eliminated, go back to step 2, else test the lethality of the phenotype. Only one iteration was sufficient in the case of our reconstructed metabolic network.

## Abbreviations

AA (amino acids), ADE (adenine), ADO (adenosine), ADP (adenosine diphosphate), ALA (alanine), ASN (asparagine), ASP (aspartate), ALTA (allantoate), ALTO (allantoin), AMP (adenosine monophosphate), ARG (arginine), ATP (adenosine triphosphate), BCAA (branched-chain amino acids), *B. subtilis *(*Bacillus subtilis*), CDS (coding sequence), CIT (citrate), C14-C18 (fatty acids with 14 to 18 carbons), C14-C20 (fatty acids with 14 to 20 carbons), CTR (citrulline), CYS (cysteine), CYT (cytidine), DAHP (3-deoxy-D-arabino-heptolosonate-7-phosphate), DNA (deoxyribonucleic acid), dADO (deoxyadenosine), dCYT (deoxycytidine), dGUO (deoxy-guanosine), dINO (deoxyinosine), dR5P (deoxyribose-5-phosphate), dURI (deoxyuridine), *E. coli *(*Escherichia coli*), FBP (fructose-1,6-biphosphate), FUM (fumarate), GDP (guanosine diphosphate), GLN (glutamine), GLT (glutamate), GLY (glycine), GMP (guanosine monophosphate), GTP (guanosine triphosphate), ppGpp (guanosine teraphosphate), pppGpp (guanosine pentaphosphate), GR (genetic regulator), GUA (guanine), GUO (guanosine), HIS (histidine), HXTH (hypoxanthine), ILE (isoleucine), IMP (inosine monophosphate), INO (inosine), LEU (leucine), LB medium (Luria-Bertani medium), LYS (lysine), MACOA (Malonyl-CoA), MAL (malate), MET (methionine), MQH2/MQ (menaquinone/menaquinol), mRNA (messenger RNA), NADH/NAD (nicotinamide adenine dinucleotide reduced/oxidized), NIT (nitrate), OAA (oxaloacetate), OAS (O-acetyl-L-serine), ORN (ornithine), O2 (dioxygen), PCS (primary carbon source), PHE (phenylalanine), PHO (phosphate), Plead (peptide leader), PRO (proline), PRPP (5-phosphoribosyl-1-pyrophosphate), PTS (phosphotransferase system), RBS (ribosome binding site), RNA (ribonucleic acid), RNAP (RNA polymerase), SAM (S-adenosyl-L-methionine), SER (serine), SCS (secondary carbon source), SNS (secondary nitrogen source), SUCC (succinate), TCA cycle (tricarboxylic acid cycle), TF (transcription factor), THR (threonine), THY (thymidine), TRP (tryptophan), TYR (tyrosine), UMP (uridine monophosphate), URA (urate), URI (uridine), UTP (uridine triphosphate), VAL (valine).

## Authors' contributions

AG carried out the reconstruction of *B. subtilis*, designed the graphic representations, participated in the local/global definition, identified the modules and drafted the manuscript. FBB developed the mathematical concepts upon which the module definition is based. IMV, PB, PN, and SA validated the reconstruction of *B. subtilis *and contributed to the writing of this manuscript. VF conceived of the study, participated in its design and coordination, and defined this manuscript. All authors read and approved the final manuscript.

## Supplementary Material

Additional File 1 Model of the central metabolism of *Bacillus sub-tilis*. This file contains the metabolic pathways description and their regulation, according to our formalism defined in the sections Results and Methods of this paper. Click here for file

Additional File 2 Review of the reconstruction of *Bacillus subtilis*. This file details the level of knowledge available for each metabolic pathway. The pathways which have been less characterized are explicitely mentioned. Click here for file

Additional File 3 Available information on the amino acid metabolism of *Bacillus subtilis*. This file details the level of knowledge available for each amino acid: genes and kinetic reactions, genetic and enzymatic regulation. Click here for file

Additional File 4 Predictions based on the reconstruction of *Bacillus subtilis*. The file details the phenotype analysis of *B. subtilis *and presents the potential utilization of amino acids as carbon or nitrogen sources. Click here for file

Additional File 5 Functional representation of the central metabolism of *Bacillus subtilis*. This file provides for each metabolic pathway: (*i*) a detailed representation of the kinetic reactions; (*ii*) the organization of genes in operon; (*iii*) a functional representation with the formalism detailed in the section Results of this paper. Click here for file

Additional File 6 Isoenzymes in metabolic pathways and their genetic regulation. This file details all known isoenzymes of the central metabolism of *Bacillus subtilis *and their genetic regulation. Click here for file

Additional File 7 Mathematical definition of module. The file presents the study of the steady-state behaviour of the main control structures identified in metabolic pathways. Click here for file

Additional File 8 Local versus global regulation of metabolic pathways. This file details the known genetic regulation of each metabolic pathway and precises (*i*) the "local/global" status of each genetic regulation; (*ii*) if the regulation is a modulation. Click here for file
