# Supplementary material for: Reconstruction and analysis of the genetic and metabolic regulatory networks of the central metabolism of Bacillus subtilis
Source: BMC Syst Biol. 2008 Feb 26;2:20. doi: 10.1186/1752-0509-2-20 (PMC2311275; doi:10.1186/1752-0509-2-20)
Supplement: Additional File 2 — Review of the reconstruction of Bacillus subtilis. This file details the level of knowledge available for each metabolic pathway. The pathways which have been less characterized are explicitely mentioned. [file 1752-0509-2-20-S2.doc]

# Review of the reconstruction

The main reason for this detailed reconstruction is to establish the first knowledge-based model of the major metabolic pathways of the bacterium *B. subtilis*: it corresponds to an accurate and unified view of current knowledge about this model Gram-positive bacterium. In this section, a detailed review of the current information available for each reconstructed pathway is presented.

***Central carbon pathway***. The central carbon pathway includes glycolysis, gluconeogenesis, the pentose phosphate pathway, the TCA cycle and the overflow pathway (acetoin and acetate syntheses). These pathways are probably the best studied [1-5], but much of the transcriptional regulation remains unknown, or has not been validated experimentally.

In *glycolysis* (see Figures 1 and 4 in Additional files 6)*,* only the regulation of the *gap* operon by CggR has been studied in detail [6]. The expression of the other genes (*pgi, pfk, fbaA and pykA*) does not seem to be regulated by glucose [7]; however, transcriptional studies including identification of promoters are lacking. Note that *fbaA* may be in an operon with *ywjH*, a gene of the pentose phosphate pathway: it would be interesting to validate this linkage.

The *pckA* and *gapB* genes involved in *gluconeogenesis* are repressed by CcpN in glycolysis conditions. How CcpN repression is released in gluconeogenesis conditions is unknown.

In the *pentose phosphate pathway* (see Figures 2 and 4 in Additional file 6), only the three 6-P-gluconate dehydrogenase isoenzymes (GntZ, YqjI, YqeC) of the oxidative branch have been characterized biochemically [8], and the transcriptional regulation of *ytqI* and *yqeC* is unknown. The non-oxidative branch of the pentose phosphate pathway has not been studied, either at the transcriptional or at the biochemical level. Only *tkt* appears to belong to the Spo0A regulon [9].

The enzymes of the *TCA cycle* (see Figures 3 and 4 in Additional file 6) have been well characterized [10-13]. Transcriptional regulation has been studied and the central role of CcpC for the tricarboxylic branch has been established. Citrate clearly prevents the binding of CcpC to the *citB* promoter region, leading to an increase of *citB* transcription. By contrast, the induction of *citZ*, the second target of CcpC, is weak in the same conditions, suggesting the presence of another effector [14]. Putative CcpA binding sites have been predicted upstream from genes involved in the dicarboxylic branch [15]. Transcriptome and proteome analyses indicate that these genes are repressed by glucose via CcpA [16,7]. Nothing is known concerning the regulation of genes encoding the pyruvate dehydrogenase complex.

CcpA and CodY are central to the regulation of genes of the *acetate synthesis pathway* [17] (see Figures 5 and 6 in Additional file 6). The *alsSD* operon is responsible for acetoin synthesis. The expression of this operon is induced by AlsR [18] and is repressed by Rex, in non-fermentative conditions (when the NADH/NAD ratio is low) [19]. The disruption of *alsR* prevents the expression of the operon, but the explicit binding of AlsR to DNA remains to be demonstrated.

***Aerobic, anaerobic respiration and fermentation.*** Aerobic respiration and the transition from aerobic to anaerobic respiration and fermentation have been extensively studied [20] (see Figures 10 and 11 in Additional file 6). The two-component system ResDE controls the transition between aerobic and anaerobic respiration but the signal(s) detected by this system remain(s) unknown. The transcriptional regulation of genes encoding the four NADH dehydrogenases is also uncharacterized, except for *ndh* controlled by Rex. The transcriptional regulation of the gene encoding the *aa3* oxidase active during the growth phase is also uncharacterized.

FNR is the key regulator of anaerobic respiration and fermentation via the control of ArfM synthesis. The mechanism of action of ArfM on its regulon remains to be determined. In addition, whether ArfM acts on *alsSD* and *lctEP* transcription directly or indirectly via Rex [21] has not been established. Not all the metabolic pathways of fermentation have been identified (see Figure 5 in Additional file 6). The reactions catalyzed by the two complexes involved in acetoin catabolism (*acoABCL* and *acuABC*) have not been determined. Nevertheless, AcoABCL seems to be the main acetoin dehydrogenase [22]. *B. subtilis* produces ethanol and 2,3-butanediol during the fermentation [22]. The pathways that lead to ethanol production are not well characterized. No acetaldehyde dehydrogenase that directly produces acetaldehyde from acetyl-coA has been found in *B. subtilis.* Acetaldehyde, the precursor of ethanol, seems to be produced from acetate by an aldehyde dehydrogenase, or by the acetoin dehydrogenase, encoded by the *acoABCL* operon*.* Five candidates (AldX, AldY, YcbD, YwdH and DhaS*)* for the aldehyde dehydrogenase are present in *B. subtilis*. The alcohol dehydrogenase, responsible for ethanol synthesis from acetaldehyde, may be encoded by four genes, *adhA, adhB, gbsB* and *yogA*. The 2,3-butanediol is produced from acetoin in one step, by a butanediol dehydrogenase encoded by an unknown gene or genes.

***Amino acid metabolism.*** Available knowledge about the transcriptional regulation and the control of the enzyme activities of amino acid synthesis and degradation pathways is presented in Additional file 3. Some pathways are uncharacterised to various extents: (*i*) all steps of the pathway (branched-chained amino acids degradation); (*ii*) the transcriptional regulation (chorismate, serine and some steps of threonine biosynthesis); (*iii*) both the transcriptional and enzymatic regulations (histidine, aspartate, alanine and proline biosynthesis, and serine, glycine and threonine degradation). It would be particularly interesting to focus on the amino-acids which are precursors for other essential metabolic components (alanine, aspartate for example) or are involved in adaptation to environmental changes (proline and osmotic stress). We discuss the regulation of these amino acids in detail below.

The regulation of genes involved in *aspartate and alanine synthesis* is unknown (see Figures 12 and 29 in Additional file 6). These amino-acids are produced from oxaloacetate or pyruvate, respectively, in one step by aminotransferases. The functions of the aminotransferases in *B. subtilis* have only been poorly described*.* An *alaT* mutant is nearly auxotroph for alanine confirming the key role of the AlaT aminotransferase in alanine synthesis [23]. However, no biochemical characterization of AlaT and no data for the transcriptional regulation of *alaT* are available. The aminotransferase AspB corresponds to the only aminotransferase involved in aspartate synthesis; the *aspB* mutant is auxotroph for aspartate [24]. The *aspB* gene seems to be constitutively transcribed [23]. Nevertheless, it is important to elucidate how the aspartate pool is controlled because aspartate is a precursor of lysine, threonine, methionine and peptidoglycan.

The intracellular concentration of *proline* increases from 16 mM to 700 mM [25] during osmotic stress (see Figure 23 in Additional file 6). In these conditions, proline synthesis (ProA, ProB, ProI, ProH, ProG, ProJ) and degradation (PutB, PutC) are presumably coordinated to prevent a futile cycle. The expression of *putBC* genes are repressed by CcpA [26]. The proline-specific T-boxes, found upstream from the *proBA* operon and the *proI* gene, remain to be validated [27]. The expression of *proH* and *proJ* is induced under osmotic stress conditions, but details of the mechanism of regulation are unknown.

***Nucleotide metabolism.*** Purine and pyrimidine syntheses have been extensively studied, and the pathways are well known both at the transcriptional and biochemical levels (see Figures 43, 44, 45, 46, 47, 49, 50 and 52 in Additional file 6). The regulation of the synthesis of di- and tri-phosphate nucleotides (NDP, NTP) from mono-phosphate nucleotides (NMP) remains an open question. ATP seems to be the main phosphate donor. The synthesis of NDP from NMP and ATP is catalyzed by several enzymes: Adk, Gmk, and Cmk catalyze ADP, GDP, and UDP synthesis from AMP, GMP, and UMP respectively. No transcriptional (or enzymatic) regulation has been reported for these genes (or enzymes). The synthesis of NTP from NDP and ATP is due to a unique nucleoside diphosphate kinase, encoded by ndk, whose transcriptional regulation is unknown. The purified enzyme exhibits different affinity (KM) for each NDP, which leads to GTP and UTP synthesis prior to CTP synthesis [28].

The regulation of the synthesis of deoxynucleotides from nucleotides by the complex encoded by the *nrdE* and *nrdF* genes is also unknown. No transcriptional information is available for the *nrdE* and *nrdF* genes. These genes are essential on LB medium [29], and their paralogues, *bnrdE* and *bnrdF*, do not encode a functional complex in these conditions. Either the complex might be active in other conditions (for example sporulation) or the function of the complex may be different.

***Fatty-acid metabolism.*** The initiation and elongation steps of both straight and branched-chain fatty acids have been identified (see Figures 54 and 56 in Additional file 6), except for the hydroxymyristoyl-(acp)-dehydratase, which may be encoded by the *ycsD* or *ywpB* genes, according to their annotation. FapR is involved in the transcriptional regulation of the corresponding genes. Nevertheless, the termination of fatty-acid synthesis and its control are still unknown. The oleoyl-[acyl-carrier-protein] hydrolase(s), which remove(s) the acyl-carrier protein from the fatty-acid, has (have) not been identified.

Steps of the degradation of straight and branched-chain fatty acids in *B. subtilis* have been proposed (see Figure 55 in Additional file 6); this degradation leads to the production of acetyl-CoA, propionyl-CoA and CO_2_. Two pathways for propionyl-CoA degradation are found in microorganisms. The first leads to succinate with the intermediate production of methyl-citrate and methyl-cis-aconitate. The second leads to succinyl-coA via the formation of methylmalonyl-CoA. In *B. subtilis*, the *mmgD*, *mmgE* and *yqiQ* genes may be involved in the first pathway. Most of the genes involved in the degradation of fatty-acids are only putative [30]. Recently, a transcription factor, *fadR* (*ysiA*) has been identified, and some genes of the FadR regulon have been renamed, according to their similarities with characterized genes [31]. Nevertheless, their functions have not been validated by biochemical study.

In conclusion, substantial amounts of information are available concerning metabolic pathways and their associated regulation. Few metabolic pathways are unknown, and in some cases, the *in vivo* functions of putative isoenzymes remain ambiguous. The general regulations of most metabolic pathways are documented, despite, some gene regulations within the pathways remaining unknown.

**References**

1. Steinmetz M: **Carbohydrate catabolism: pathways, enzymes, genetic regulation, and evolution.** In *Bacillus subtilis and other gram-positive bacteria: biochemistry, physiology, and molecular genetics*. Edited by Abraham L. Sonenshein et al.: ASM Press, Washington DC, USA; 1993:157-170.

2. Fortnagel P : **Glycolysis.** In *Bacillus subtilis and other gram-positive bacteria: biochemistry, physiology, and molecular genetics.* Edited by Abraham L. Sonenshein et al.: ASM Press, Washington DC, USA; 1993:171-180.

3. Hederstedt L: 1993. **The Krebs citric acid cycle.** In *Bacillus subtilis and other gram-positive bacteria: biochemistry, physiology, and molecular genetics.* Edited by Abraham L. Sonenshein et al.: ASM Press, Washington DC, USA; 1993:181-197.

4. Deutscher J, Galinier A, Martin-Verstraete I: **Carbohydrate uptake and metabolism.** In *Bacillus subtilis and its closest relatives: from genes to cells.* Edited by Abraham L. Sonenshein et al.: ASM Press, Washington DC, USA; 2001:129-150.

5. Sonenshein A: **The Krebs citric acid cycle.** In *Bacillus subtilis and its closest relatives: from genes to cells.* Edited by Abraham L. Sonenshein et al.: ASM Press, Washington DC, USA; 2001:151-162.

6. Doan T : **Etude fonctionnelle du génome de *Bacillus subtilis*: de nouvelles régulations transcriptionnelles du métabolisme central du carbone.** PhD thesis 2003, INRA Paris-Grignon.

7. Tobisch S, Zuhlke D, Bernhardt J, Stulke J, Hecker M: **Role of CcpA in regulation of the central pathways of carbon catabolism in *Bacillus subtilis*.** *J Bacteriol* 1999, **181**(22):6996–7004.

8. Zamboni N, Fischer E, Laudert D, Aymerich S, Hohmann H, Sauer U: **The *Bacillus subtilis* *yqjI* gene encodes the NADP+-dependent 6-p-gluconate dehydrogenase in the pentose phosphate pathway.** *J Bacteriol* 2004, **186**(14):4528–4534.

9. Molle V, Fujita M, Jensen S, Eichenberger P, Gonzalez-Pastor J, Liu J, Losick R: **The spo0A regulon of *Bacillus subtilis*.** *Mol Microbiol* 2003, **50**(5):1683–1701.

10. Flechtner VR, Hanson RS: **Coarse and fine control of citrate synthase from Bacillus subtilis.** *Biochim Biophys Acta* 1969, **184**(2):252-62.

11. Hanson S, Cox D: **Effect of different nutritional conditions on the synthesis of tricarboxylic acid cycle enzymes.** *J Bacteriol* 1967, **93**(6):1777–1787.

12. Diesterhaft M, Freese E: **Role of pyruvate carboxylase, phosphoenolpyruvate carboxykinase, and malic enzyme during growth and sporulation in *Bacillus subtilis*.** *J Biol Chem* 1973, **248**(17):6062–6070.

13. Ohne M: **Regulation of the dicarboxylic acid part of the citric acid cycle in *Bacillus subtilis*.** *J Bacteriol* 1975, **122**(1):224–234.

14. Jourlin-Castelli C, Mani N, Nakano M, Sonenshein A: **CcpC, a novel regulator of the LysR family required for glucose repression of the *citB* gene in *Bacillus subtilis*.** *J Mol Biol* 2000, **295**(4):865–878.

15. Miwa Y, Nakata A, Ogiwara A, Yamamoto M, Fujita Y: **Evaluation and characterization of catabolite-responsive elements (cre) of *Bacillus subtilis*.** *Nucleic Acids Res* 2000, **28**(5):1206–1210.

16. Moreno M, Schneider B, Maile R, Weyler W, Saier MJ: **Catabolite repression mediated by the CcpA protein in *Bacillus subtilis*: novel modes of regulation revealed by whole-genome analyses.** *Mol Microbiol* 2001, **39**(5):1366–1381.

17. Shivers R, Dineen S, Sonenshein A: **Positive regulation of *Bacillus subtilis* *ackA* by CodY and CcpA: establishing a potential hierarchy in carbon flow.** *Mol Microbiol* 2006, **62**(3):811–822.

18. Renna MC, Najimudin N, Winik LR, Zahler SA: **Regulation of the *Bacillus subtilis* alsS, alsD, and alsR genes involved in post-exponential-phase production of acetoin.** *J Bacteriol* 1993, **175**(12):3863-3875.

19. Reents H, Munch R, Dammeyer T, Jahn D, Hartig E: **The Fnr regulon of *Bacillus subtilis*.** *J Bacteriol* 2006, **188**(3):1103-1112.

20. Nakano M Zuber P: **Anaerobic growth of a strict aerobe (*Bacillus subtilis*).** *Annual Rev Microbiol* 1998, **52**:165–190.

21. Marino M, Cruz-Ramos H, Hoffmann T, Glaser P, Jahn D : **Modulation of anaerobic energy metabolism of *Bacillus subtilis* by ArfM (YwiD).** *J Bacterioly* 2001, **183**(23):6815–6821.

22. Cruz Ramos H, Hoffmann T, Marino M, Nedjari H, Presecan-Siedel E, Dreesen O, Glaser P, Jahn D: **Fermentative metabolism of *Bacillus subtilis*: physiology and regulation of gene expression.** *J Bacteriol* 2000, **182**(11):3072-3080.

23. Belitsky BR: **Biosynthesis of amino acids of the glutamate and aspartate families, alanine and polyamines**. In *Bacillus subtilis and its closest relatives: from genes to cells.* Edited by Abraham L. Sonenshein et al.: ASM Press, Washington DC, USA; 2001:203-231.

24. Iijima T, Diesterhaft MD, Freese E: **Sodium effect of growth on aspartate and genetic analysis of a *Bacillus subtilis* mutant with high aspartase activity.** *J Bacteriol* 1977, **129**(3):1440–1447.

25. Bremer E: 2001. **Adaptation to changing osmolarity.** In *Bacillus subtilis and its closest relatives: from genes to cells.* Edited by Abraham L. Sonenshein et al.: ASM Press, Washington DC, USA; 2001: 385-391.

26. Lulko A, Buist G, Kok J, Kuipers O: **Transcriptome analysis of temporal regulation of carbon metabolism by CcpA in *Bacillus subtilis* reveals additional target genes.** *J Mol* *Microbiol Biotechnol* 2007, **12**(1-2):82–95.

27. Chopin A, Biaudet V, Ehrlich D: **Analysis of the *Bacillus subtilis* genome sequence reveals nine new T-box leaders.** *Mol Microbiol* 1998, **29**(2):662.

28. Sedmak J, Ramaley R: **Purification and properties of *Bacillus subtilis* nucleoside diphosphokinase.** *J Biol Chem,* 1971, **246**(17):5365–5372.

29. Kobayashi K, Ehrlich S, Albertini A, Amati G, Andersen K, Arnaud M, Asai K, Ashikaga S, Aymerich S, Bessieres P, et al*.*: **Essential *Bacillus subtilis* genes.** *Proc Natl Acad Sci USA* 2003, **100**(8):4678–4683.

30. Koburger T, Weibezahn J, Bernhardt J, Homuth G, Hecker M: **Genome-wide mRNA profiling in glucose starved *Bacillus subtilis* cells.** *Mol Genet Genomics* 2005, **274**(1):1–12.

31. Matsuoka H, Hirooka K, Fujita Y: **Organization and function of the YsiA regulon of *Bacillus subtilis* involved in fatty acid degradation.** *J Biol Chem* 2007, **282**(8):5180–5194.
