# Supplementary material for: Reconstruction and analysis of the genetic and metabolic regulatory networks of the central metabolism of Bacillus subtilis
Source: BMC Syst Biol. 2008 Feb 26;2:20. doi: 10.1186/1752-0509-2-20 (PMC2311275; doi:10.1186/1752-0509-2-20)
Supplement: Additional File 4 — Predictions based on the reconstruction of Bacillus subtilis. The file details the phenotype analysis of B. subtilis and presents the potential utilization of amino acids as carbon or nitrogen sources. [file 1752-0509-2-20-S4.doc]

**Predictions based on the reconstruction of *Bacillus subtilis***

The model can be used (*i*) to predict the phenotypes associated to genes deletion and (*ii*) to check if the known management of various carbon and nitrogen sources by *B. subtilis* can be deduced from the analysis of the genetic regulations. The recent high-throughput phenotypic testing in [1] and literature have been used to test the predictions.

**I. Predictions of gene deletion phenotypes**

***Prediction of gene deletion phenotypes***.

In a minimal medium containing glucose, ammonium and sulfate as sole carbon, nitrogen and sulfur sources, respectively, 153 genes involved in metabolic pathways and three genes encoding regulators (i.e. 25% of the genes included in our model) are predicted to be required for growth (see Additional file 1). The three regulatory proteins are GltC, CysL (formerly YwfK) and RtpA (formerly YczA). GltC and CysL directly activate the transcription of genes required for growth in minimal medium. GltC activates the transcription of the *gltAB* operon, which encodes the glutamate synthase [2]. CysL activates the transcription of the *cysJI* operon, which encodes the sulfite reductase catalysing the last step of the sulfate assimilation pathway [3]. RtpA sequesters the TRAP protein during tryptophan starvation, and is necessary to switch off TRAP-dependent repression of tryptophan synthesis during starvation of this amino-acid [4]. Typically, these predictions cannot be inferred from the study of the stoichiometric network alone.

The exact composition of LB (rich) medium is difficult to evaluate. As it contains yeast extracts and peptides, we consider that glucose, all the amino-acids, all the nucleobases (adenine, guanine…) and the (deoxy)-nucleosides, acetate and branched short-chain α-keto acids are available. In this rich medium, 56 genes (9% of all genes included) are predicted to be required for growth.

***Validation of model predictions.*** We compared these predictions to known phenotypes described in the work of Fischer and Sauer [5] (minimal medium), of Kobayashi et al. [6] (rich medium), and elsewhere (see Additional file 1).

*In minimal medium*: Among the 156 genes that were predicted to be required for growth, experiments are available for only 30 genes, whose lethal phenotypes have been confirmed. Among the 456 genes which are predicted to be inessential for growth, 100 genes have been tested experimentally and predictions were false for three (*cdd*, *trxB*, and *ptsH*).

*In rich medium*: the predictions were directly compared to the results of [6]. Twelve genes (*trxB*, *yumC, odhB,* *pdhA, guaB, pyrG, tyrS, yycF, pyrG, cmk, nrdE, nrdF, nrdI*) that were predicted to be dispensable are actually essential. By contrast, two genes (*ndk*, *dapE*) are dispensable, although predicted to be essential.

The discrepancies between the predictions and the experimental data in rich and minimal medium can be explained in different ways. When the genome encodes isoenzymes, it is often predicted that the deletion of one gene can be compensated by the synthesis of an isoenzyme. Indeed our model assumes that the transcription of genes with unknown regulation is constitutive (see Methods). However, this does not explain the observed requirement for growth of *trxB*, *yumC* and *tyrS* mutants (see Additional file 4), as well as that of the *ptsH* mutant, which prevents glucose transport by the main glucose transporter PtsG and should be compensated by two potential isoenzymes (GlcP, GlcU). Alternative causes for discrepencies could be: (*i*) a gene could have several biological functions, one of them being essential (*odhB,* *pdhA, cdd*), (*ii*) the existence of a paralogue or of a functional analog could compensate the gene disruption (*ndk*,*dapE*), (*iii*) the hypotheses on the LB medium composition are wrong. Typically the phenotypes associated to *guaB, pyrG, cmk, nrdE, nrdF, nrdI* deletions could be explained by the paucity of the LB medium in guanine and (deoxy)-cytidine [6]. Finally, the YycF transcription factor controls the expression of essential genes [7] that have not been included in our model.

**II. Predictions of the utilization of carbon and nitrogen sources**

Not all the 20 amino-acids can be used as nitrogen sources or carbon sources in *B. subtilis*: only L-alanine, L-aspartate, L-asparagine, L-arginine, L-ornithine, L-glutamate, L-threonine, L-glutamine, L-histidine, L-isoleucine, L-proline, and L-valine can be used as nitrogen sources [8]. L-arginine, L-glutamate and L-glutamine catabolism is controlled at the transcriptional level by TnrA [9,2,10] and histidine degradation is regulated by CodY [11]. Both CodY and TnrA are involved in valine and isoleucine catabolism [12]. In conclusion, the known transcriptional regulations of amino acid degradation as nitrogen sources are in agreement with physiological data.

The exhaustive list of amino acids that can be used as carbon sources is not available. Published data and predictions suggest that L-aspartate, L-glutamine, L-glutamate, L-ornithine, and L-proline are used as carbon sources [13,14]. Our model predicts that L-alanine, L-serine, L-histidine, L-arginine, L-asparagine, L-threonine, L-isoleucine, and L-valine can be used; the corresponding degradation pathways exist. Moreover, CcpA directly regulates the degradation of L-histidine [15], and indirectly, L-arginine catabolism through σ^L^ repression [16,17]. CcpA also indirectly represses BCAA degradation because the *bkd* operon is transcribed from a σ^L^-specific promoter [12]. Moreover, the transcription of the *bkd* operon is activated by the local regulator BkdR in the presence of L-isoleucine and L-valine, the two metabolite effectors [12]. In consequence, we predict that L-isoleucine and L-valine can also be used as alternative carbon sources. If a L-leucine transporter exists, L-leucine may be used if L-isoleucine or L-valine is also present. L-threonine, with pyruvate, is the precursor of L-isoleucine. So if L-isoleucine could be used as carbon source, L-threonine could be used as well. Indeed, at least one L-threonine transporter must exist, because L-threonine can be used as a nitrogen source. The existence of an operon, which includes the genes involved both in L-aspartate and L-asparagine degradation [18], suggests that L-asparagine, like L-aspartate, can be used as a carbon source.

Recently the ability of *Bacillus subtilis* to grow on various carbon, nitrogen, sulphur and phosphorus sources has been tested using Biolog’s Phenotype MicroArrays technology [19] by Oh et al. [1]. Growth on L-asparagine, L-serine, L-threonine, and L-alanine as carbon source has been confirmed. L-histidine and L-arginine have not been tested. By contrast, *B. subtilis* is not able to grow on any of the branched-chain amino acids, contrary to our predictions or those provided by Oh et al. [1]. This is surprising, in particular since L-threonine can be used as carbon source. As the different steps of the degradation pathway and their regulation are not yet characterized in *B. subtilis*, one can conclude that either (*i*) *B. subtilis* cannot grow on branched-chain amino acids but recent results suggest the contrary [20] or (*ii*) either one of these steps should be limiting under the conditions tested in [1]. Perhaps a mixture of branched-chain amino acids instead of a unique has to be present to induce the degradation pathway.

**References**

1. Oh YK, Palsson BO, Park SM, Schilling CH, Mahadevan R: **Genome-scale reconstruction of metabolic network in *Bacillus subtilis* based on high-throughput phenotyping and gene essentiality data.** *J Biol Chem* 2007, in press.

2. Belitsky B, Wray LJ, Fisher S, Bohannon D, Sonenshein A: **Role of TnrA in nitrogen source-dependent repression of *Bacillus subtilis* glutamate synthase gene expression.** *J Bacteriol* 2000, **182**:5939–5947.

3. Guillouard I, Auger S, Hullo MF, Chetouani F, Danchin A, Martin-Verstraete I: **Identification of *Bacillus subtilis* CysL, a regulator of the cysJI operon, which encodes sulfite reductase.** *J Bacteriol* 2002, **184**:4681-4689.

4. Gollnick P, Babitzke P, Antson A, Yanofsky C: **Complexity in regulation of tryptophan biosynthesis in *Bacillus subtilis*.** *Annu Rev Genet* 2005, **39**:47–68.

5. Fischer E, Sauer U: **Large-scale *in vivo* flux analysis shows rigidity and suboptimal performance of *Bacillius subtilis* metabolism.** *Nat Genet* 2005, **37**:636–640.

6. Kobayashi K, Ehrlich S, Albertini A, Amati G, Andersen K, Arnaud M, Asai K, Ashikaga S, Aymerich S, Bessieres P, et al: **Essential Bacillus subtilis genes.** *Proc Natl Acad Sci USA* 2003, **100**:4678–4683.

7. Bisicchia P, Noone D, Lioliou E, Howell A, Quigley S, Jensen T, Jarmer H, Devine KM: **The essential YycFG two-component system controls cell wall metabolism in *Bacillus subtilis*.** *Mol Microbiol* 2007, **65**:180-200.

8. Fisher SH: **Utilization of amino acids and other nitrogen-containing compounds.** In *Bacillus subtilis and other gram-positive bacteria: biochemistry, physiology, and molecular genetics*. Edited by Abraham L. Sonenshein et al.: ASM Press, Washington DC, USA; 1993:221-228.

9. Fisher S, Wray L: ***Bacillus subtilis* 168 contains two differentially regulated genes encoding L-asparaginase.** *J Bacteriol* 2002, **184**:2148–2154.

10. Wray LJ, Ferson A, Rohrer K, Fisher S: **TnrA, a transcription factor required for global nitrogen regulation in *Bacillus subtilis*.** *Proc Natl Acad Sci USA* 1996, **93**:8841–8845.

11. Fisher S, Rohrer K, Ferson A: **Role of CodY in regulation of the *Bacillus subtilis* hut operon.** *J Bacteriol* 1996, **178**:3779–3784.

12. Débarbouillé M, Gardan R, Arnaud M, Rapoport G : **Role of bkdR, a transcriptional activator of the sigL-dependent isoleucine and valine degradation pathway in *Bacillus subtilis*.** *J* *Bacteriol* 1999, **181**:2059–2066.

13. Blencke H, Reif I, Commichau F, Detsch C, Wacker I, Ludwig H, Stulke J: **Regulation of *citB* expression in *Bacillus subtilis*: integration of multiple metabolic signals in the citrate pool and by the general nitrogen regulatory system.** *Arch Microbiol* 2005, **185**:136–146.

14. Belitsky B, Kim H, Sonenshein A: **CcpA-dependent regulation of *Bacillus subtilis* glutamate dehydrogenase gene expression.** *J Bacteriol* 2004, **186**:3392–3398.

15. Wray LJ, Pettengill F, Fisher S: **Catabolite repression of the *Bacillus subtilis hut* operon requires a cis-acting site located downstream of the transcription initiation site.** *J Bacteriol* 1994, **176**:1894–1902.

16. Choi SK, Saier MH Jr: **Regulation of *sigL* expression by the catabolite control protein CcpA involves a roadblock mechanism in *Bacillus subtilis*: potential connection between carbon and nitrogen metabolism.** *J Bacteriol* 2005, **187**:6856-6861.

17. Calogero S, Gardan R, Glaser P, Schweizer J, Rapoport G, Debarbouille M: **RocR, a novel regulatory protein controlling arginine utilization in *Bacillus subtilis*, belongs to the NtrC/NifA family of transcriptional activators.** *J Bacteriol* 1994*,* **176**:1234-1241.

18. Sun DX, Setlow P: **Cloning, nucleotide sequence, and expression of the *Bacillus subtilis* *ans* operon, which codes for L-asparaginase and L-aspartase.** *J Bacteriol* 1991, **173**:3831-3845.

19. Bochner BR Gadzinski P, Panomitros E: **Phenotype microArrays for high-throughput phenotypic testing and assay of gene function.** *Genome Res* 2001, **11**:1246-1255.

20. Koburger T, Weibezahn J, Bernhardt J, Homuth G, Hecker M: **Genome-wide mRNA profiling in glucose starved *Bacillus subtilis* cells.** *Mol Genet Genomics* 2005, **274**:1–12.
