# Supplementary material for: Reconstruction and analysis of the genetic and metabolic regulatory networks of the central metabolism of Bacillus subtilis
Source: BMC Syst Biol. 2008 Feb 26;2:20. doi: 10.1186/1752-0509-2-20 (PMC2311275; doi:10.1186/1752-0509-2-20)
Supplement: Additional File 5 — Functional representation of the central metabolism of Bacillus subtilis. This file provides for each metabolic pathway: (i) a detailed representation of the kinetic reactions; (ii) the organization of genes in operon; (iii) a functional representation with the formalism detailed in the section Results of this paper. [file 1752-0509-2-20-S5.pdf]

Functional representation of metabolic pathways

Table of contents

***1. CENTRAL CARBON METABOLISM* 3**

***2. AEROBIC, ANAEROBIC RESPIRATION AND FERMENTATION* 13**

***3. AMINO ACIDS METABOLISM* 16**

3.1) ALANINE 17
3.2) GLYCINE 17
3.3) ISOLEUCINE, VALINE AND LEUCINE 19
3.4) METHIONINE AND CYSTEINE 22
3.5) PROLINE 27
3.6) PHENYLALANINE, TYROSINE AND TRYPTOPHAN 29
3.7) ASPARTATE AND ASPARAGINE 33
3.8) GLUTAMATE AND GLUTAMINE 35
3.9) ARGININE 37
3.10) HISTIDINE 39
3.11) LYSINE AND THREONINE 42
3.12) SERINE 45

***4. NUCLEOTIDES METABOLISM* 46**

4.1) SYNTHESIS OF NUCLEOTIDES 47
4.2) PURINES AND PYRIMIDINES SALVAGE PATHWAY 50

***5. FATTY-ACIDS METABOLISM* 57**

1. Central carbon metabolism

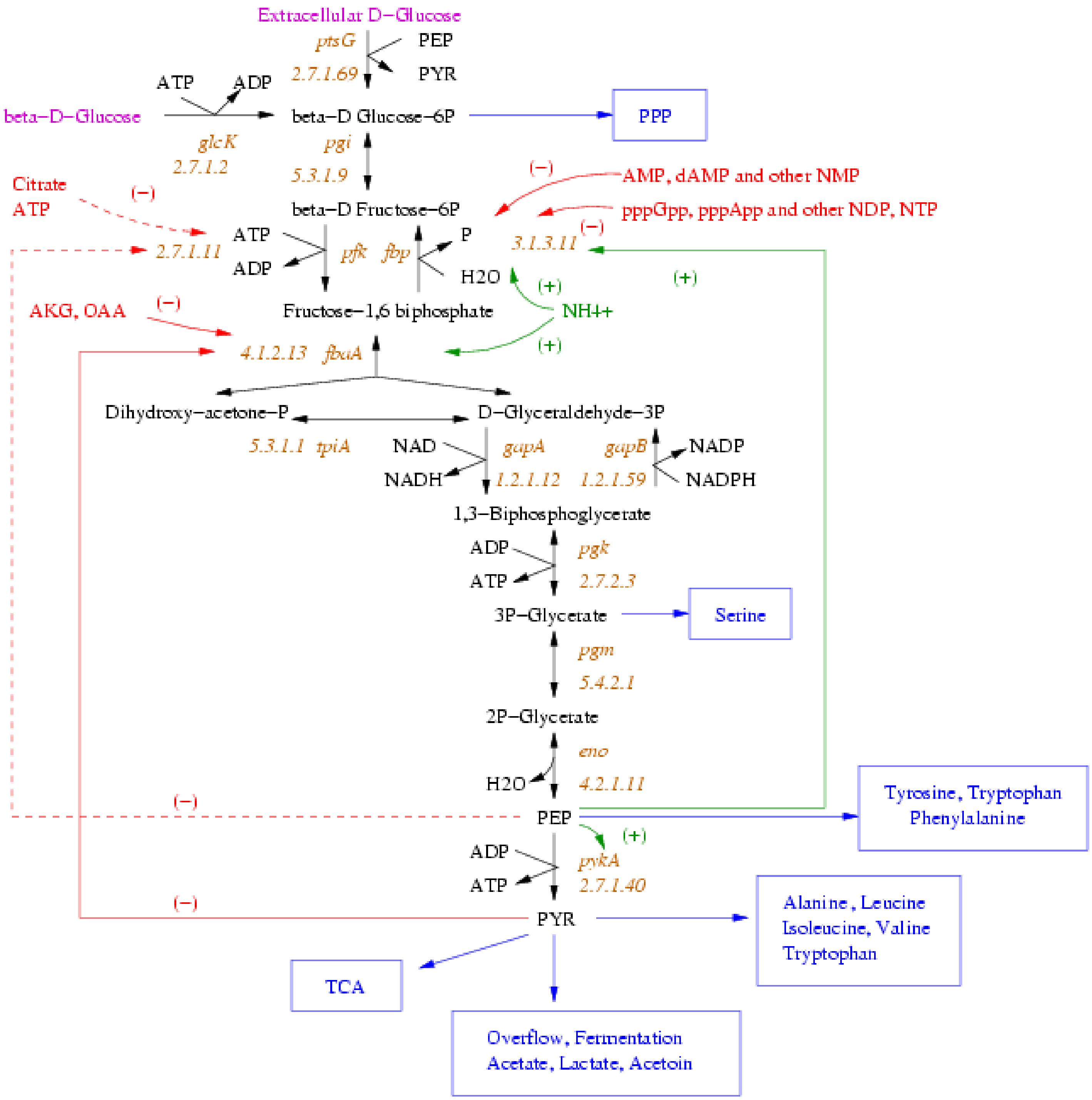

Figure 1: Glycolysis pathway

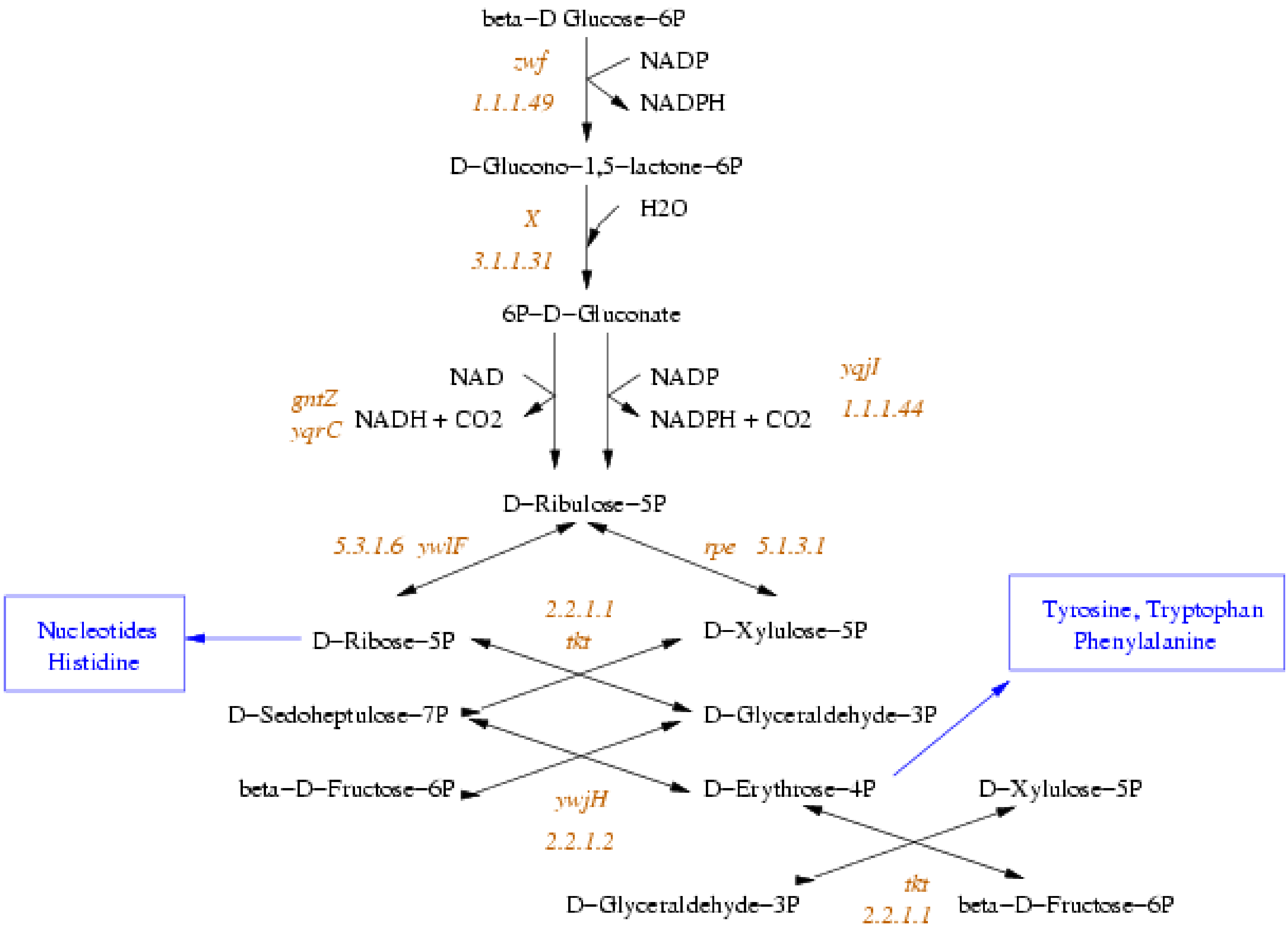

Figure 2: Pentose phosphate pathway

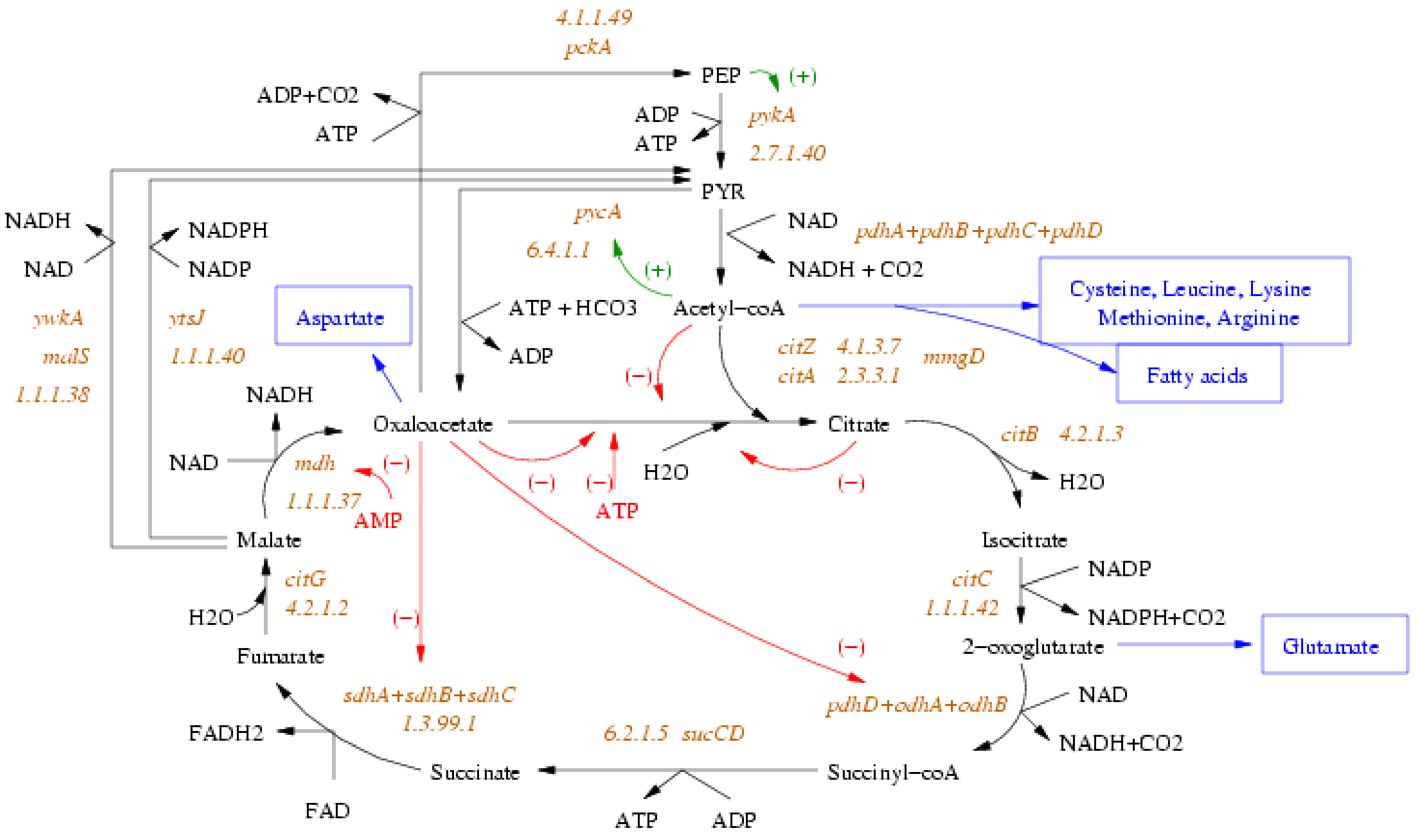

Figure 3: TCA cycle

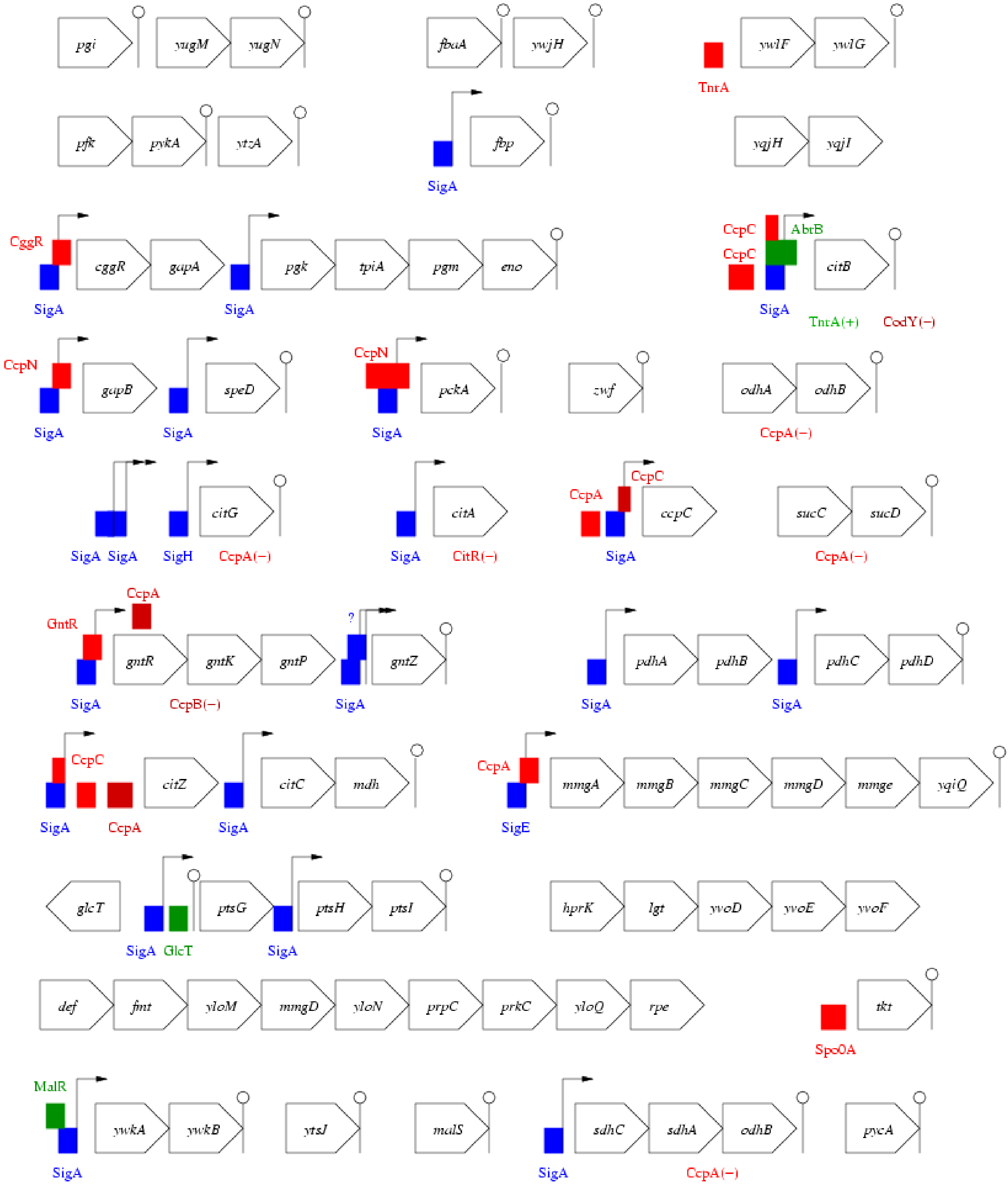

No information on: *glcK, yqeC*

<u>Figure 4: Operons involved in glycolysis, PPP and TCA cycle pathways</u>

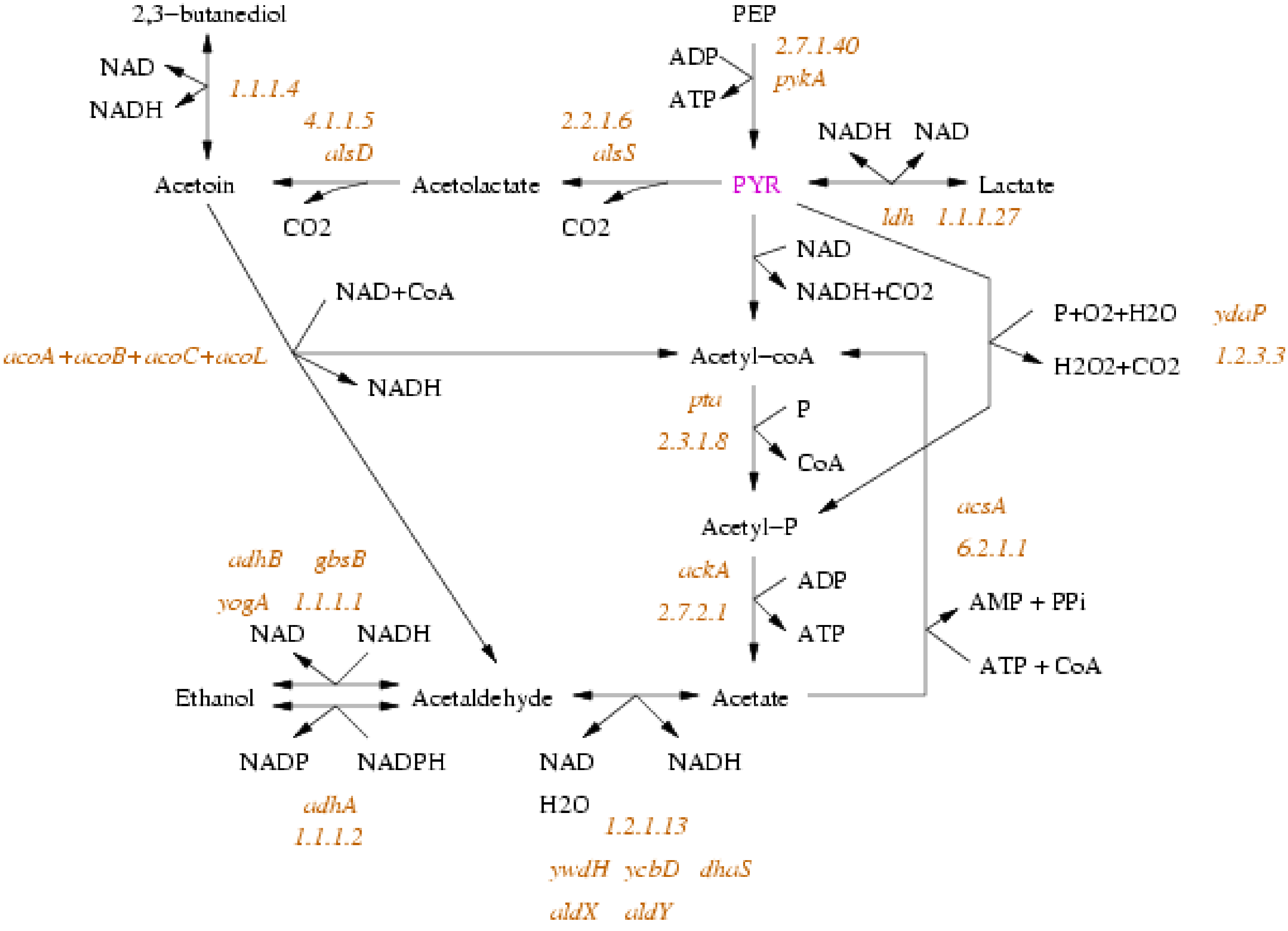

Figure 5: Overflow pathway and fermentation

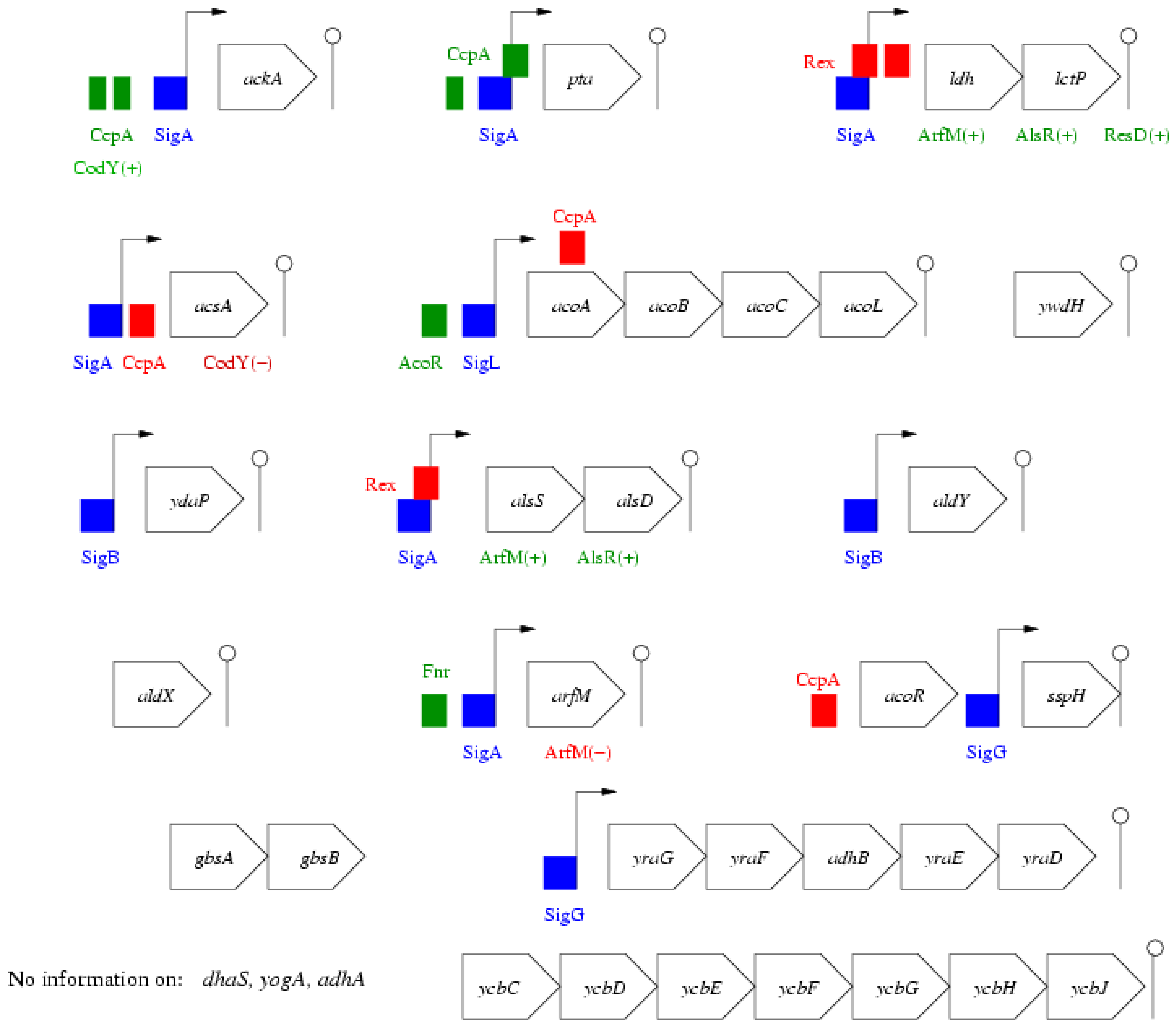

Figure 6: Operons involved in overflow and fermentation pathways

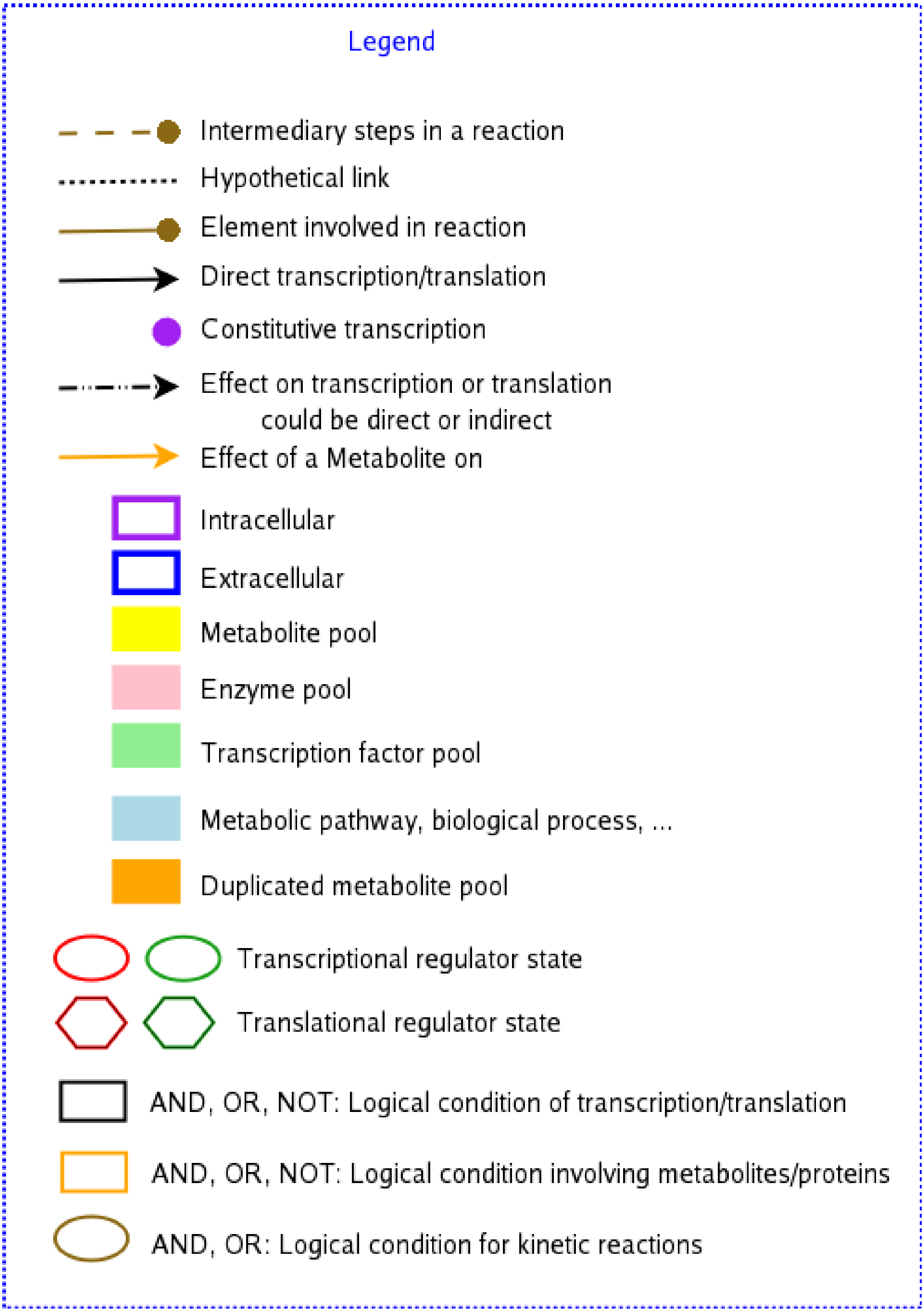

Figure 7: Legend

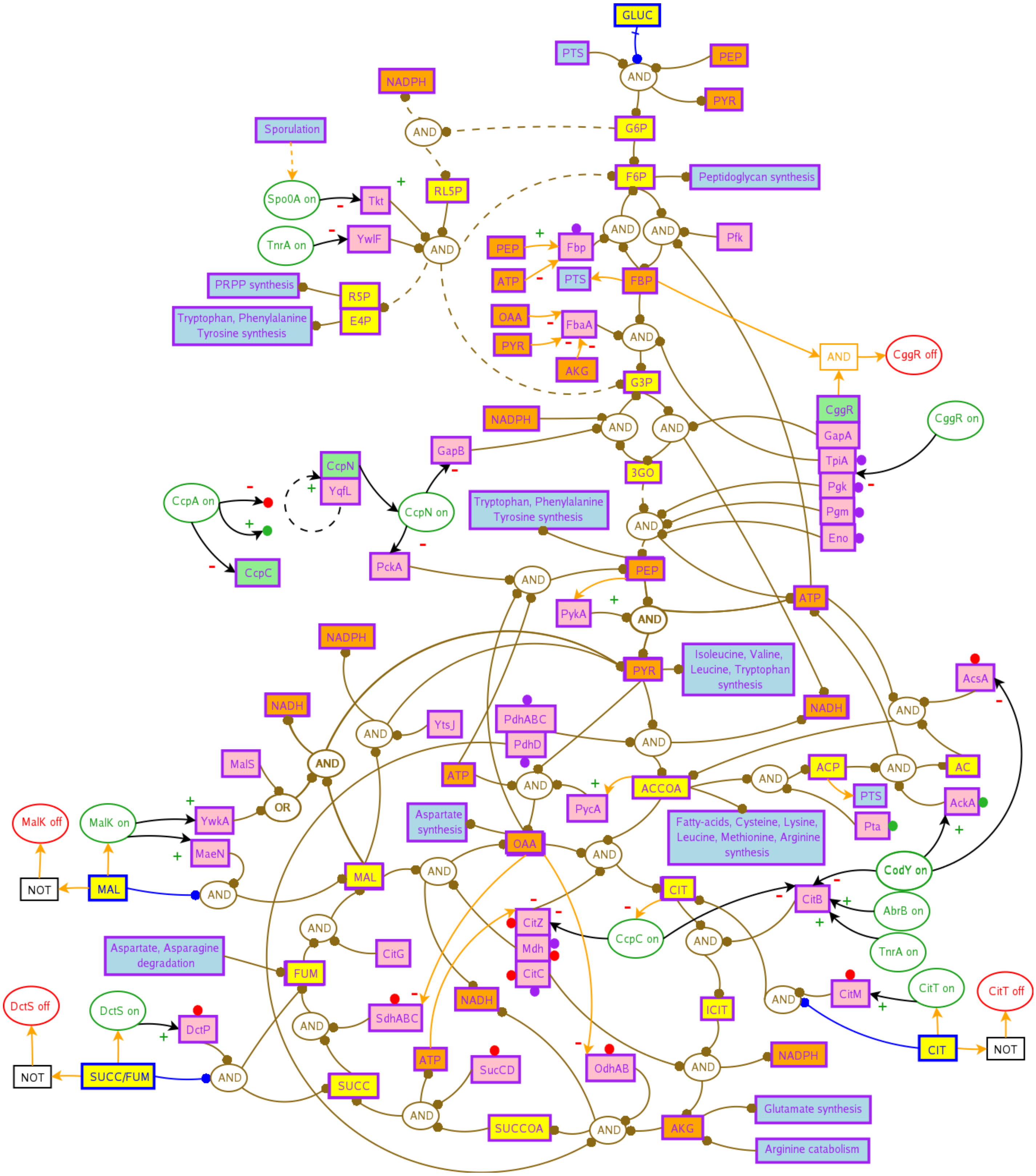

Figure 8: Regulation of the central carbon metabolism

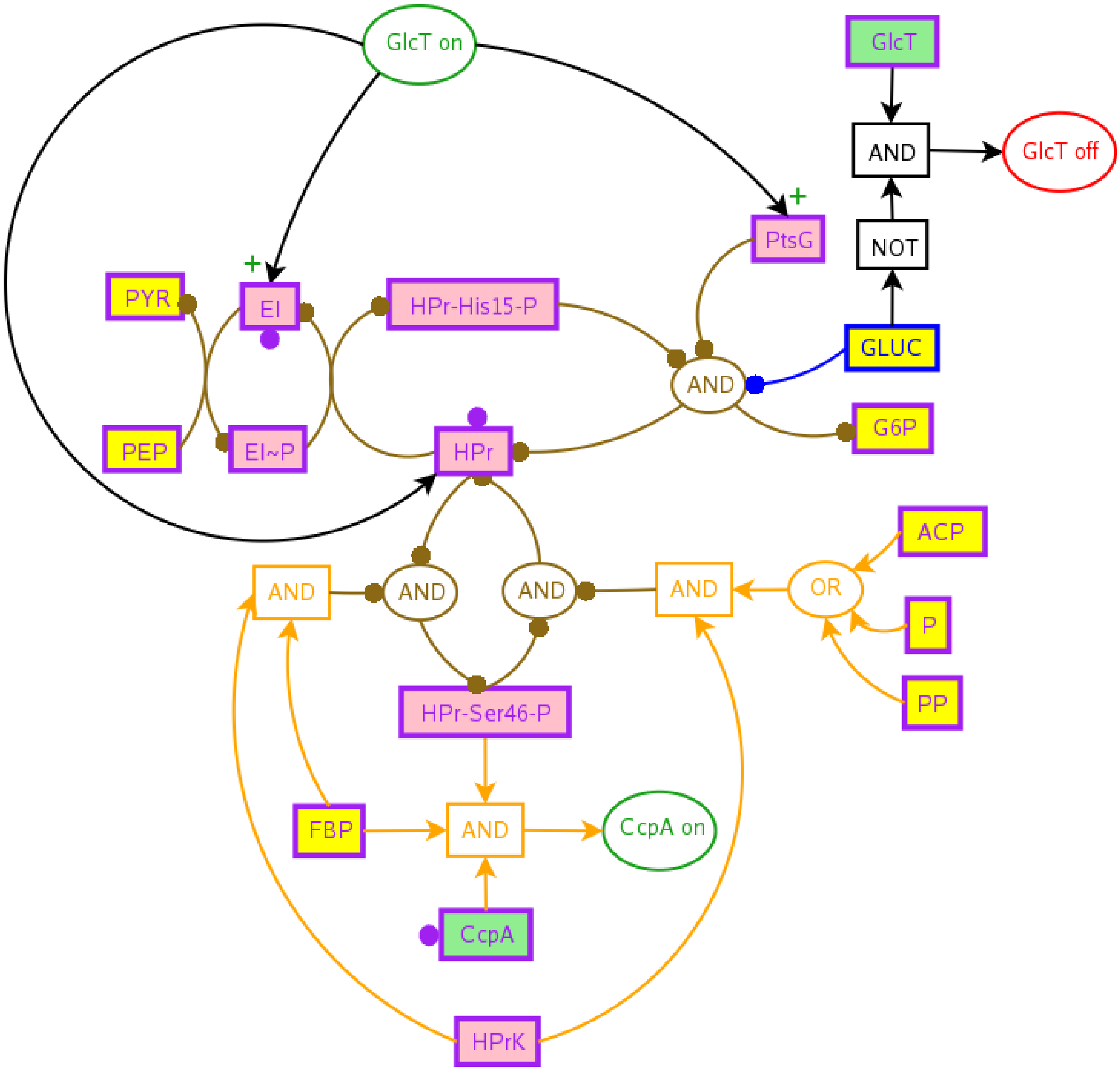

Figure 9: Regulation of the glucose PhosphoTransferaseSystem

2. Aerobic, anaerobic respiration and fermentation

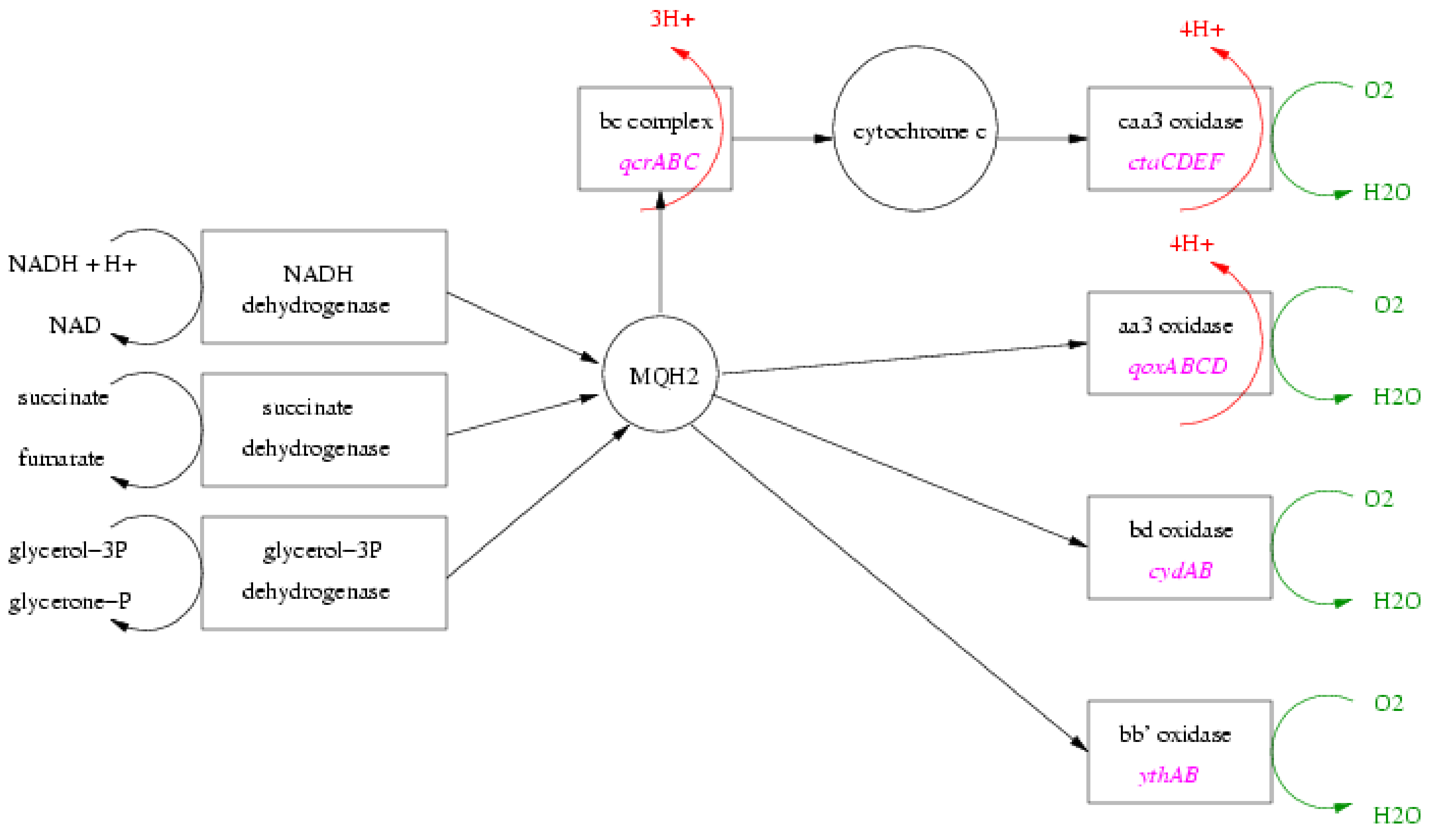

Figure 10: Aerobic respiration

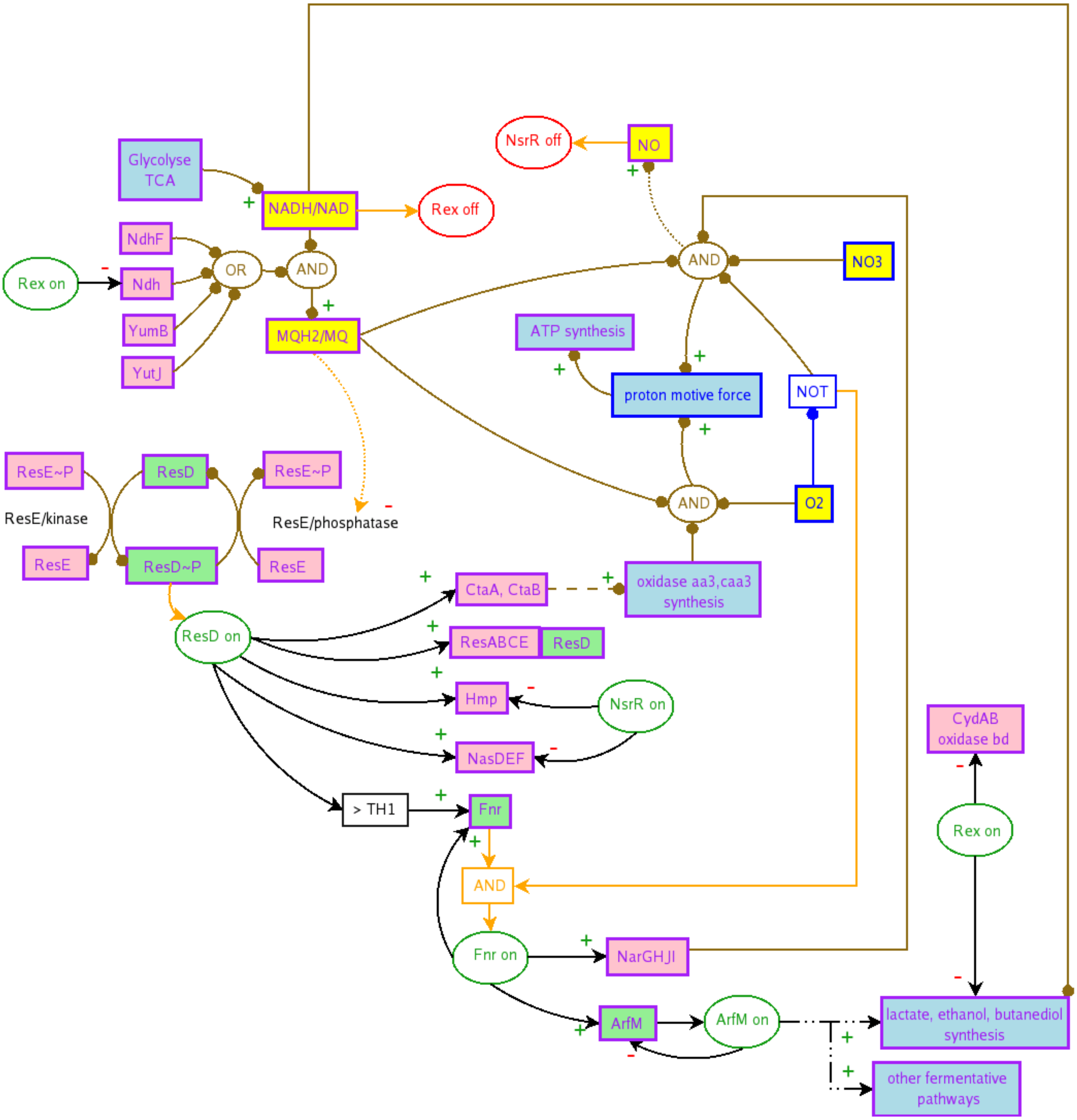

Figure 11: Regulation of the transition from aerobic to anaerobic respiration and fermentation

3. Amino acids metabolism

3.1) Alanine

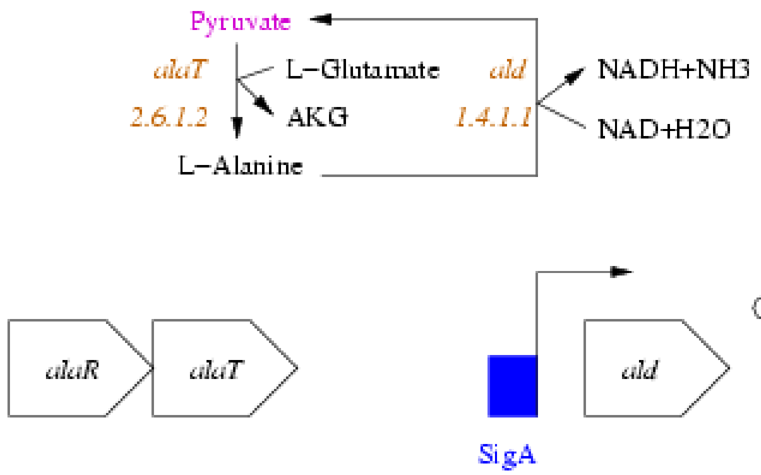

Figure 12: Alanine metabolism

3.2) Glycine

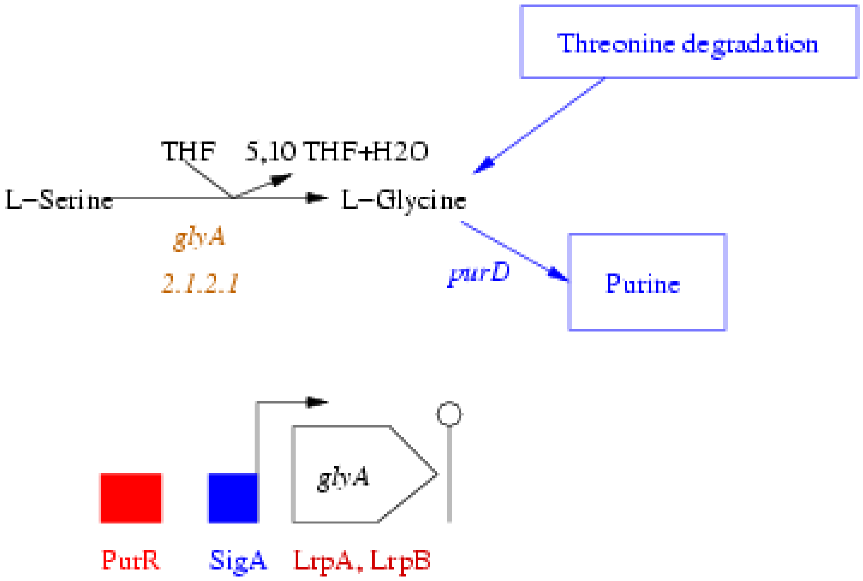

Figure 13: Glycine metabolism

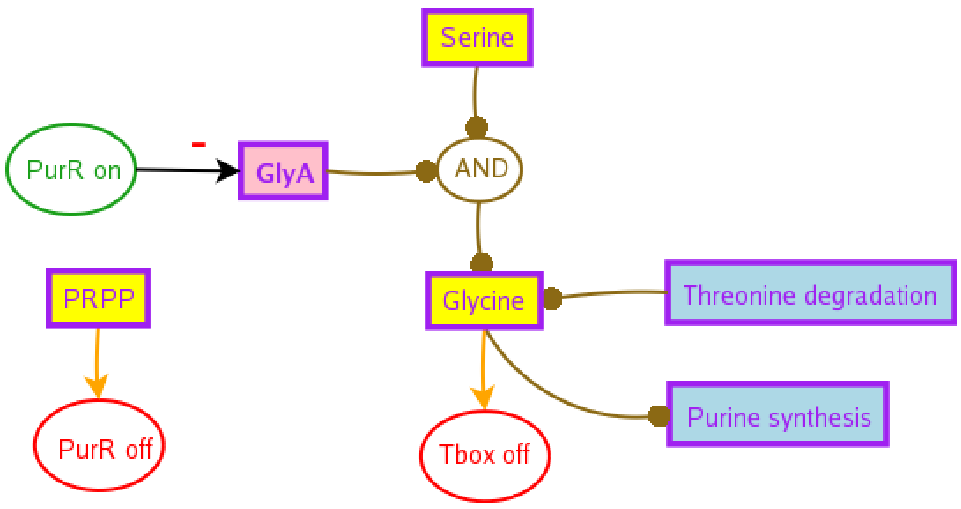

Figure 14: Glycine regulation

3.3) Isoleucine, valine and leucine

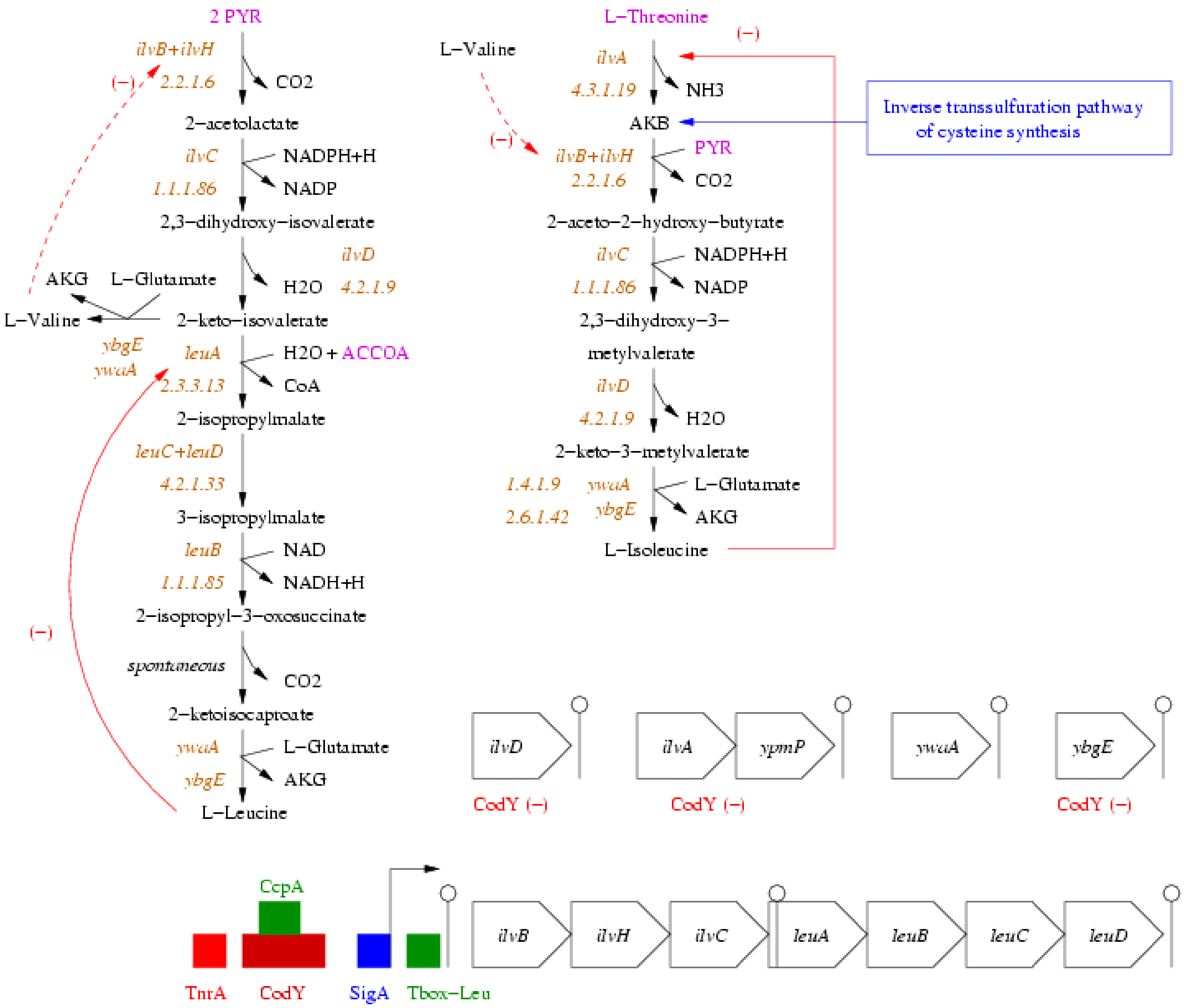

Figure 15: Isoleucine, valine and leucine synthesis

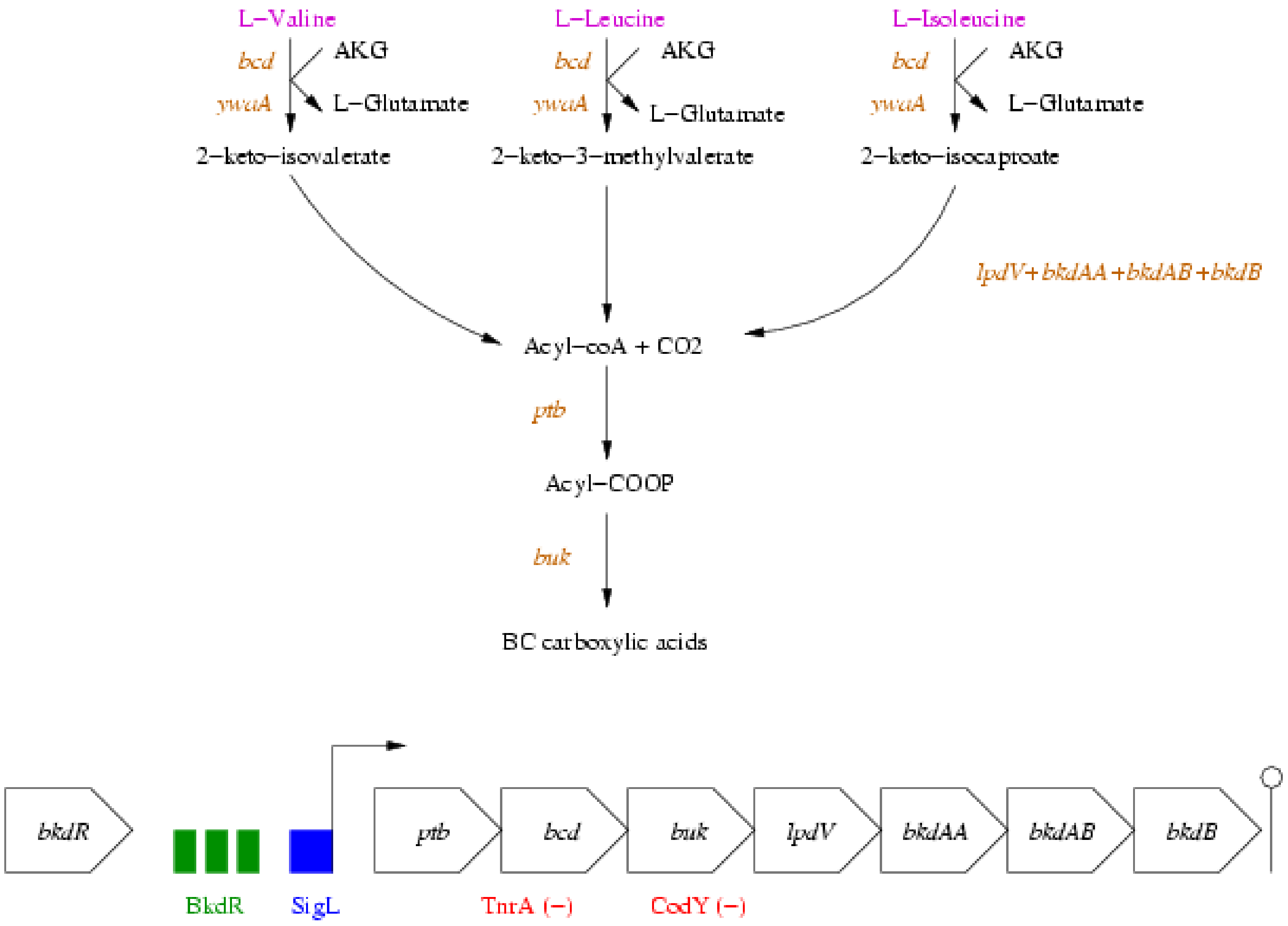

Figure 16: Isoleucine, valine and leucine degradation

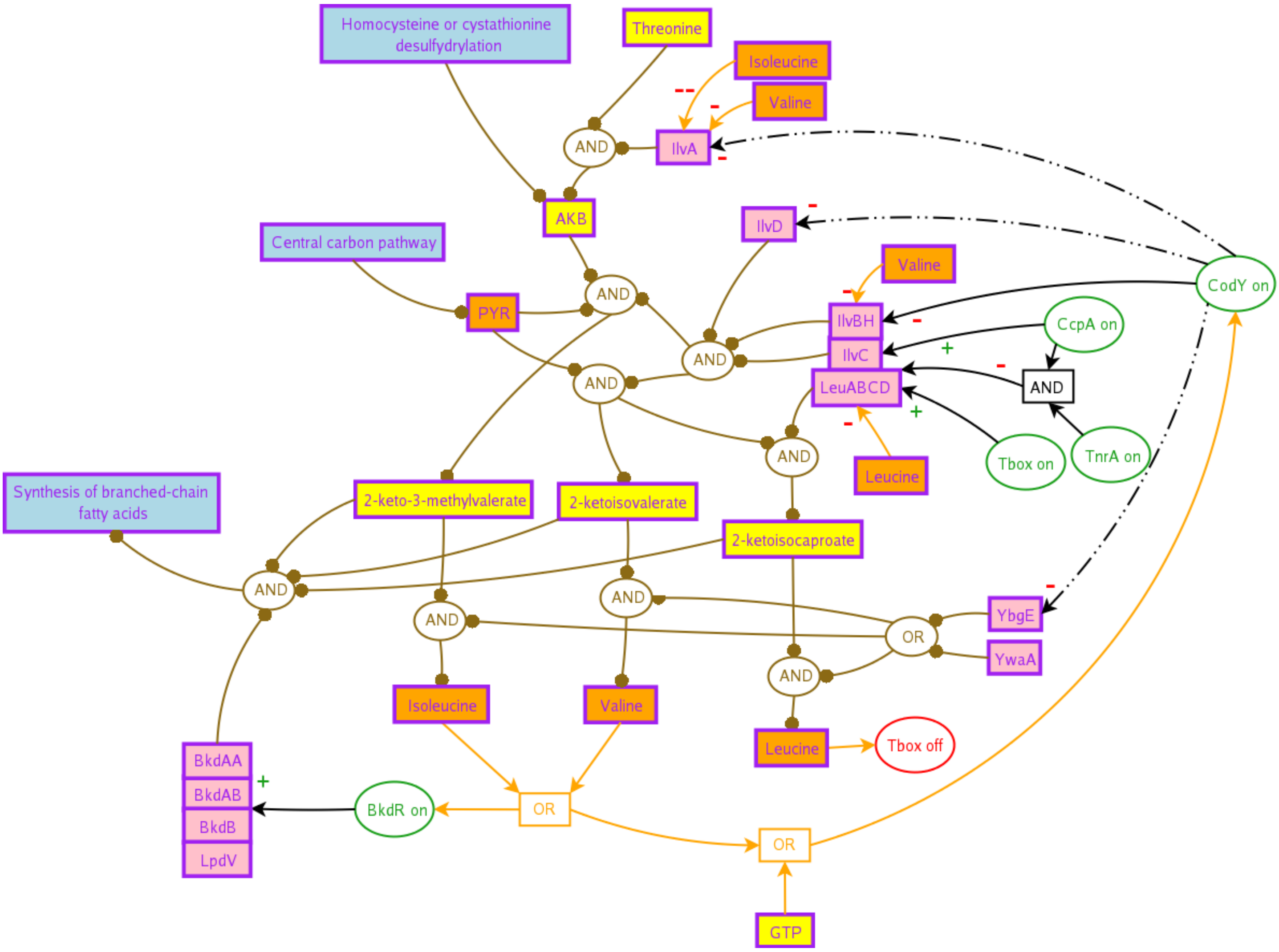

Figure 17: Regulation of branched-chain amino acids metabolism

3.4) Methionine and cysteine

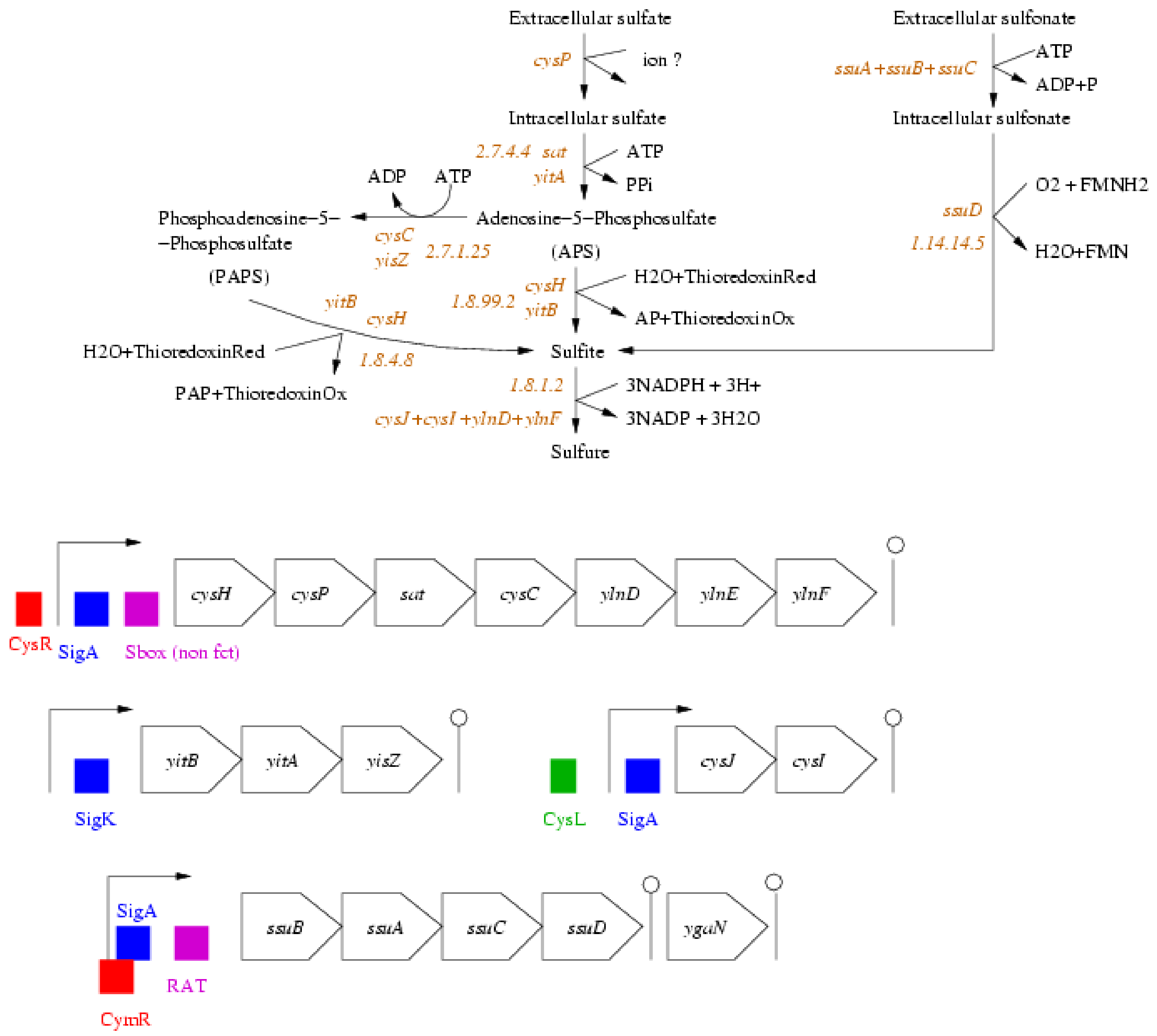

Figure 18: Sulfate assimilation

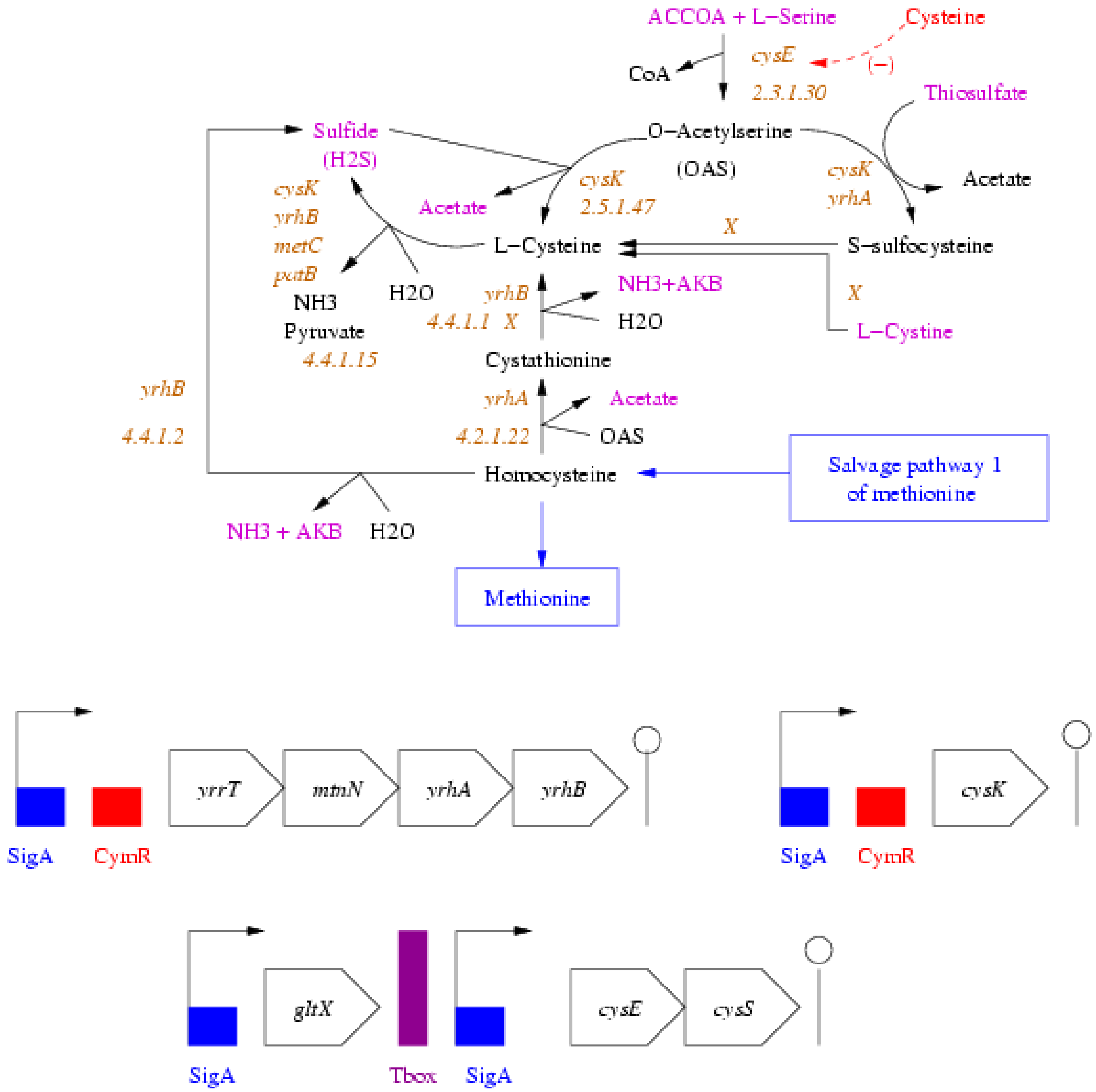

Figure 19: Cysteine metabolism

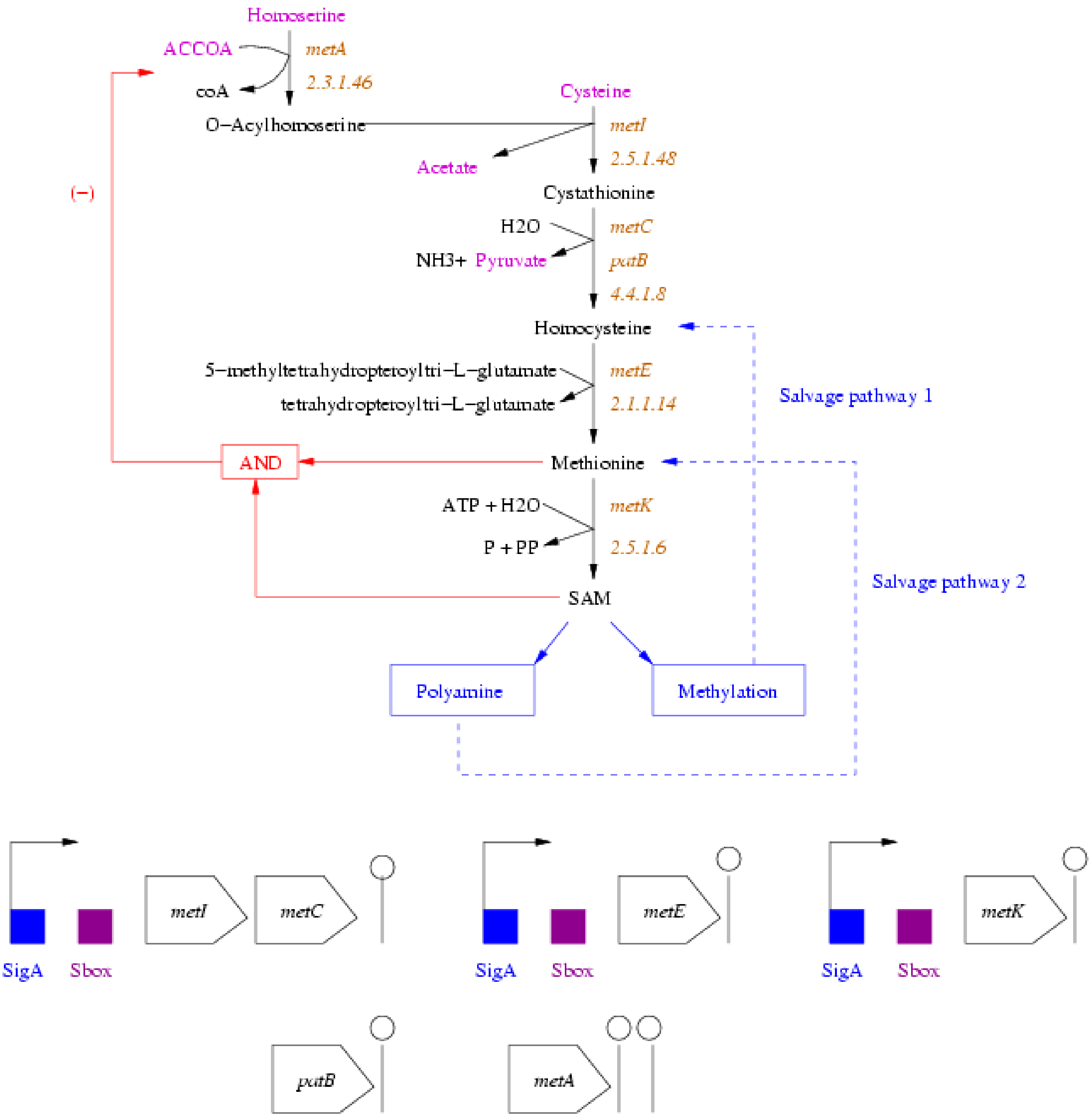

Figure 20: Methionine metabolism

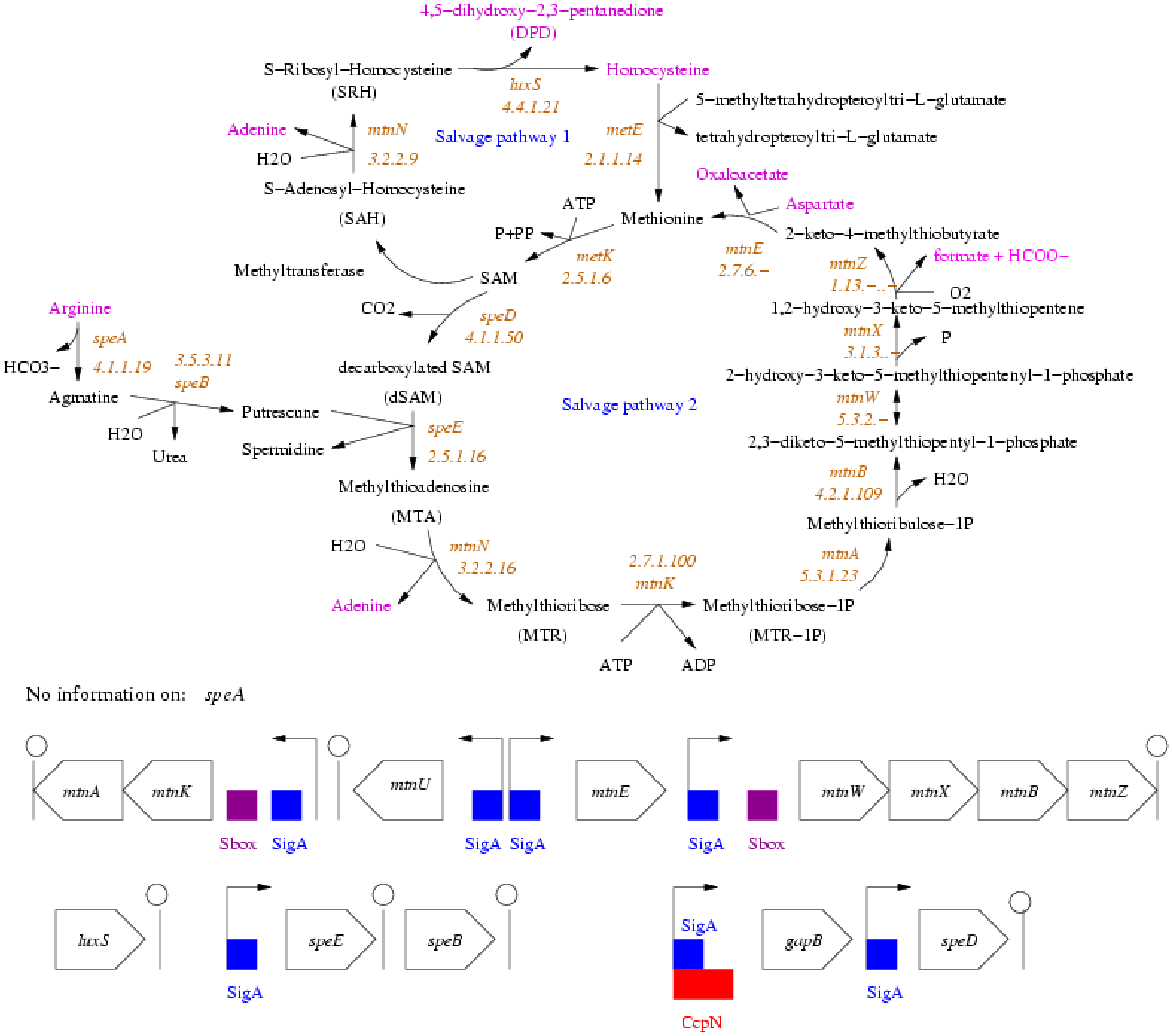

Figure 21: Methionine salvage pathways

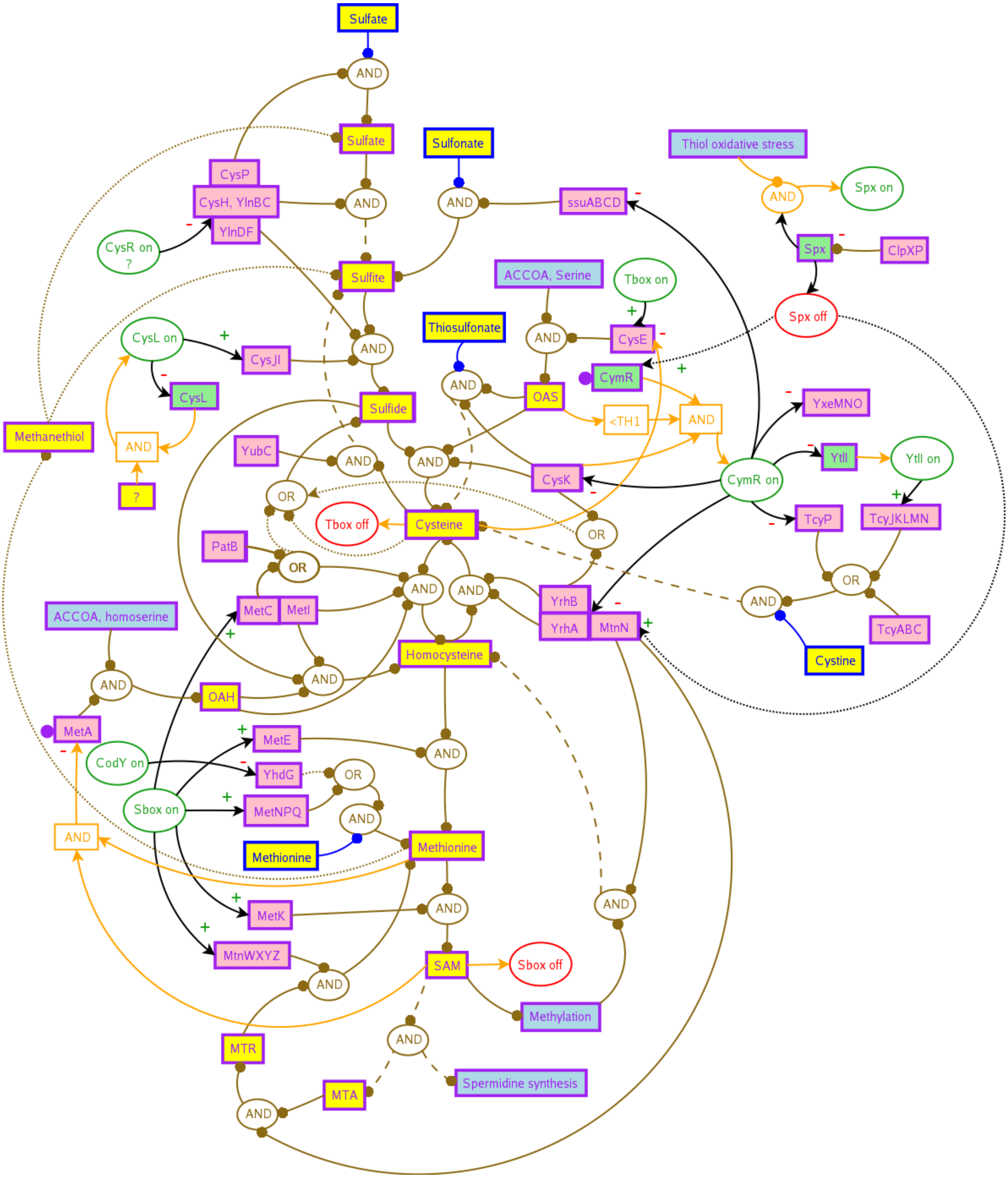

Figure 22: Cysteine and methionine regulation

3.5) Proline

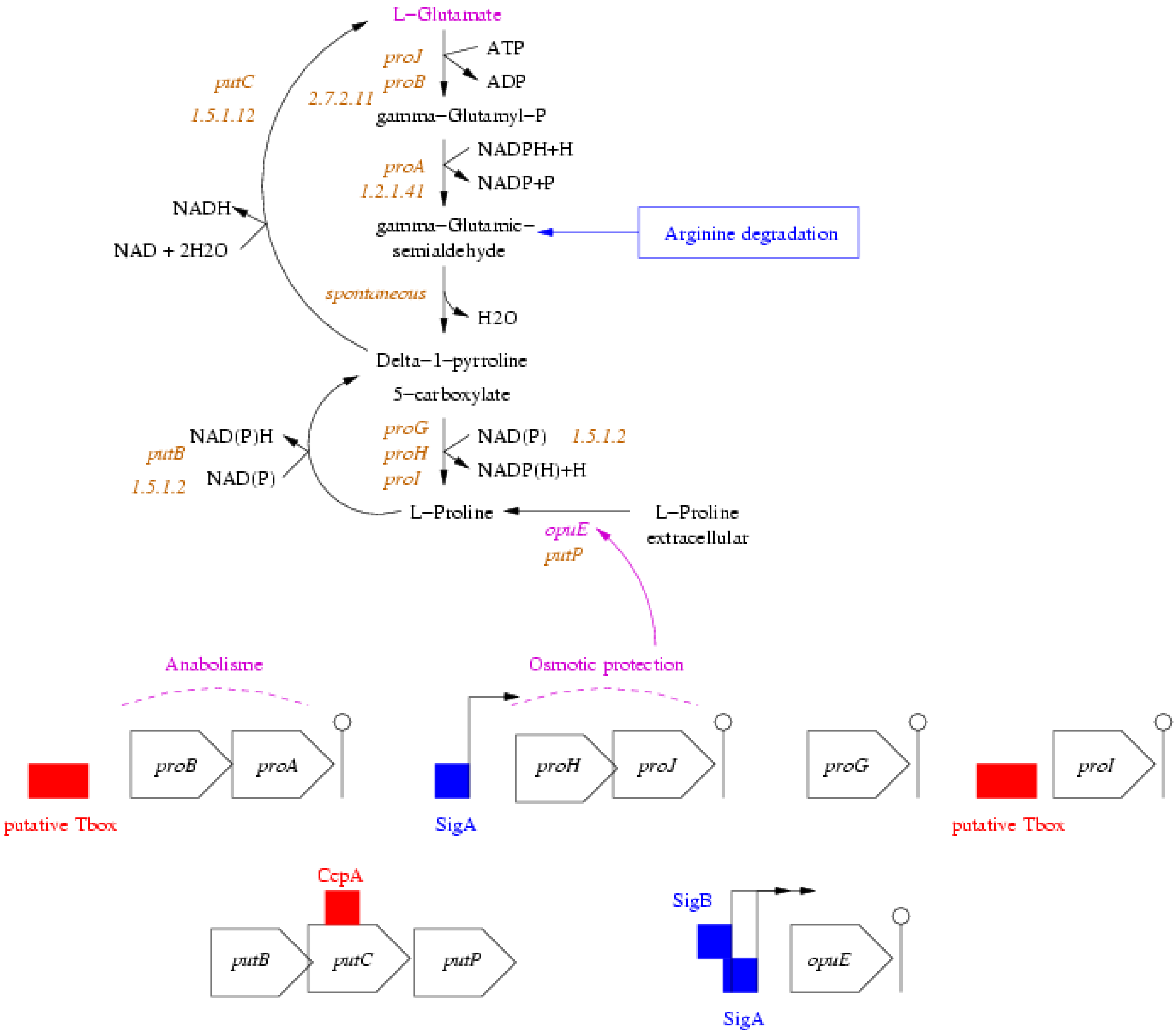

Figure 23: Proline metabolism

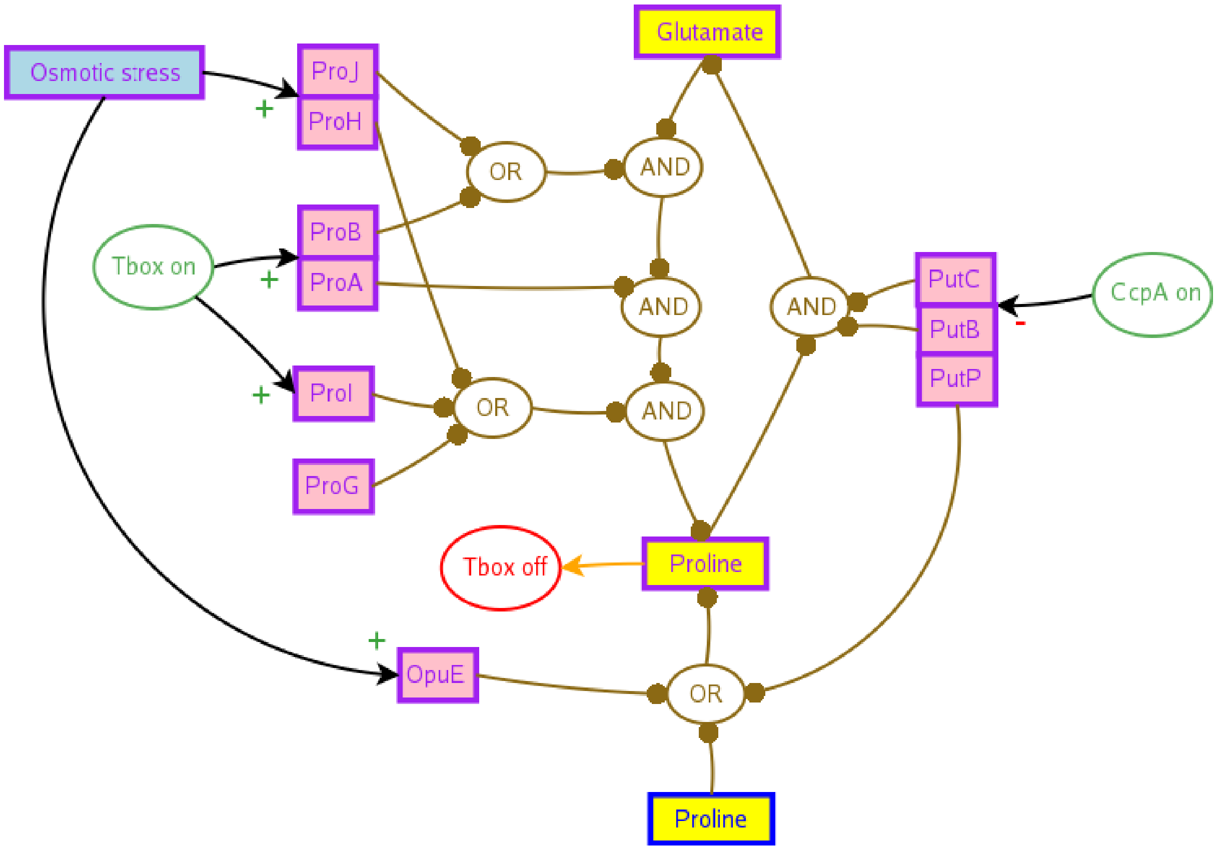

Figure 24: Proline regulation

3.6) Phenylalanine, tyrosine and tryptophan

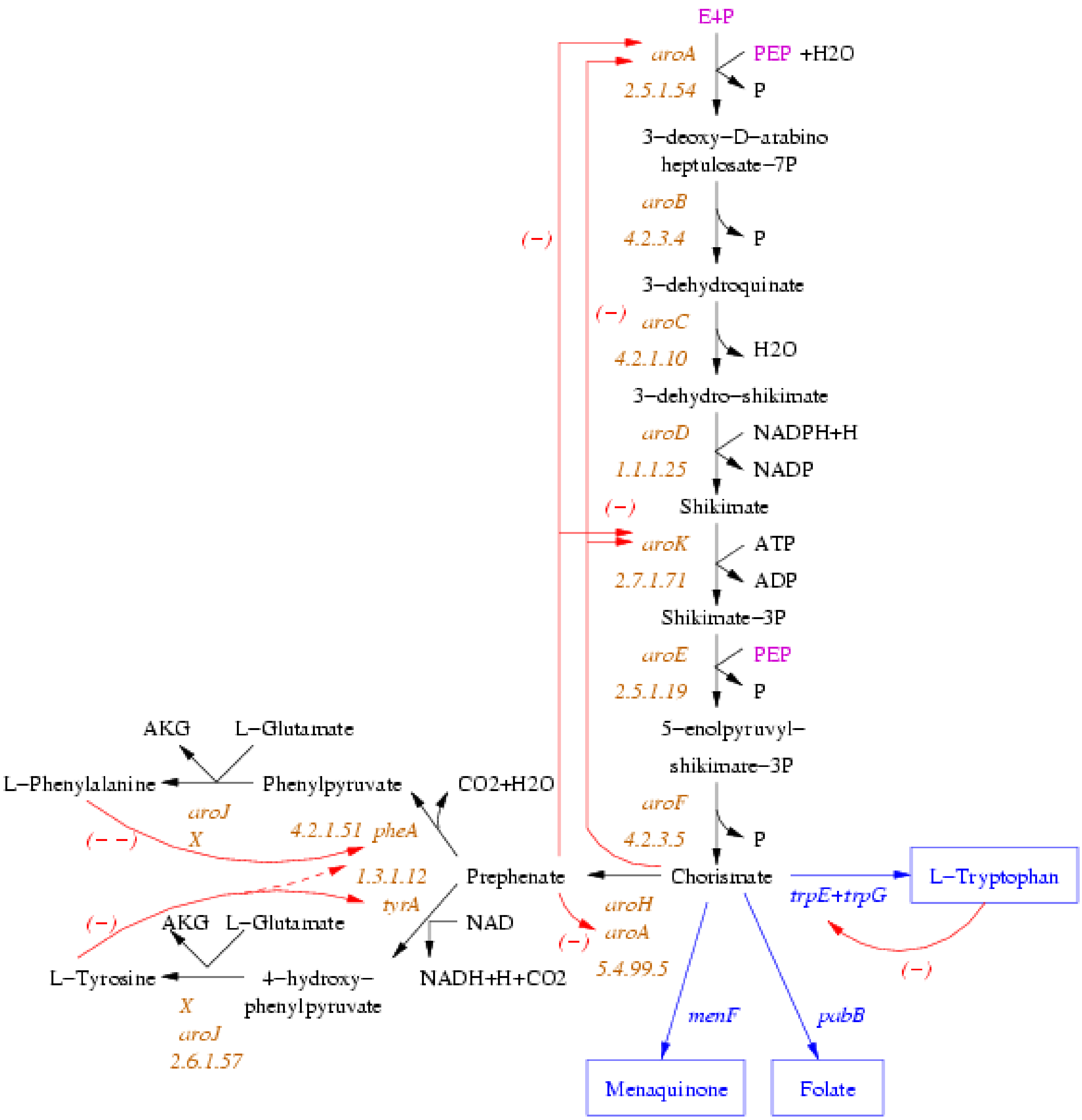

Figure 25: Phenylalanine and tyrosine metabolism

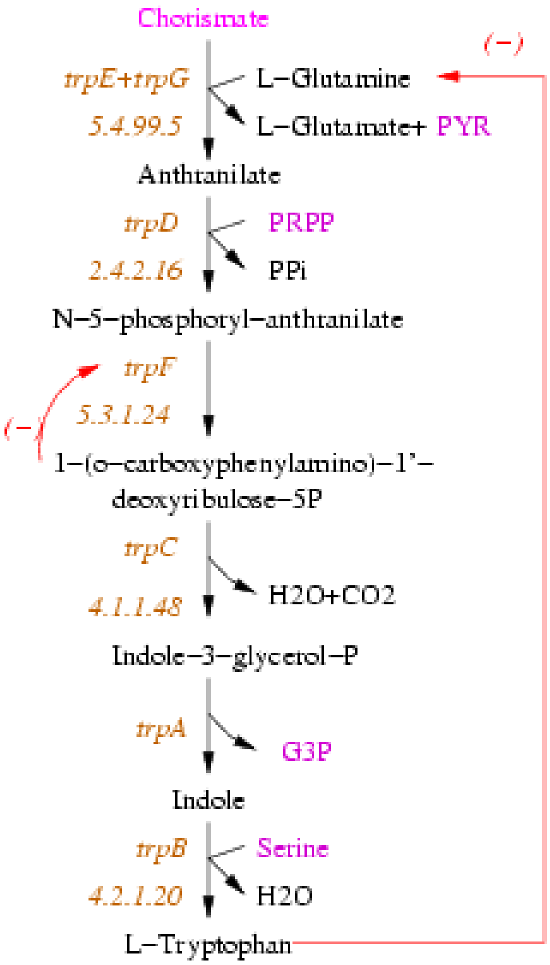

Figure 26: Tryptophan metabolism

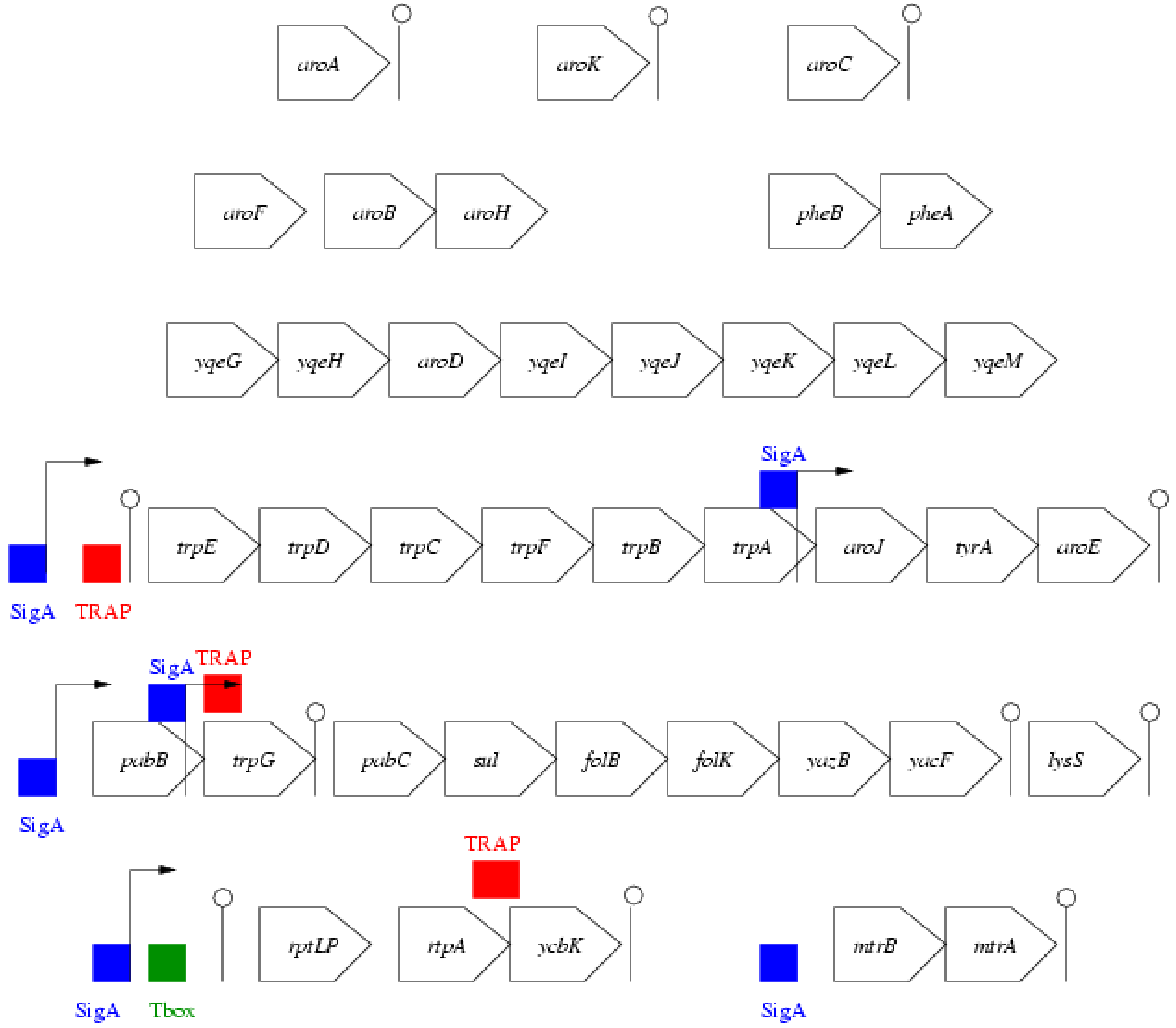

Figure 27: Operons involved in aromatic amino acids synthesis

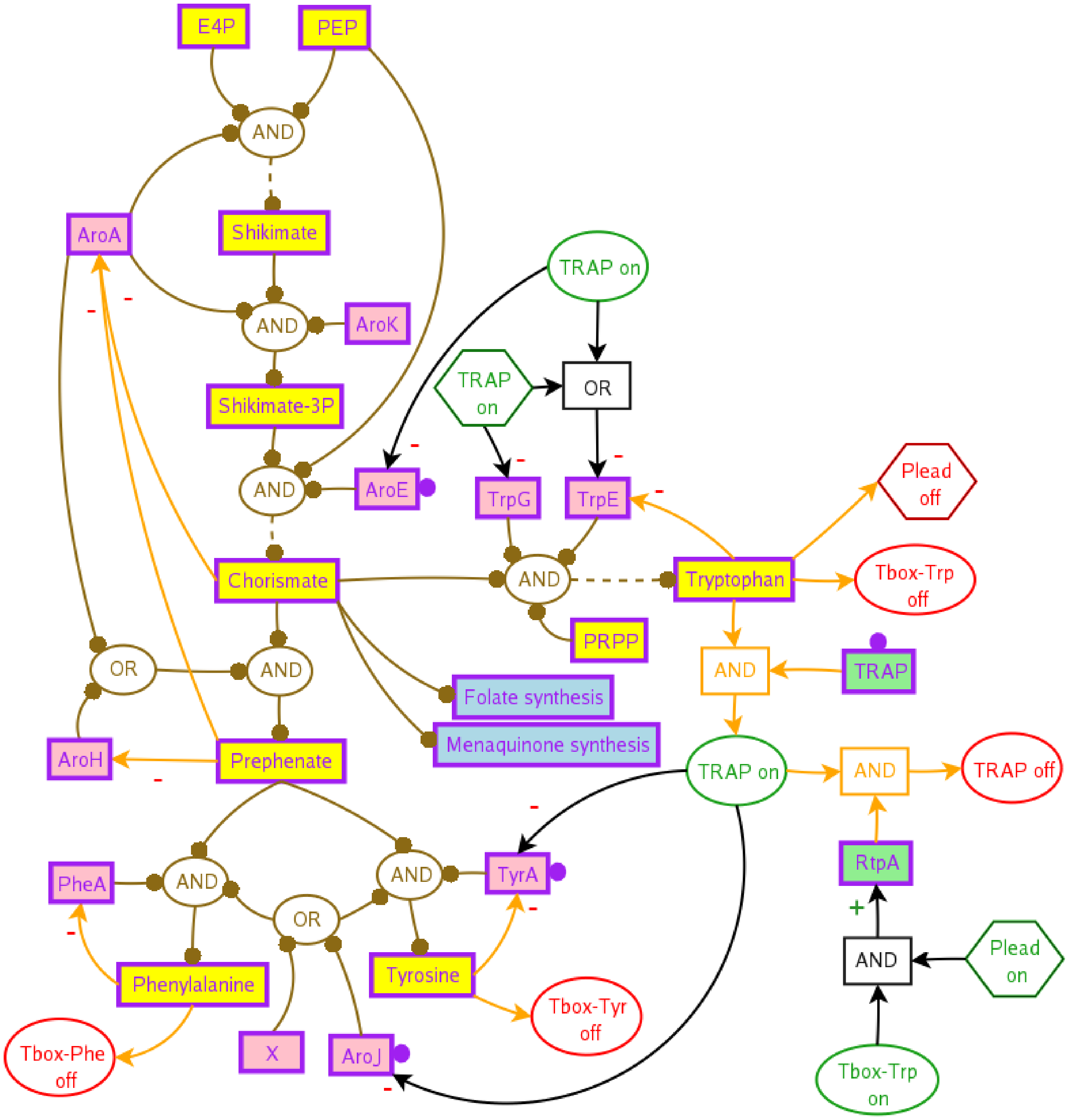

Figure 28: Regulation of aromatic amino acids

3.7) Aspartate and asparagine

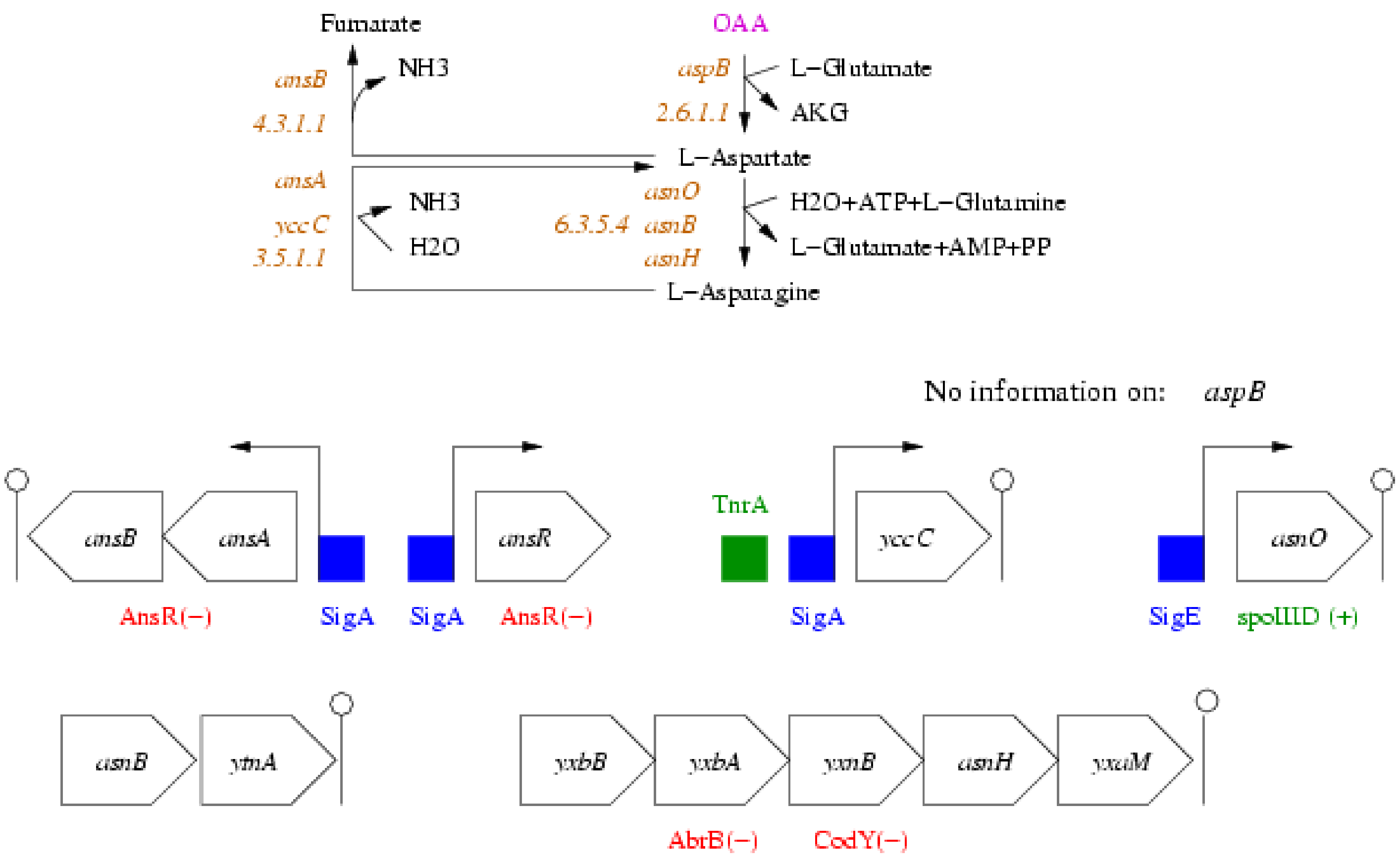

Figure 29: Aspartate and asparagine metabolism

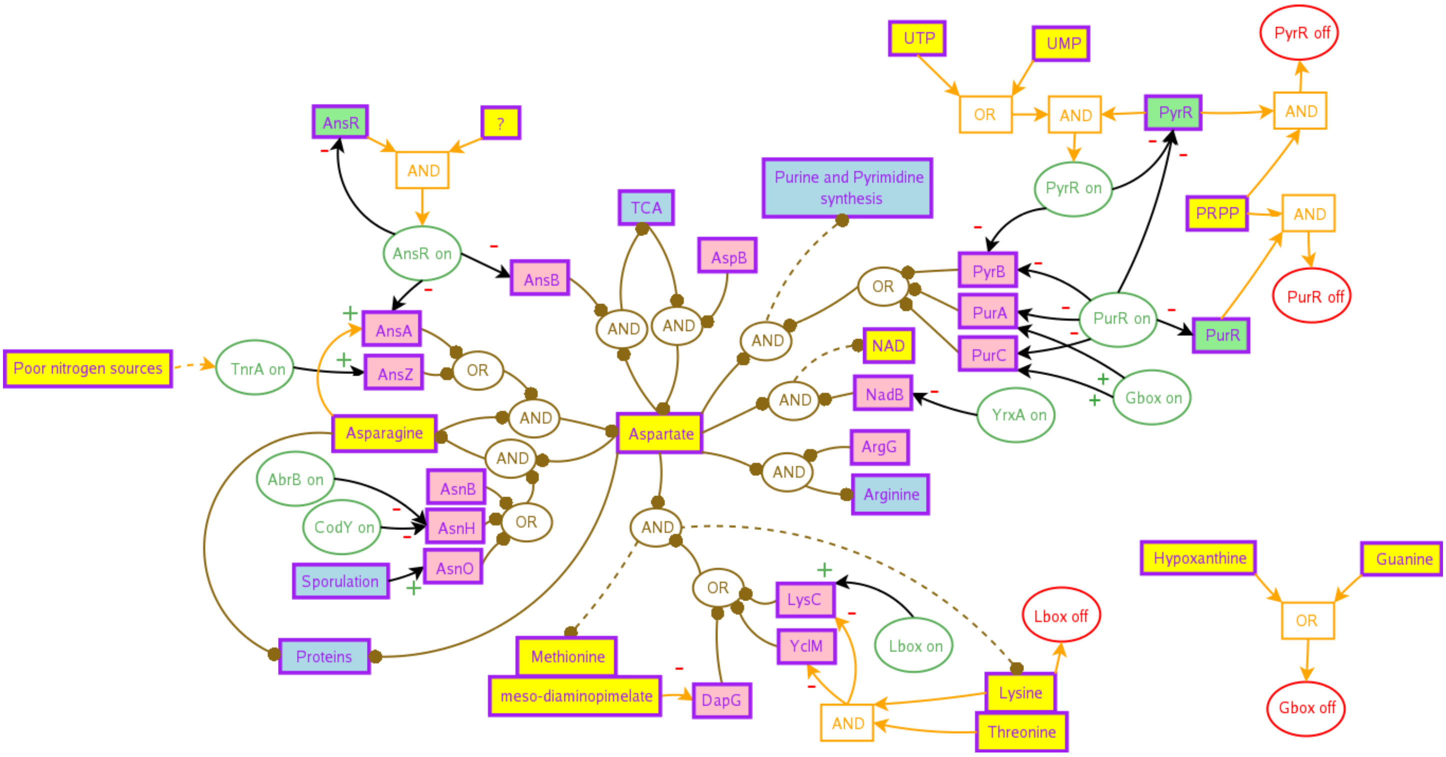

Figure 30: Aspartate and asparagine regulation

3.8) Glutamate and glutamine

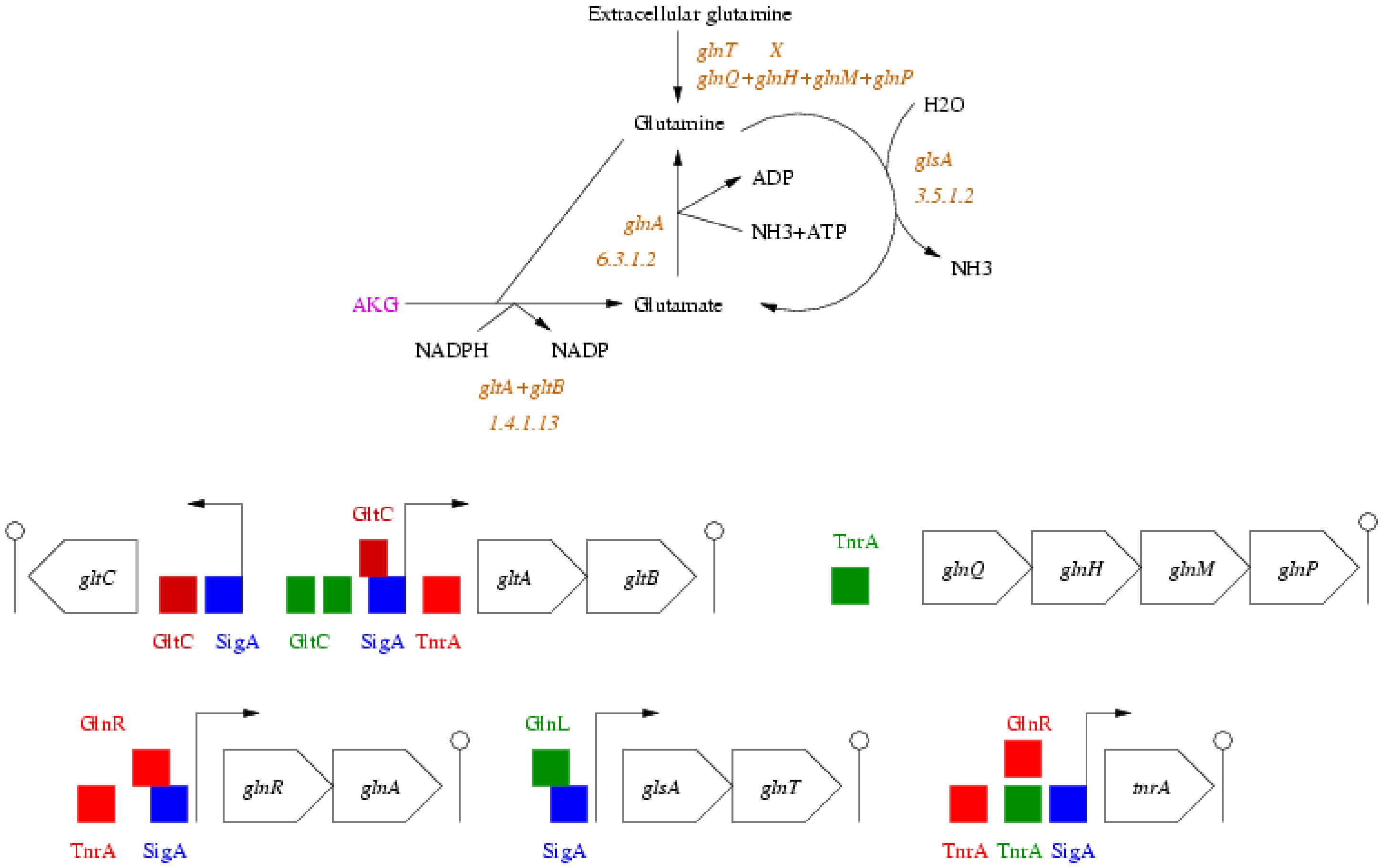

Figure 31: Glutamate and glutamine metabolism

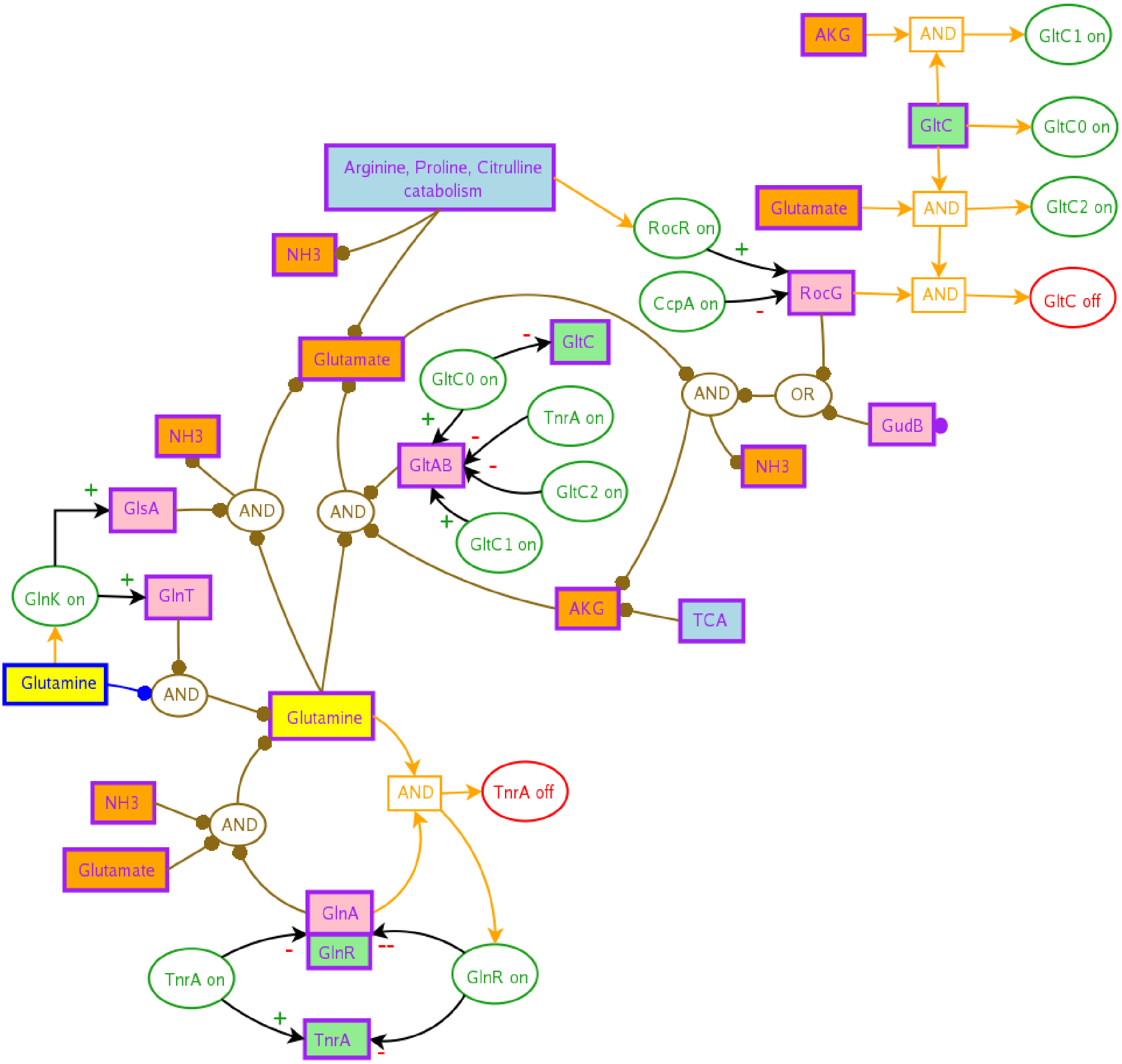

Figure 32: Glutamate and glutamine regulation

3.9) Arginine

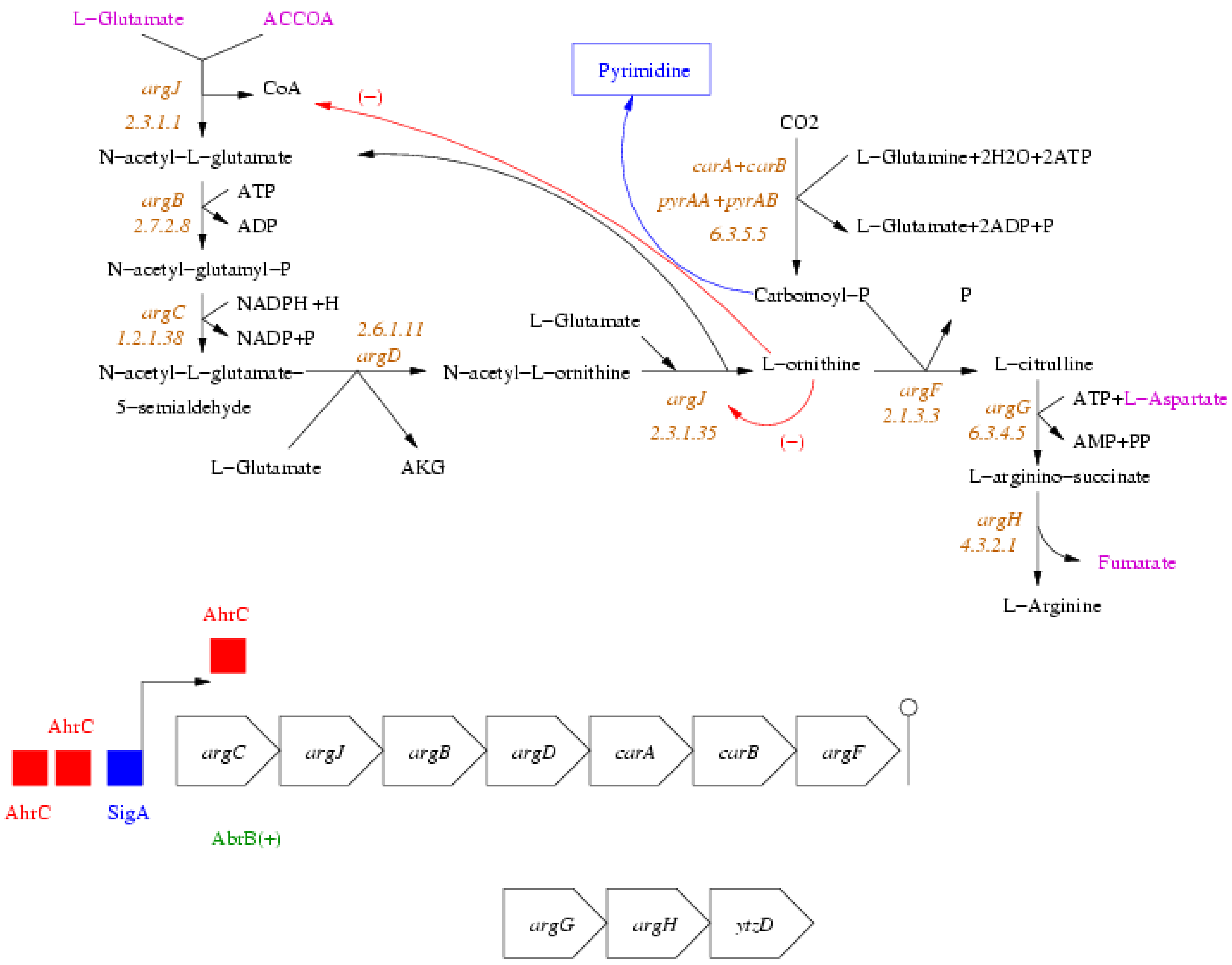

Figure 33: Arginine metabolism

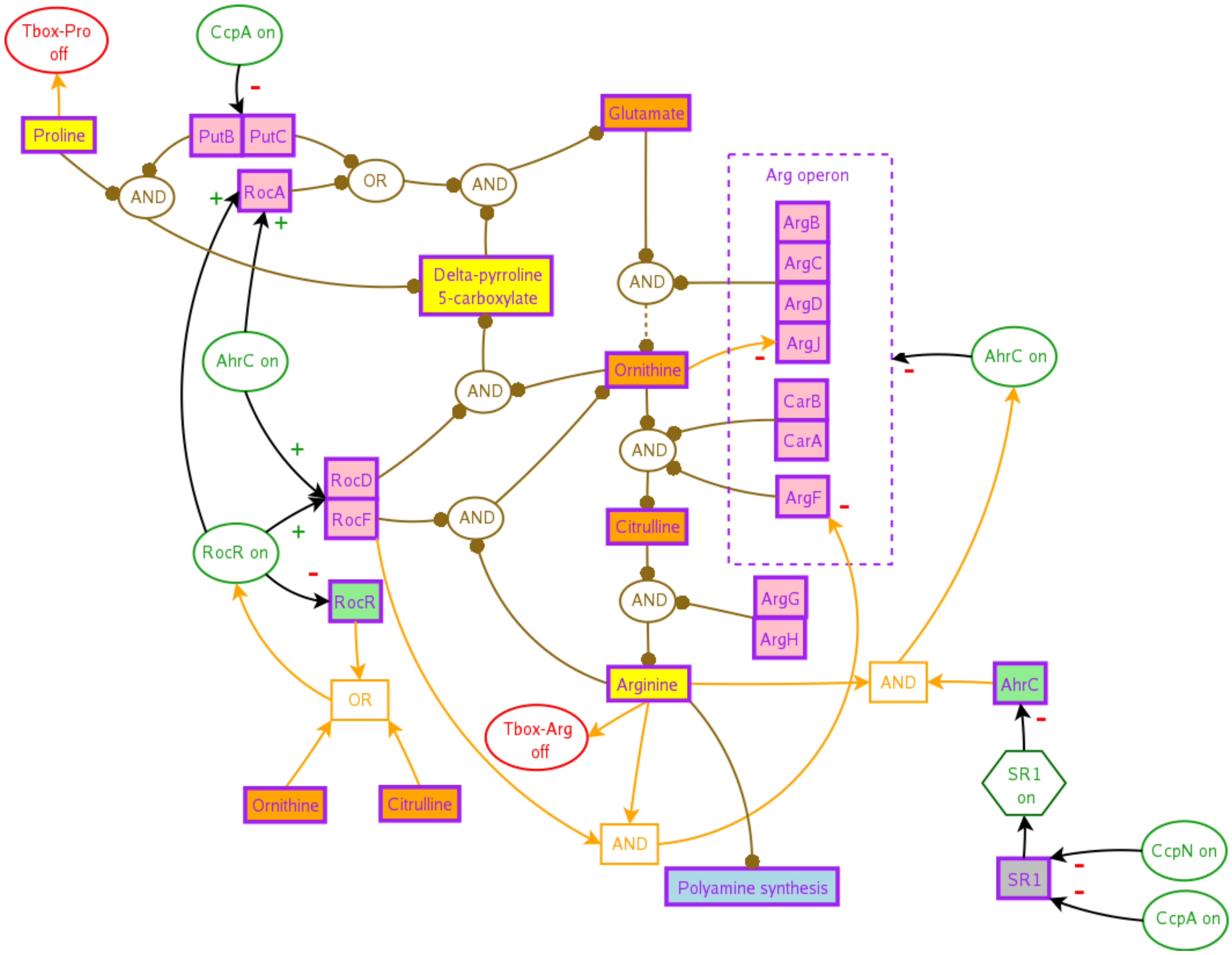

Figure 34: Arginine regulation and link with proline degradation

3.10) Histidine

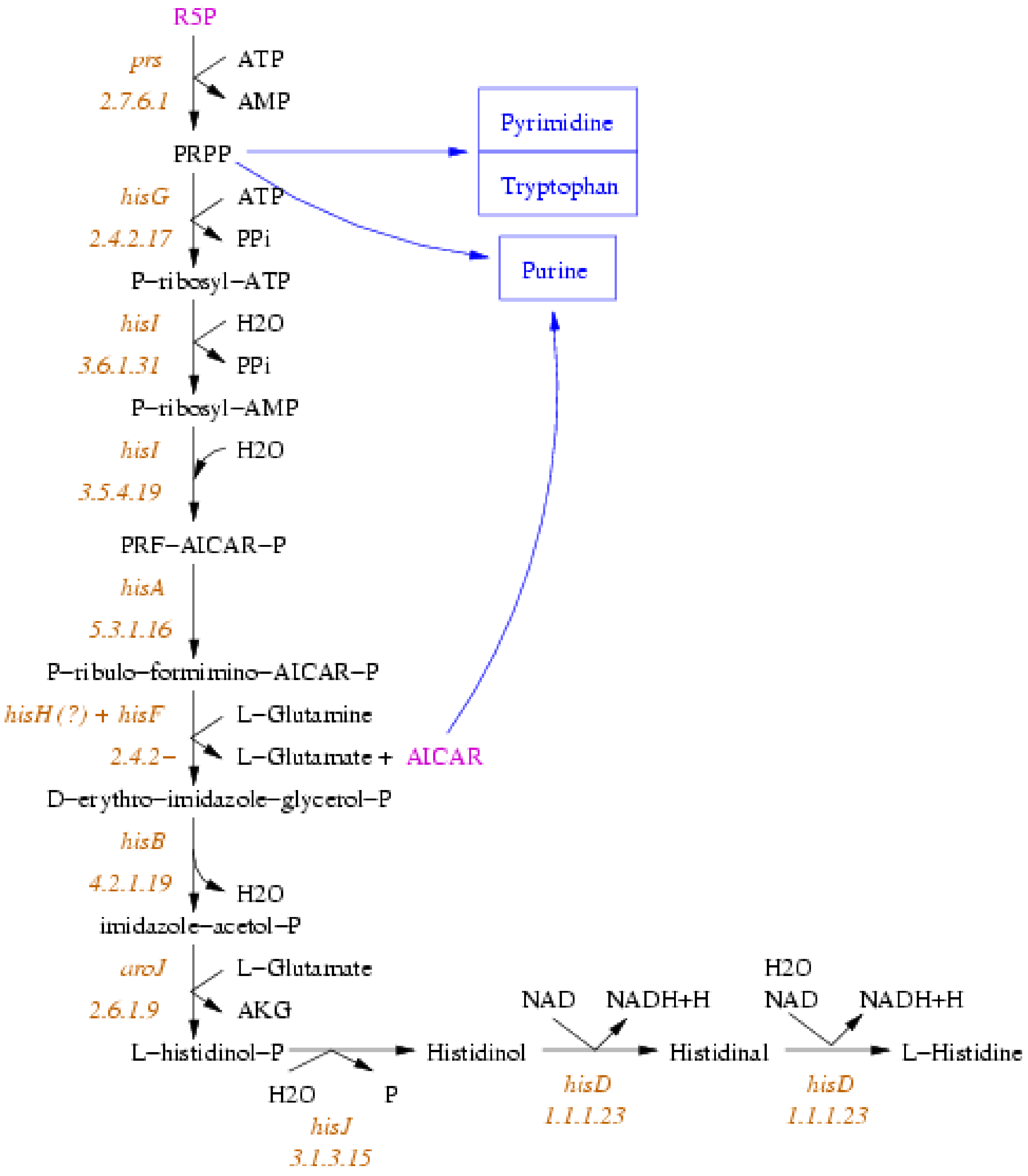

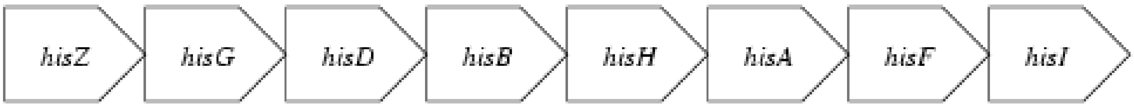

No information on: *hisJ*

See purine metabolism for prs regulation

See aromatic amino acids synthesis for aroJ regulation

Figure 35: Histidine synthesis

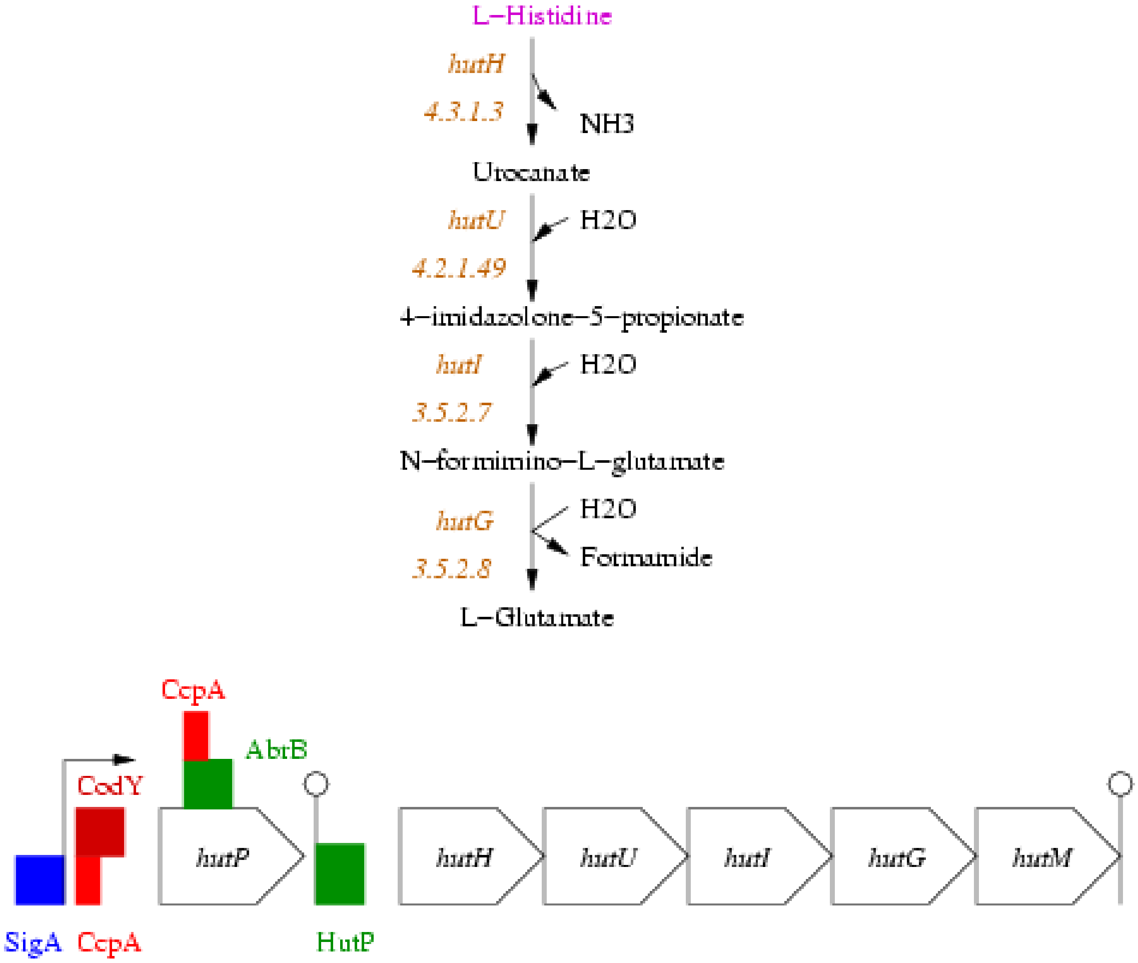

Figure 36: Histidine degradation

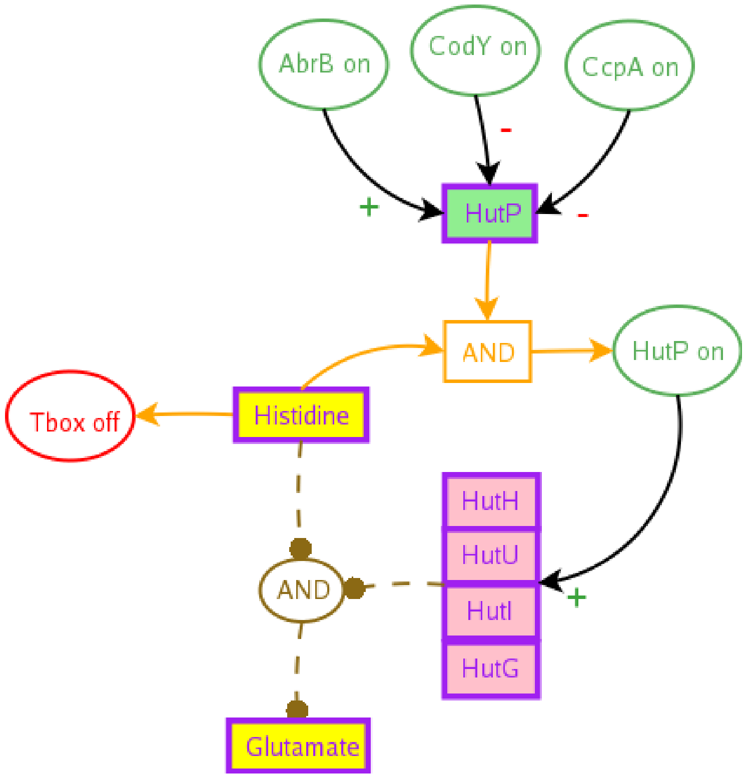

Figure 37: Histidine regulation

3.11) Lysine and threonine

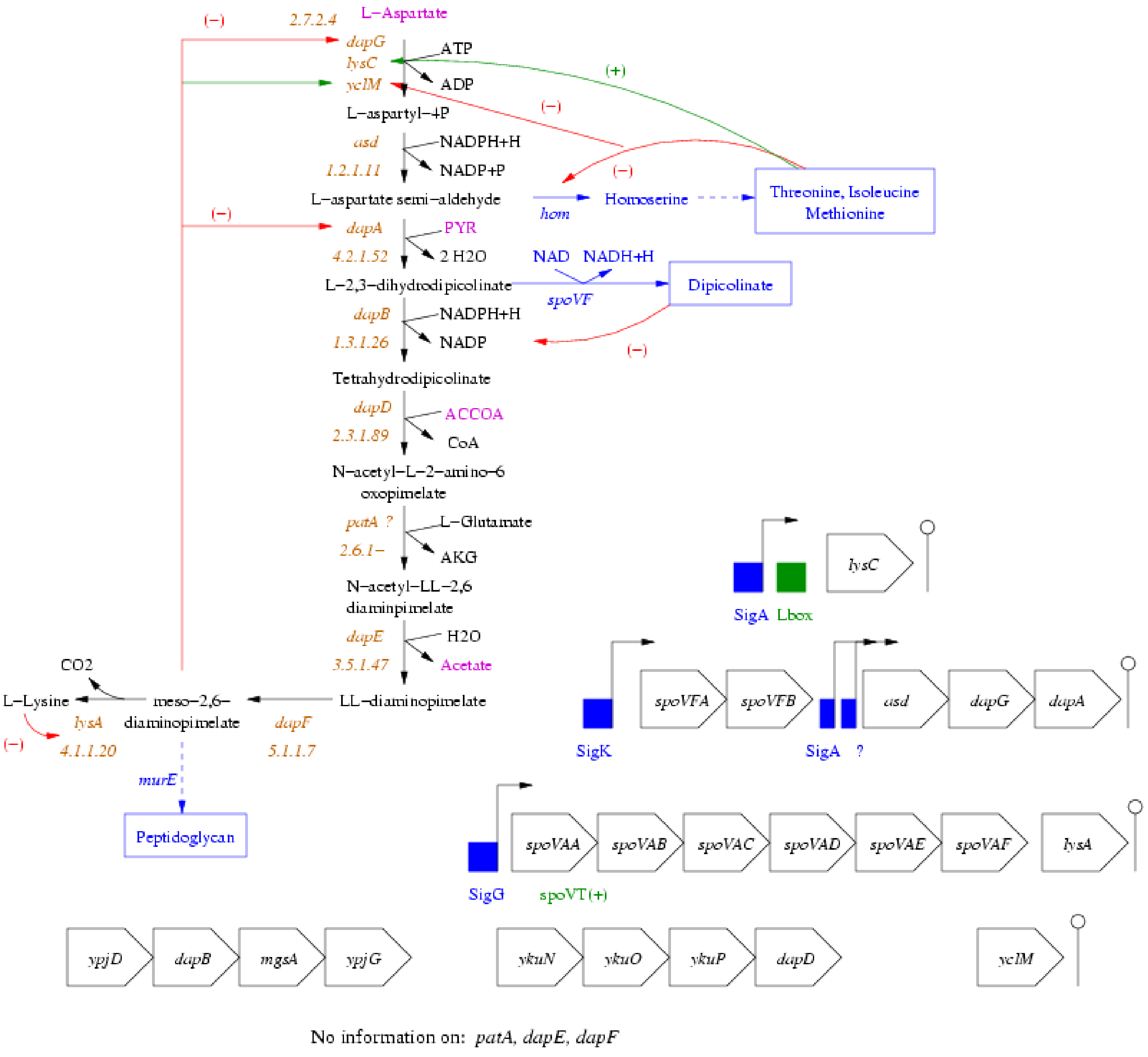

Figure 38: Lysine metabolism

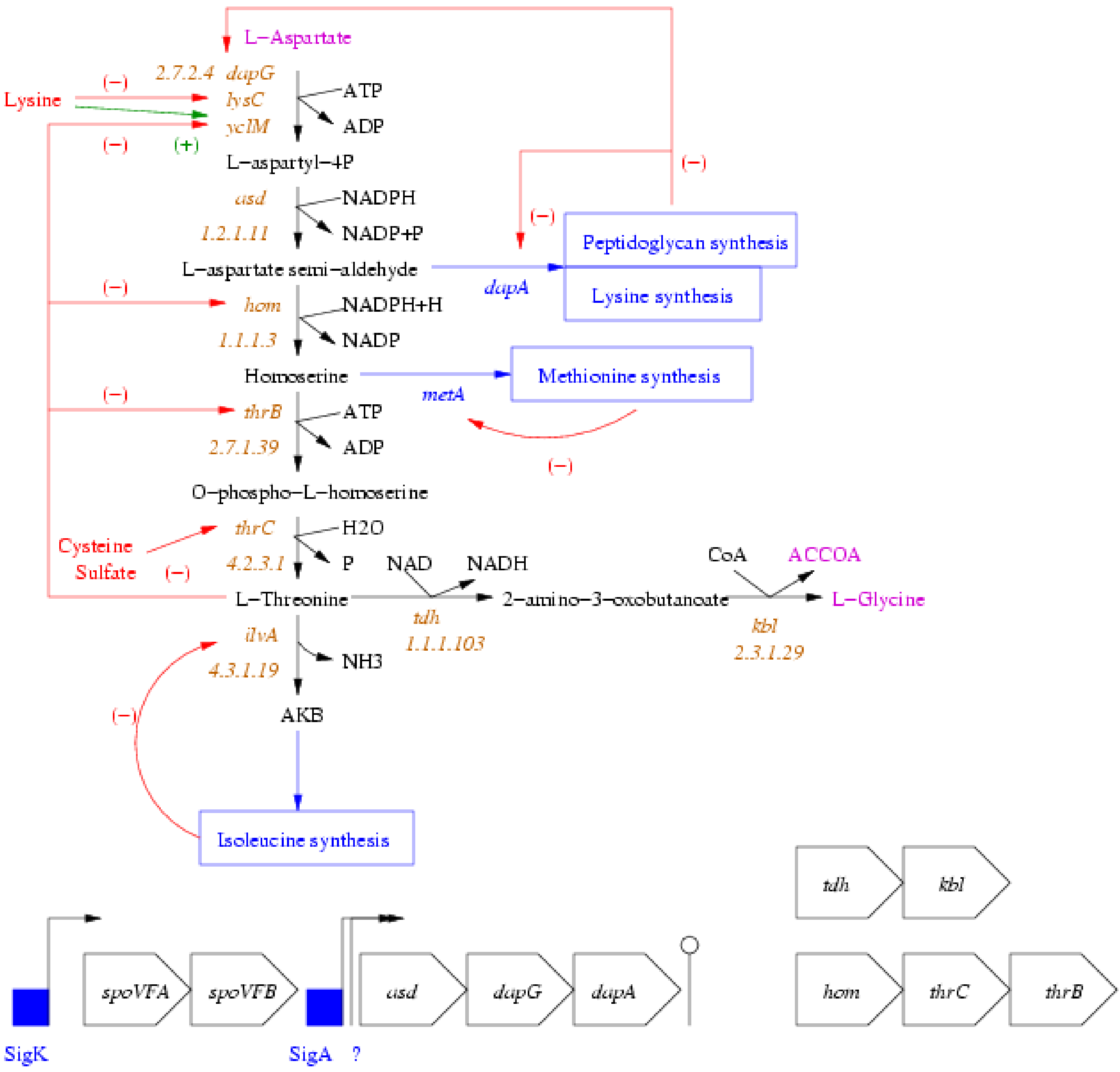

See branched–chain amino acids synthesis for ilvA regulation

Figure 39: Threonine metabolism

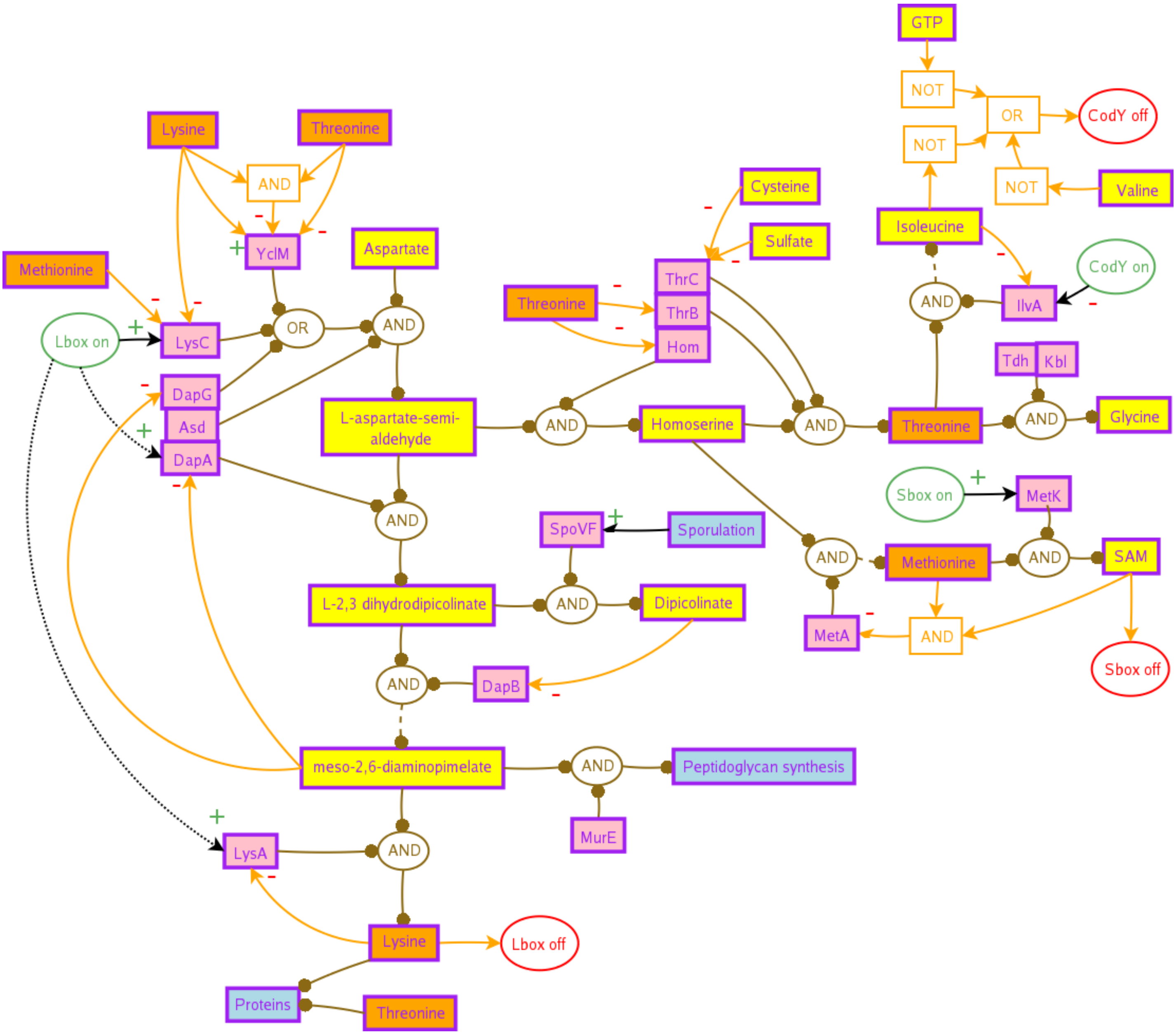

Figure 40: Lysine and threonine metabolism

3.12) Serine

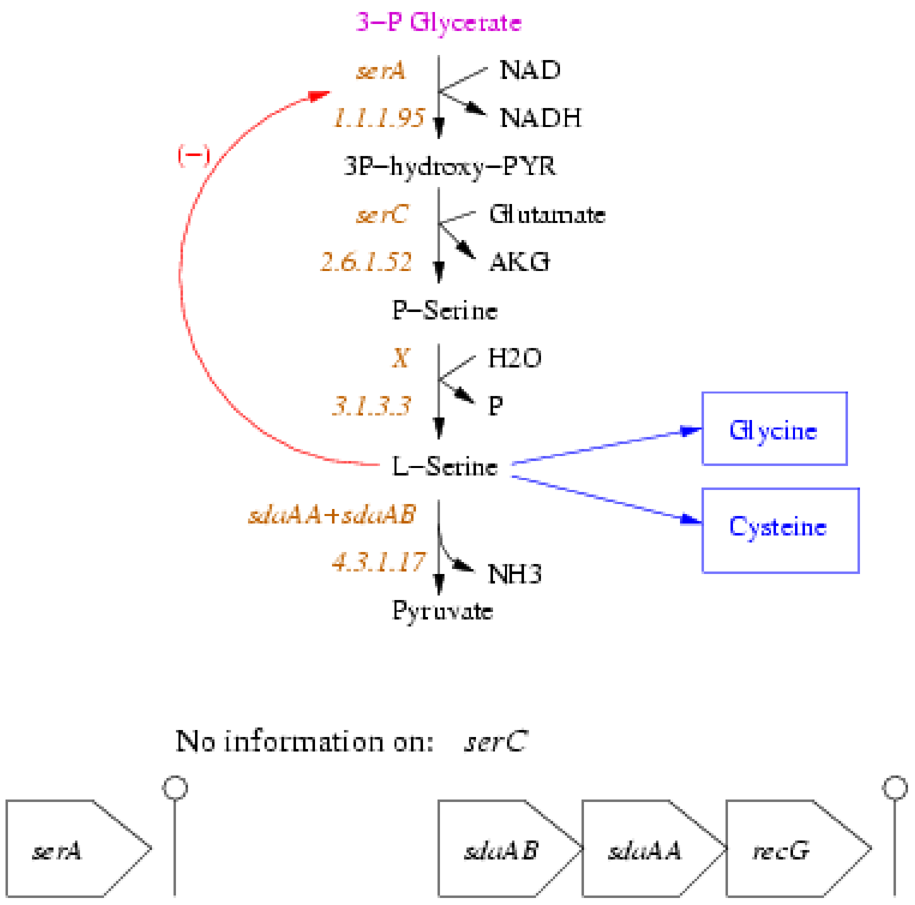

Figure 41: Serine metabolism

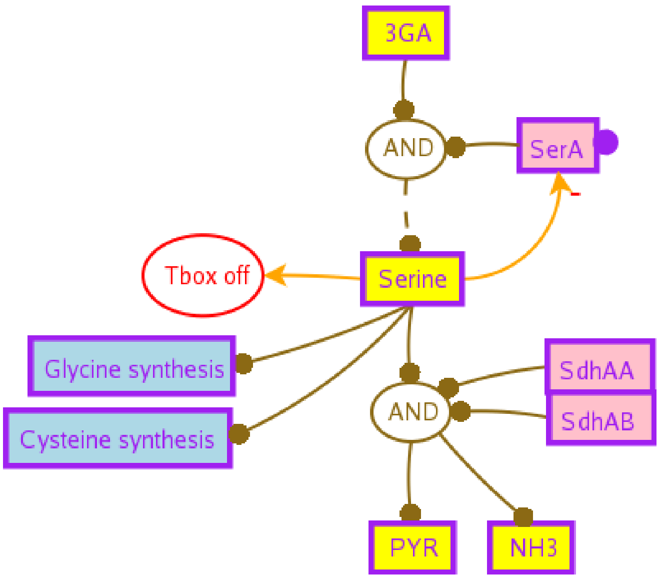

Figure 42: Serine regulation

4. Nucleotides metabolism

4.1) Synthesis of nucleotides

Purines

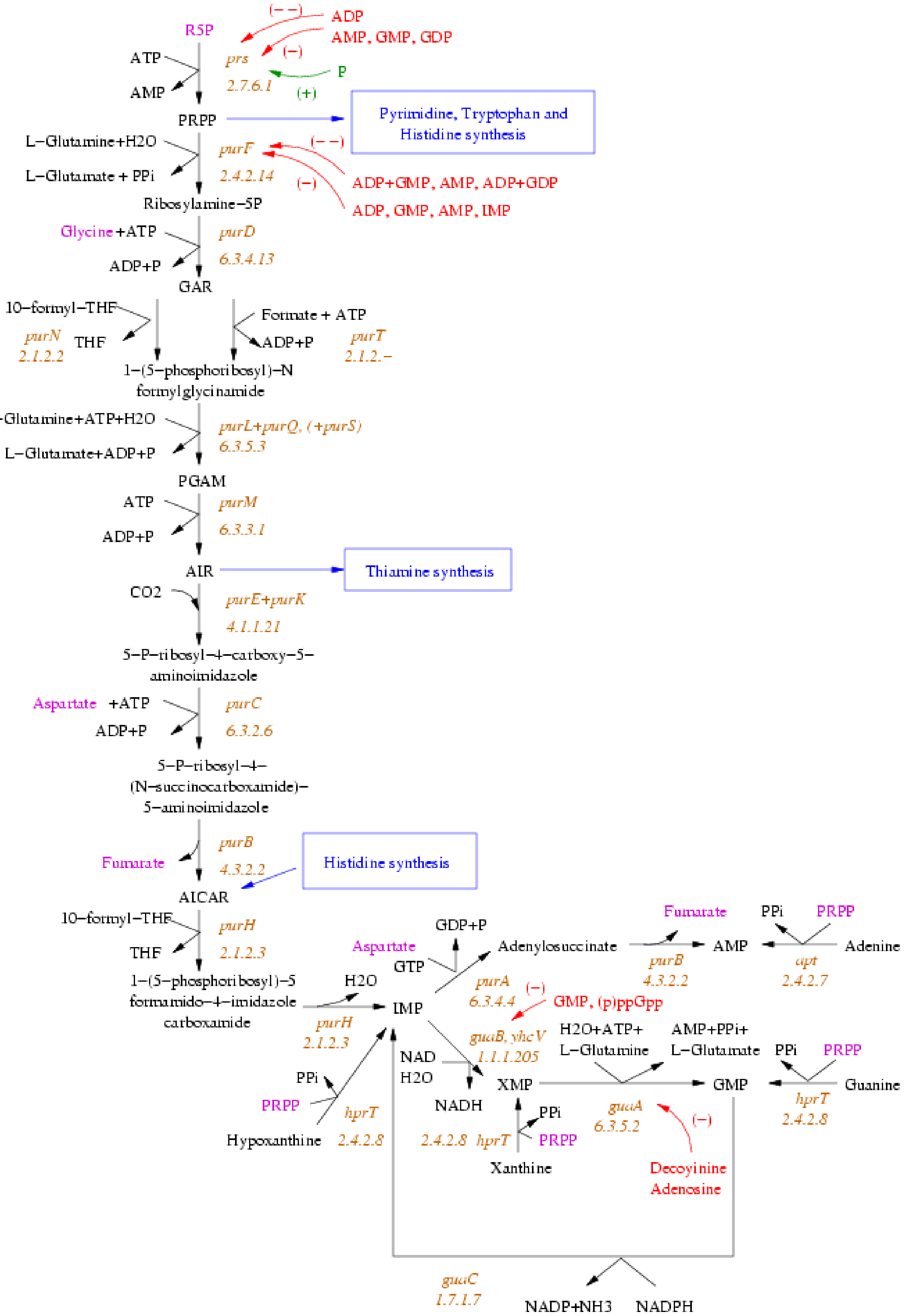

Figure 43: Purines metabolism

Pyrimidines

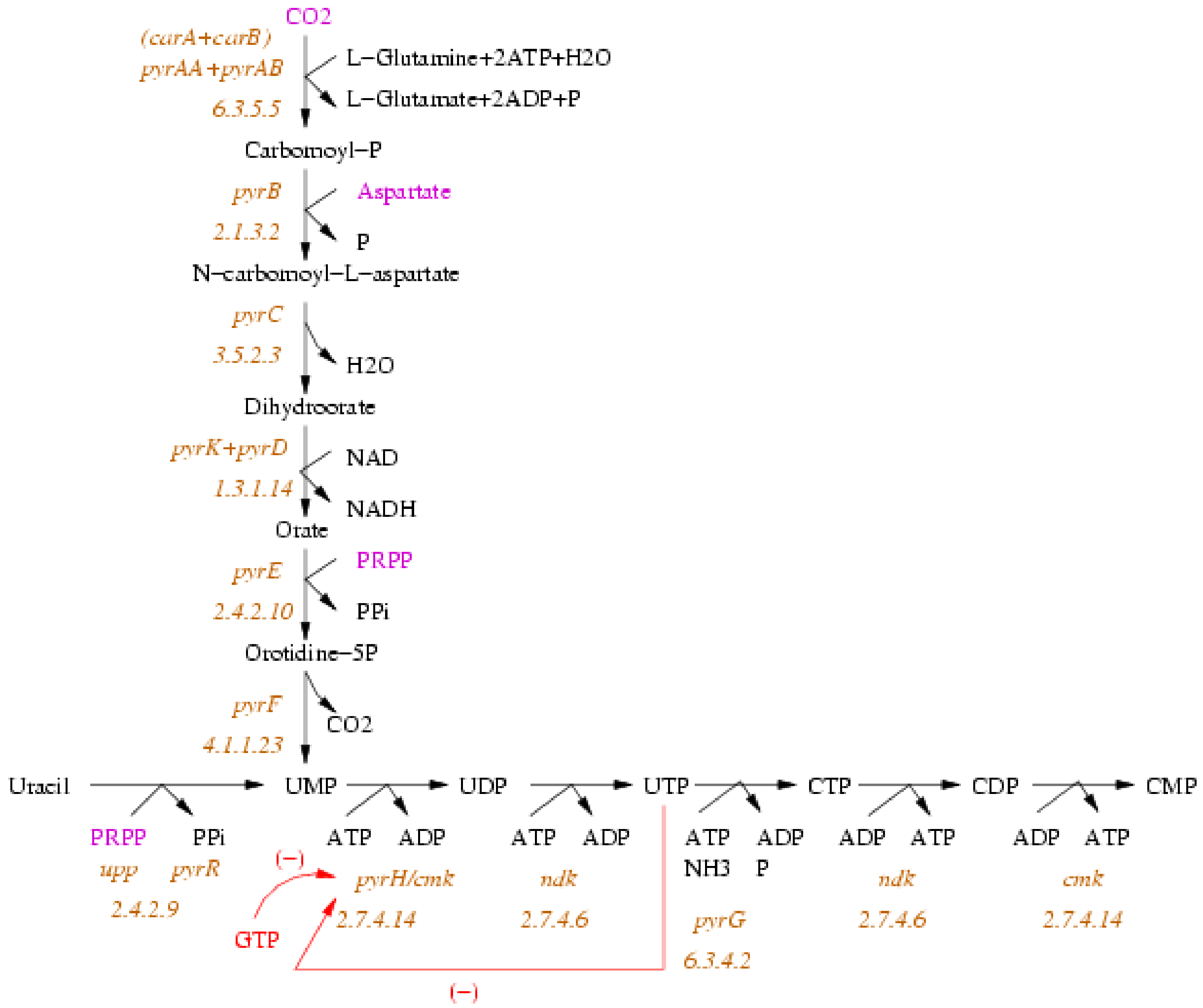

Figure 44: Pyrimidines metabolism

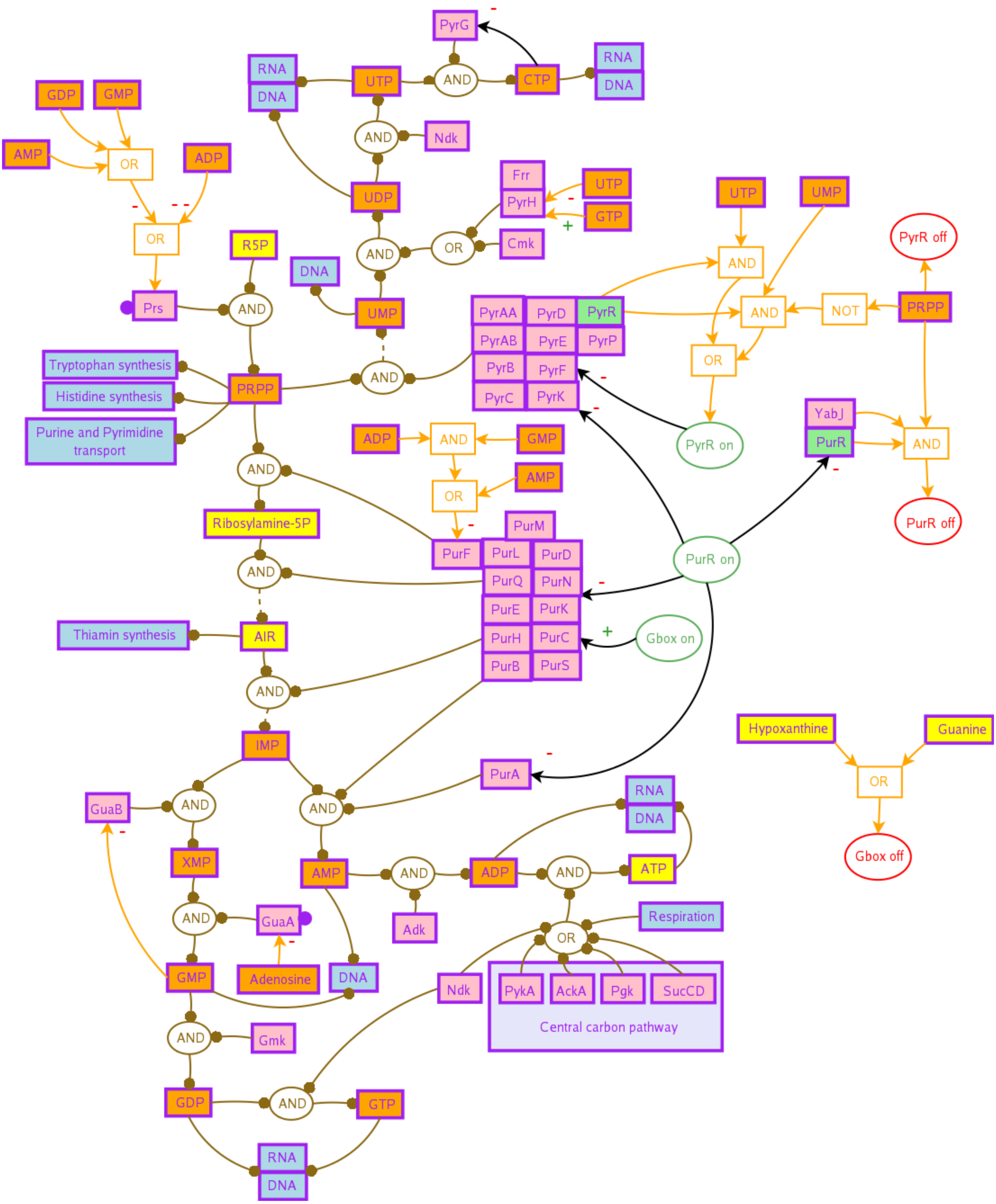

Figure 45: Purines and pyrimidines regulation

4.2) Purines and Pyrimidines salvage pathway

Nucleosides catabolism

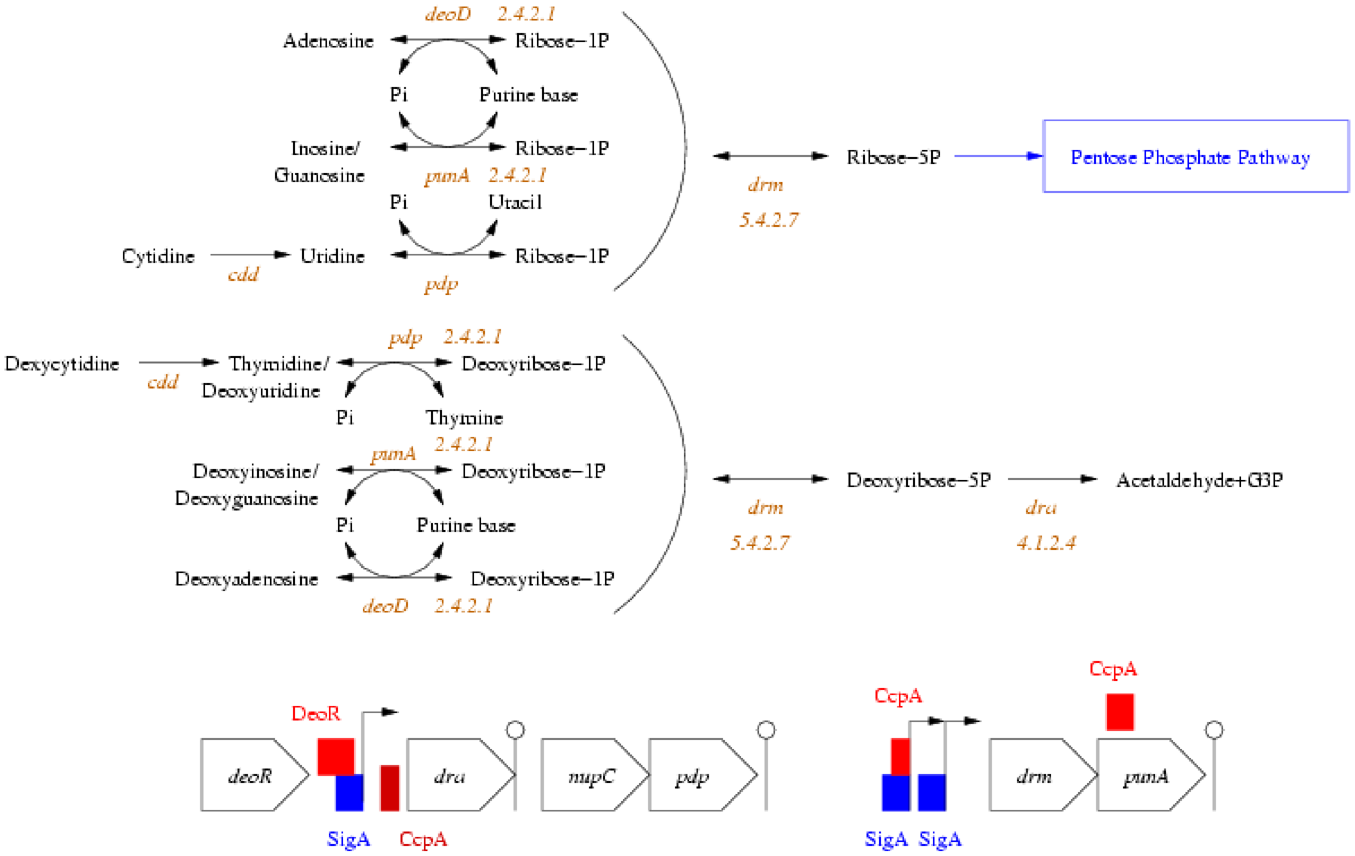

Figure 46: Nucleosides catabolism

Purines salvage pathway

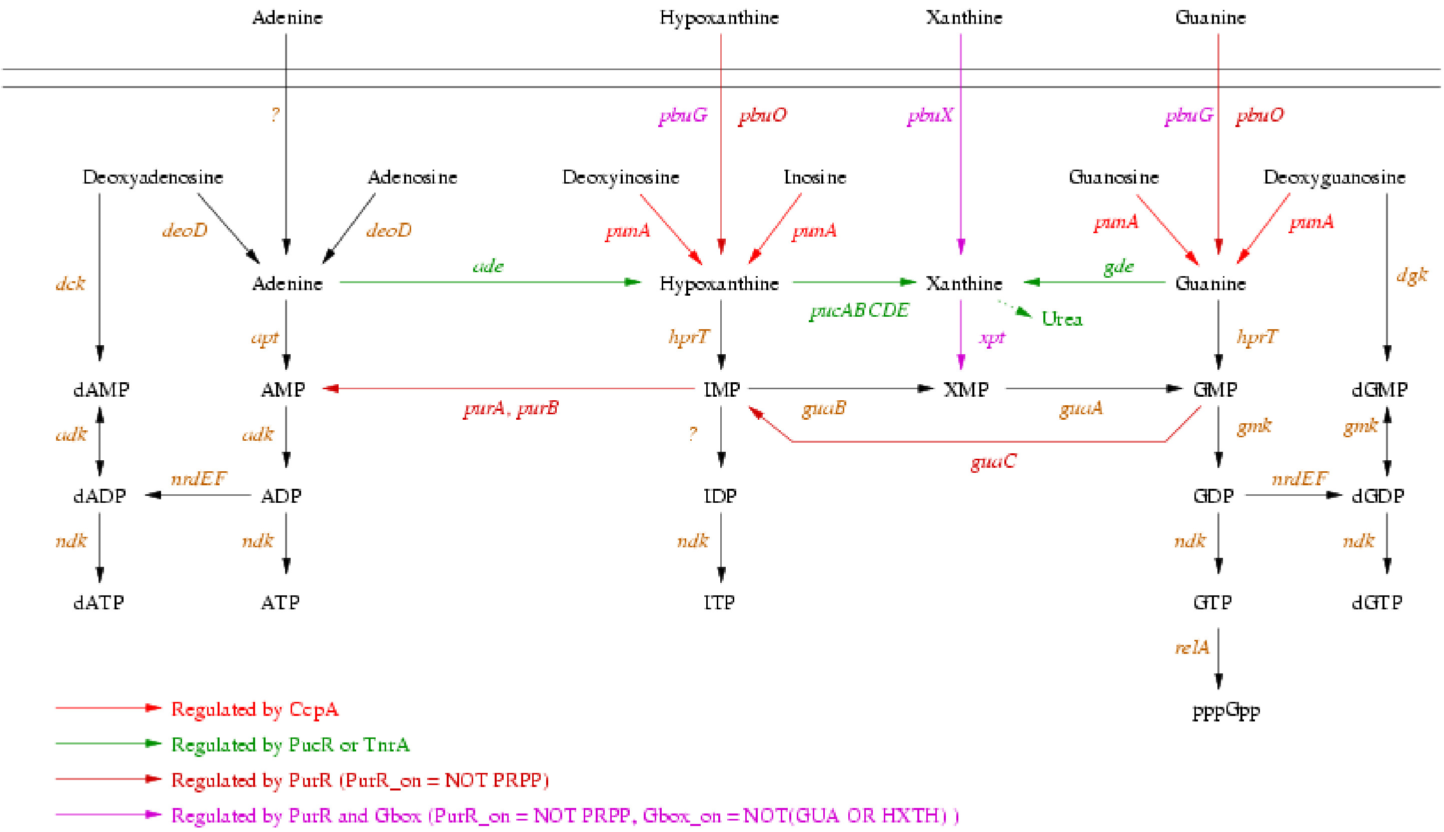

Figure 47: Purines salvage pathway

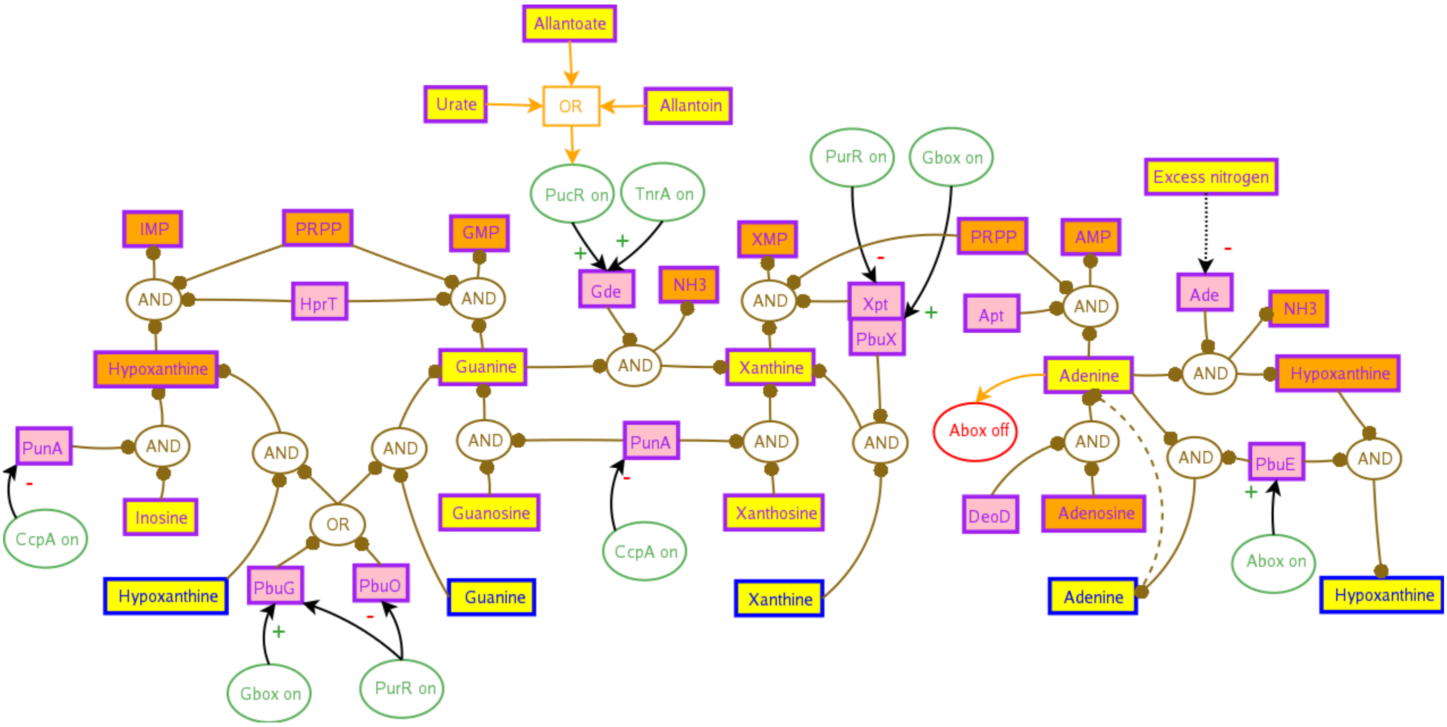

Figure 48: Regulation of purines salvage pathway

Purines catabolism

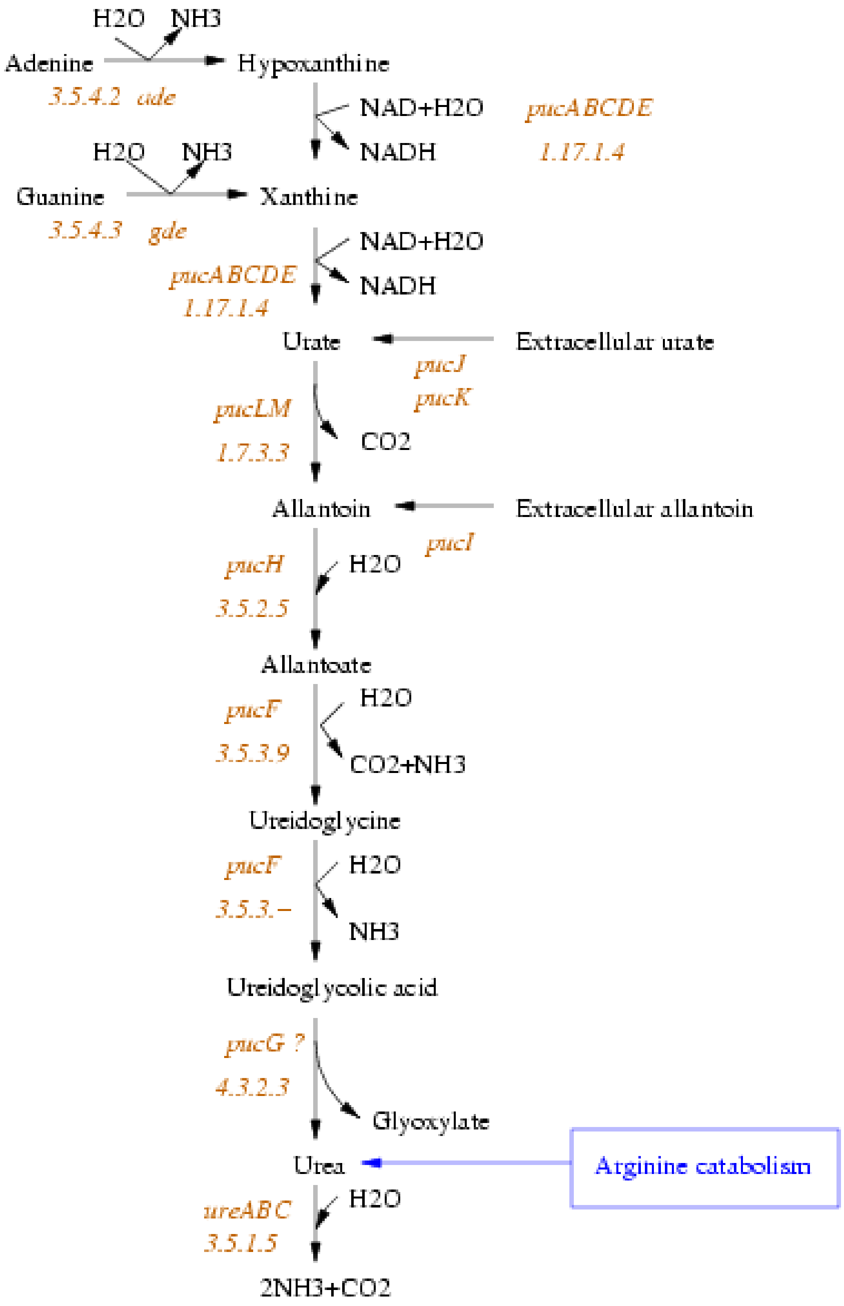

Figure 49: Purines catabolism

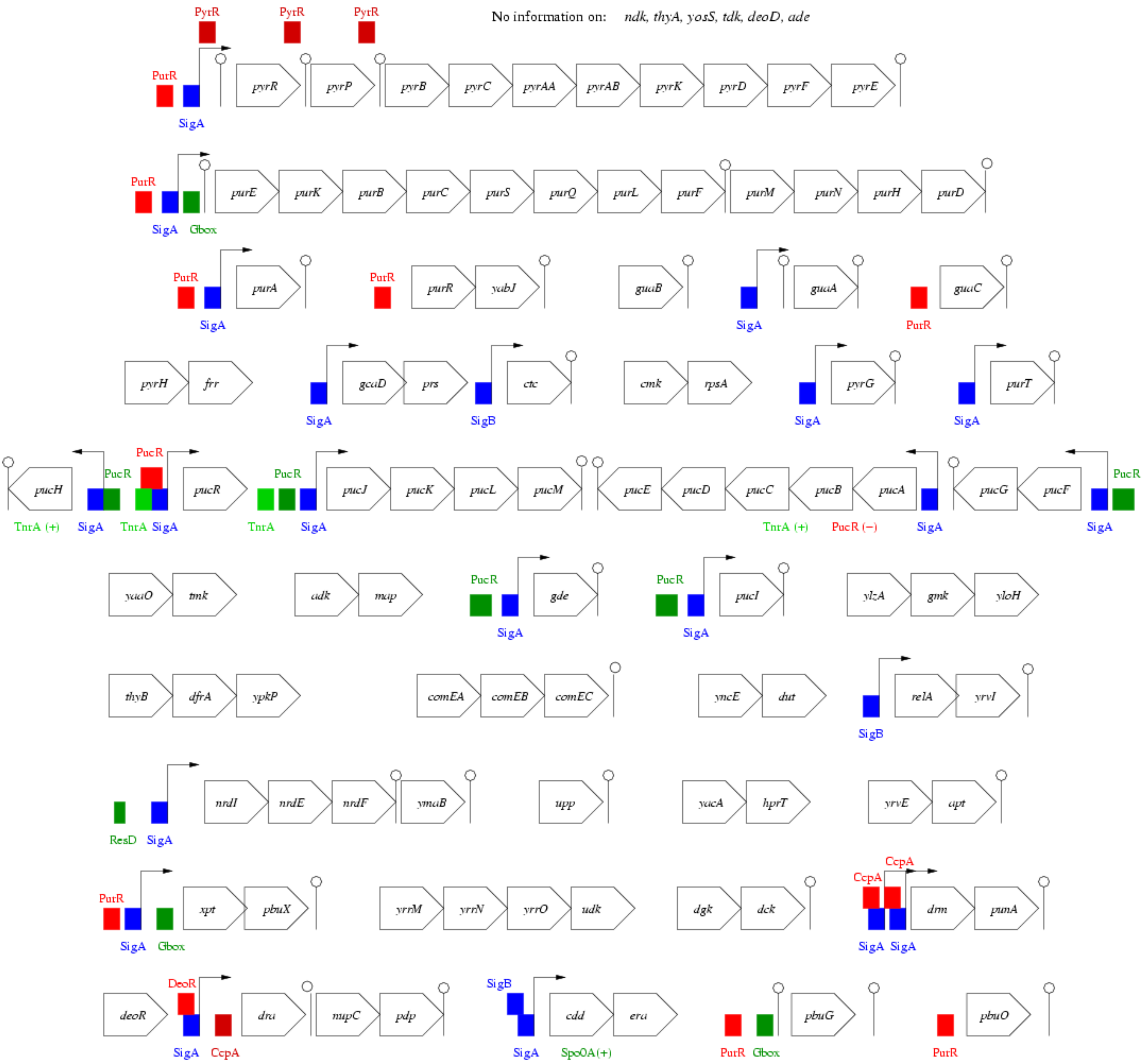

Figure 50: Operons involved in nucleotides metabolism

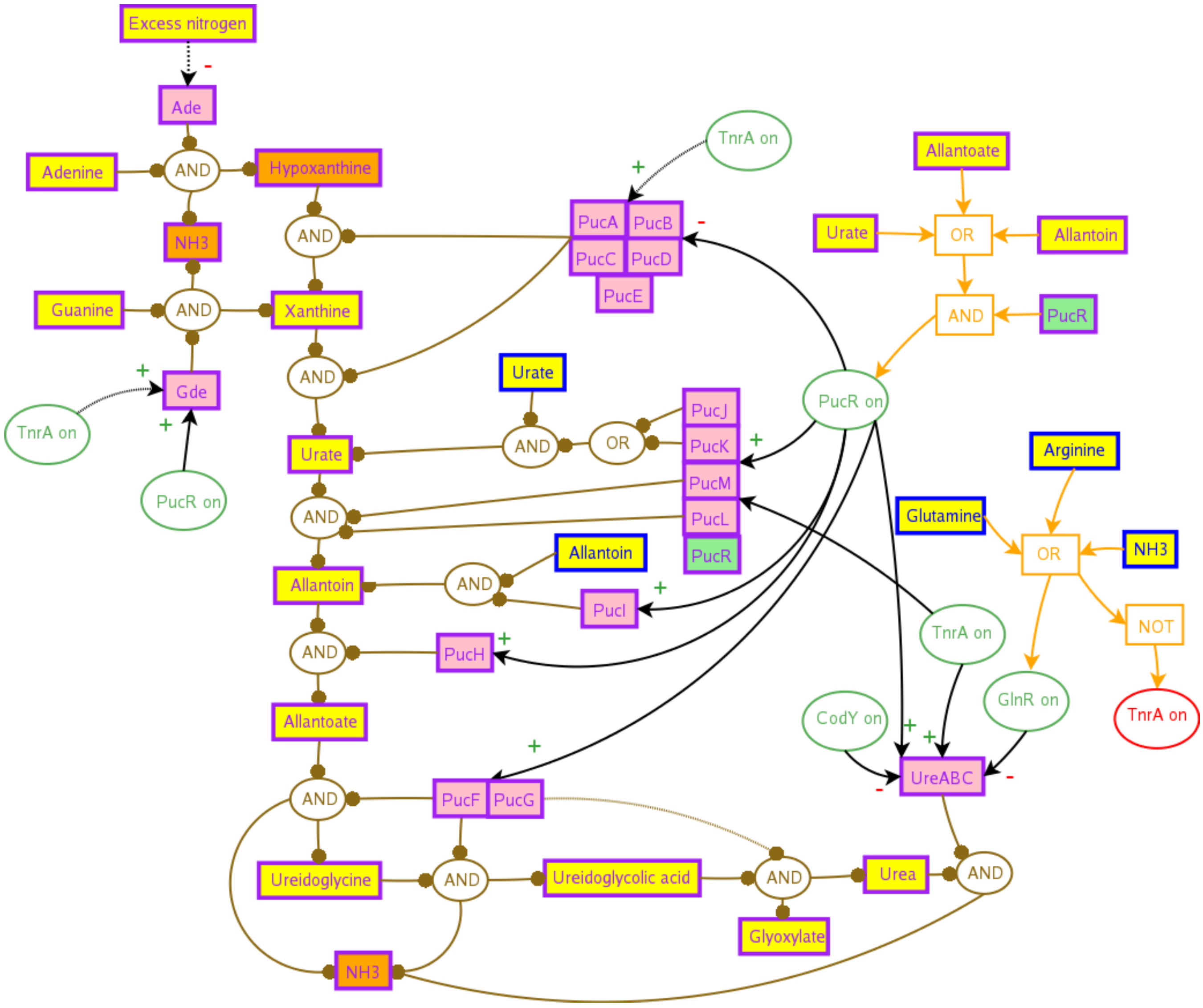

Figure 51: Regulation of purines catabolism

Pyrimidines salvage pathway

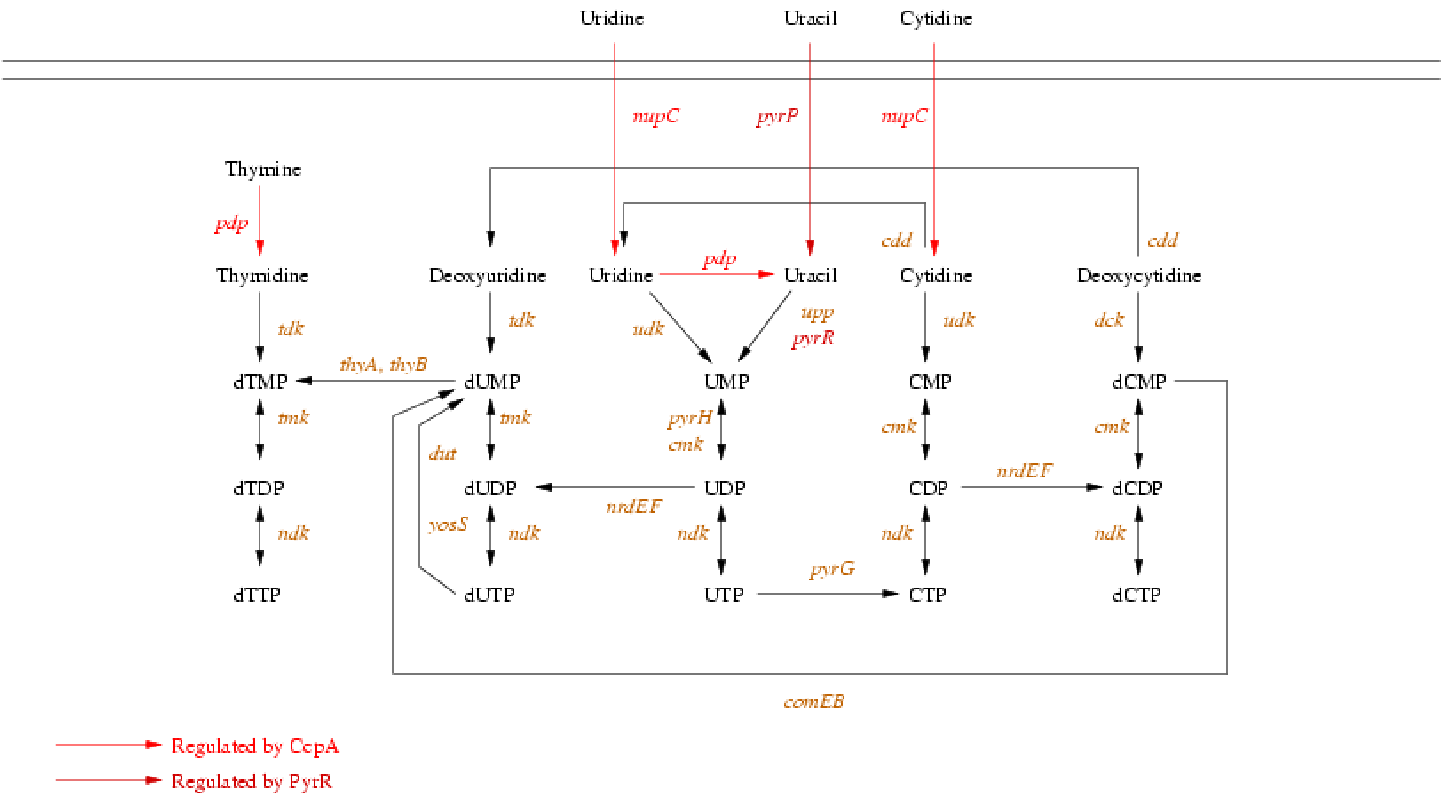

Figure 52: Pyrimidines salvage pathway

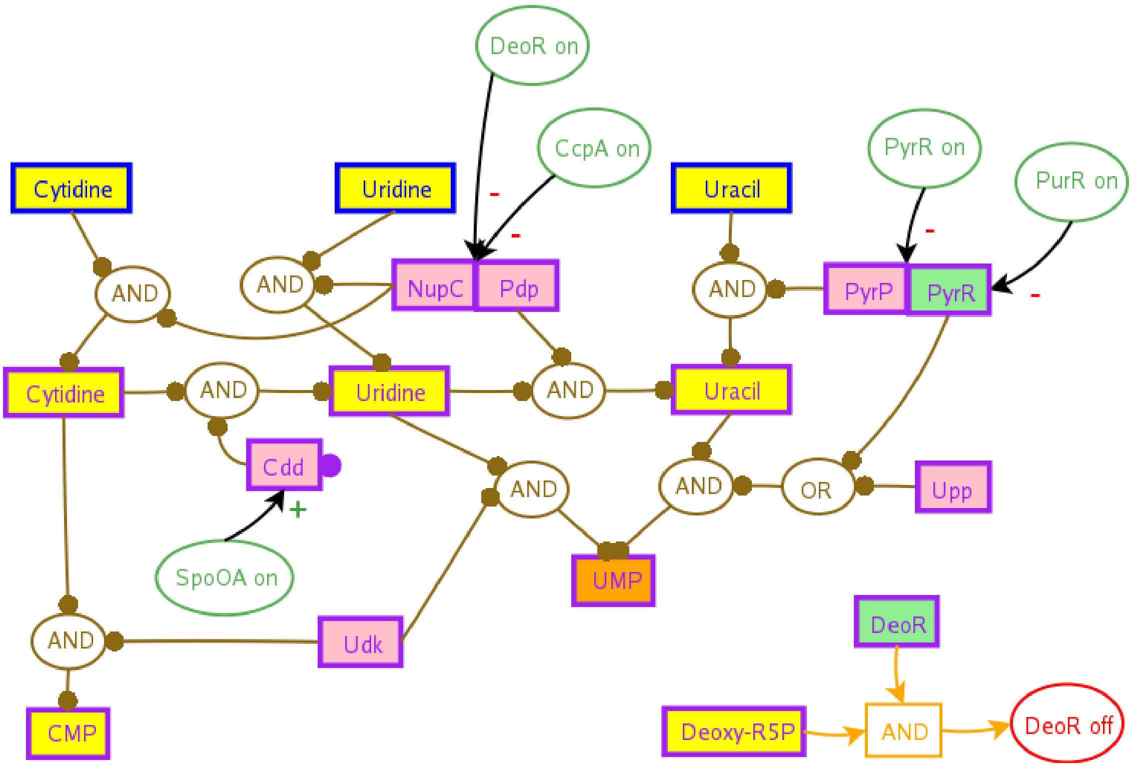

Figure 53: Regulation of pyrimidines salvage pathway

5. Fatty-acids metabolism

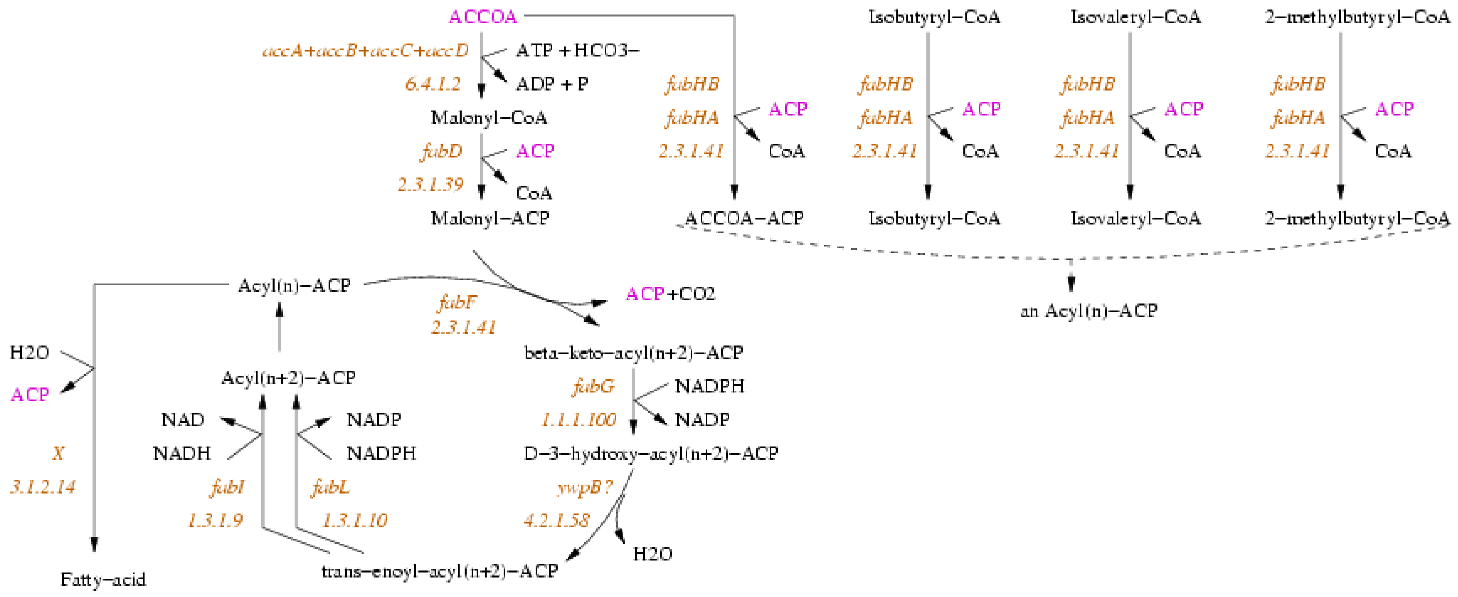

Figure 54: Fatty-acids synthesis

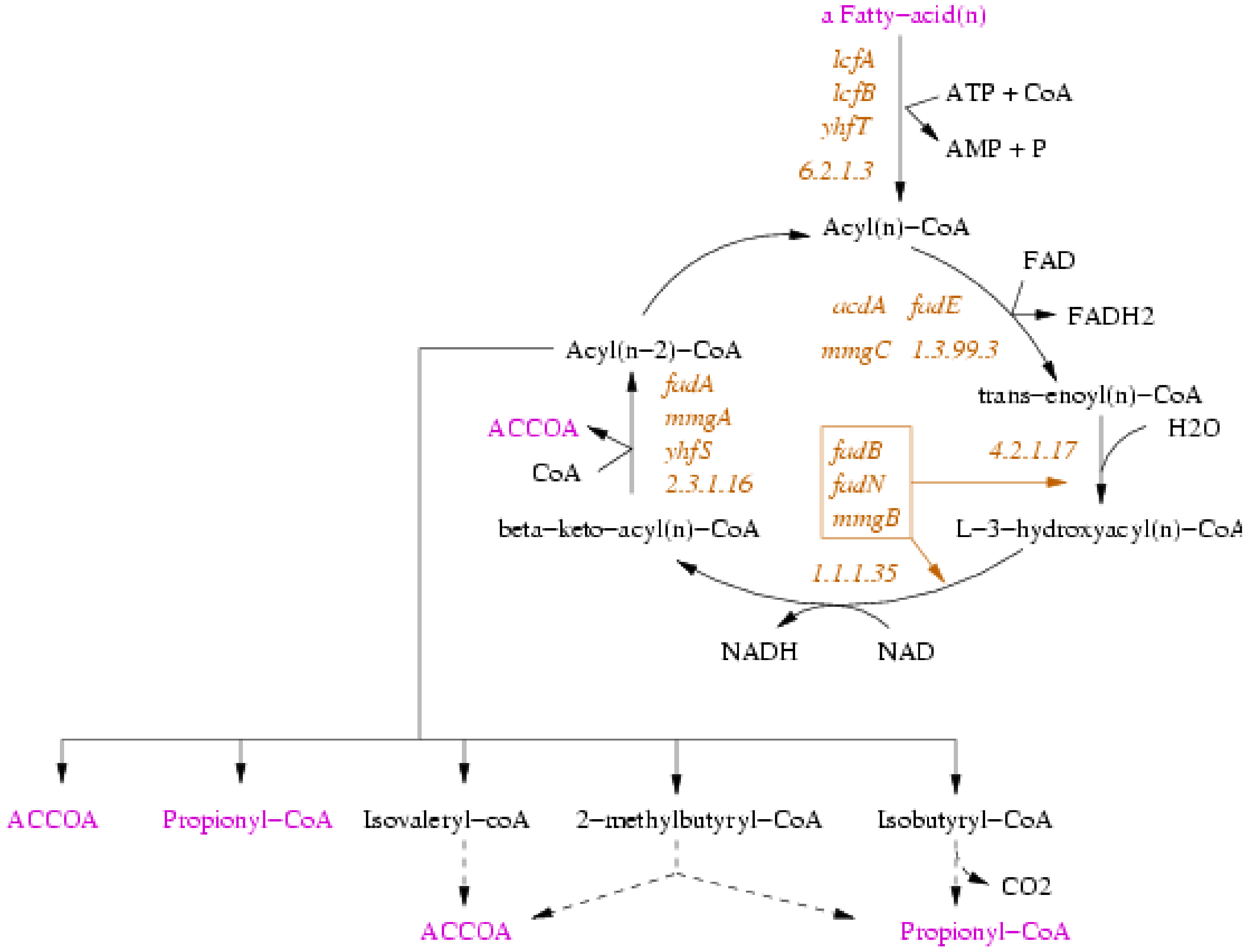

Figure 55: Fatty-acids degradation

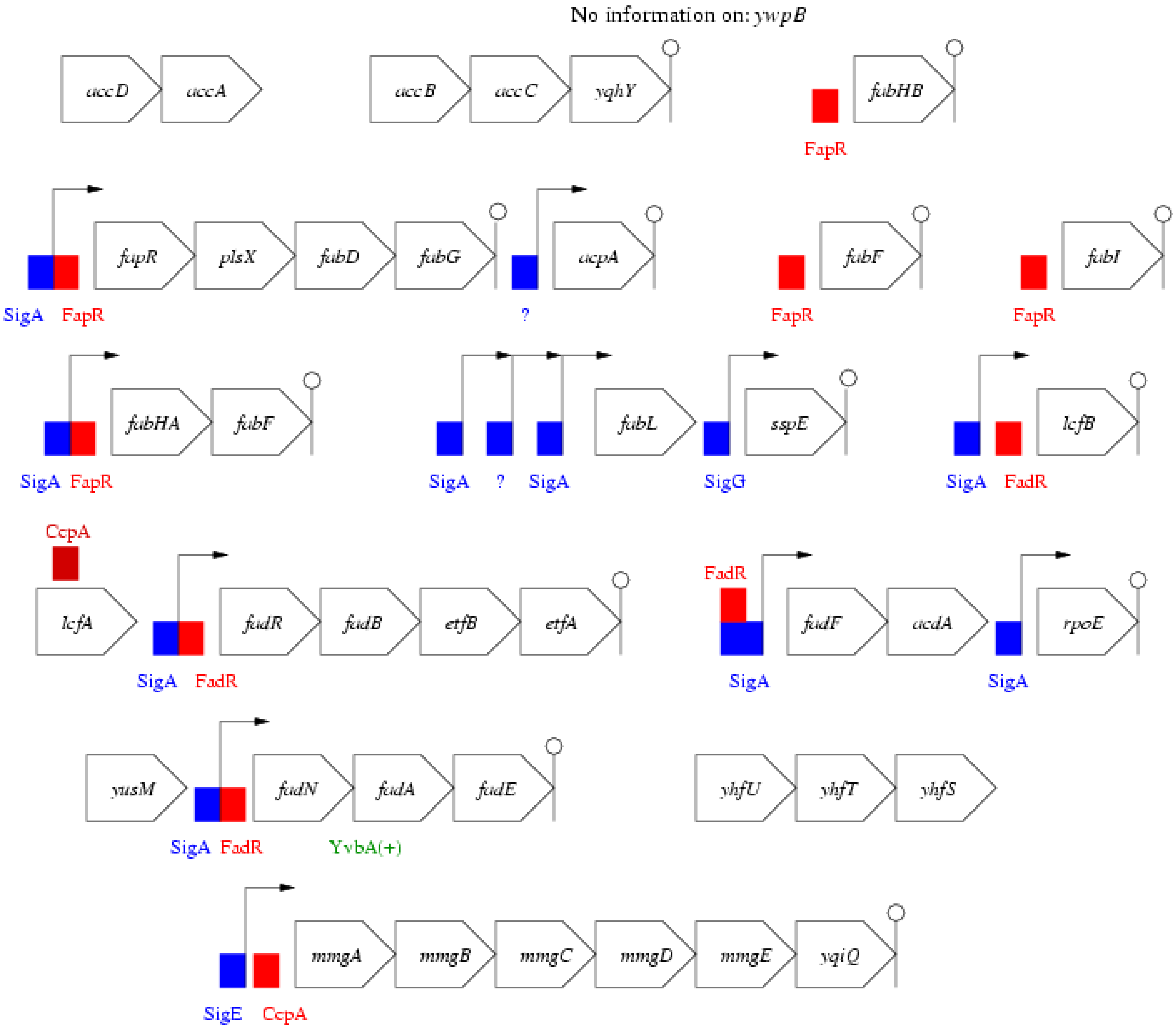

Figure 56: Operons involved in fatty-acids metabolism

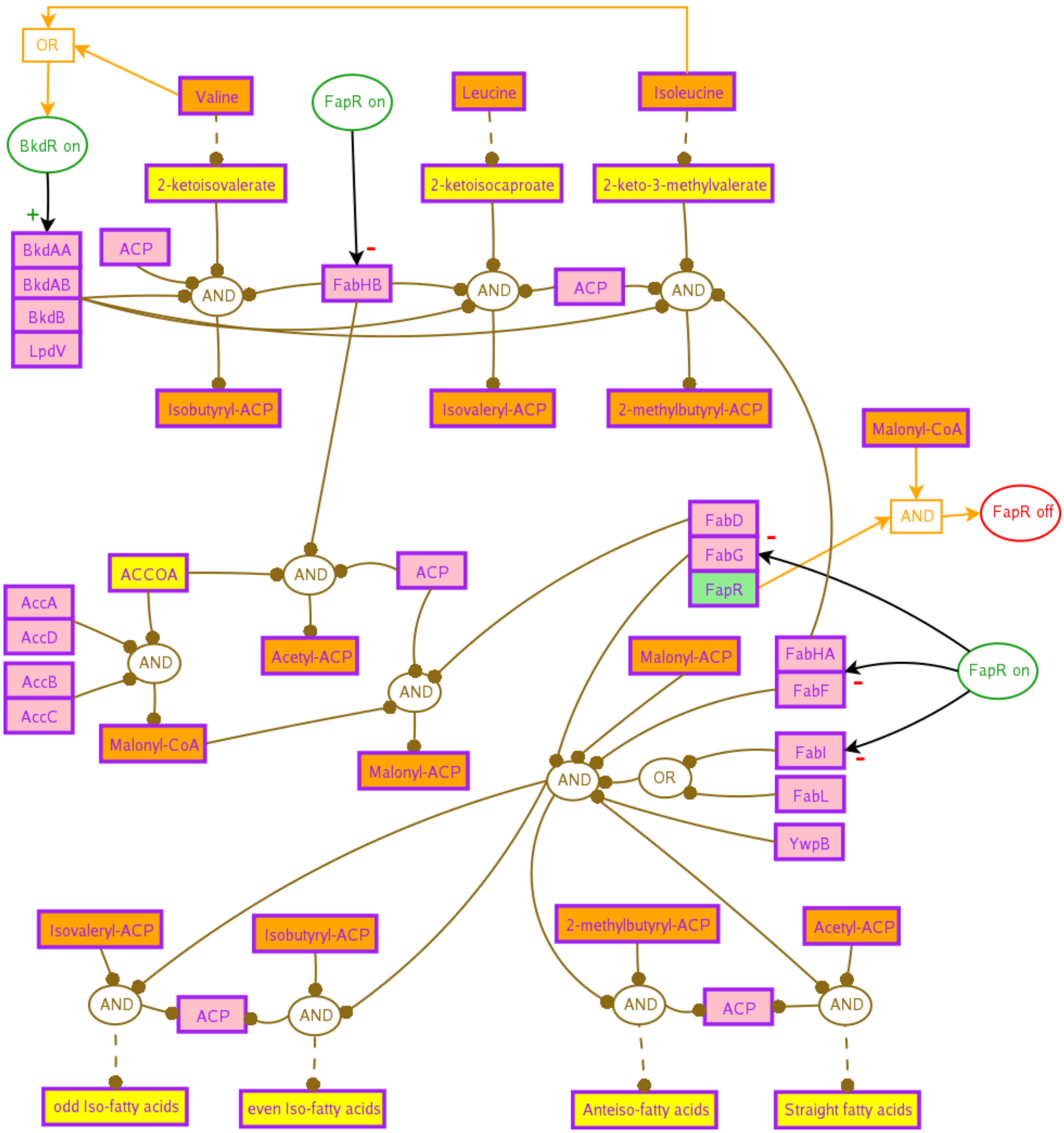

Figure 57: Regulation of fatty-acids synthesis

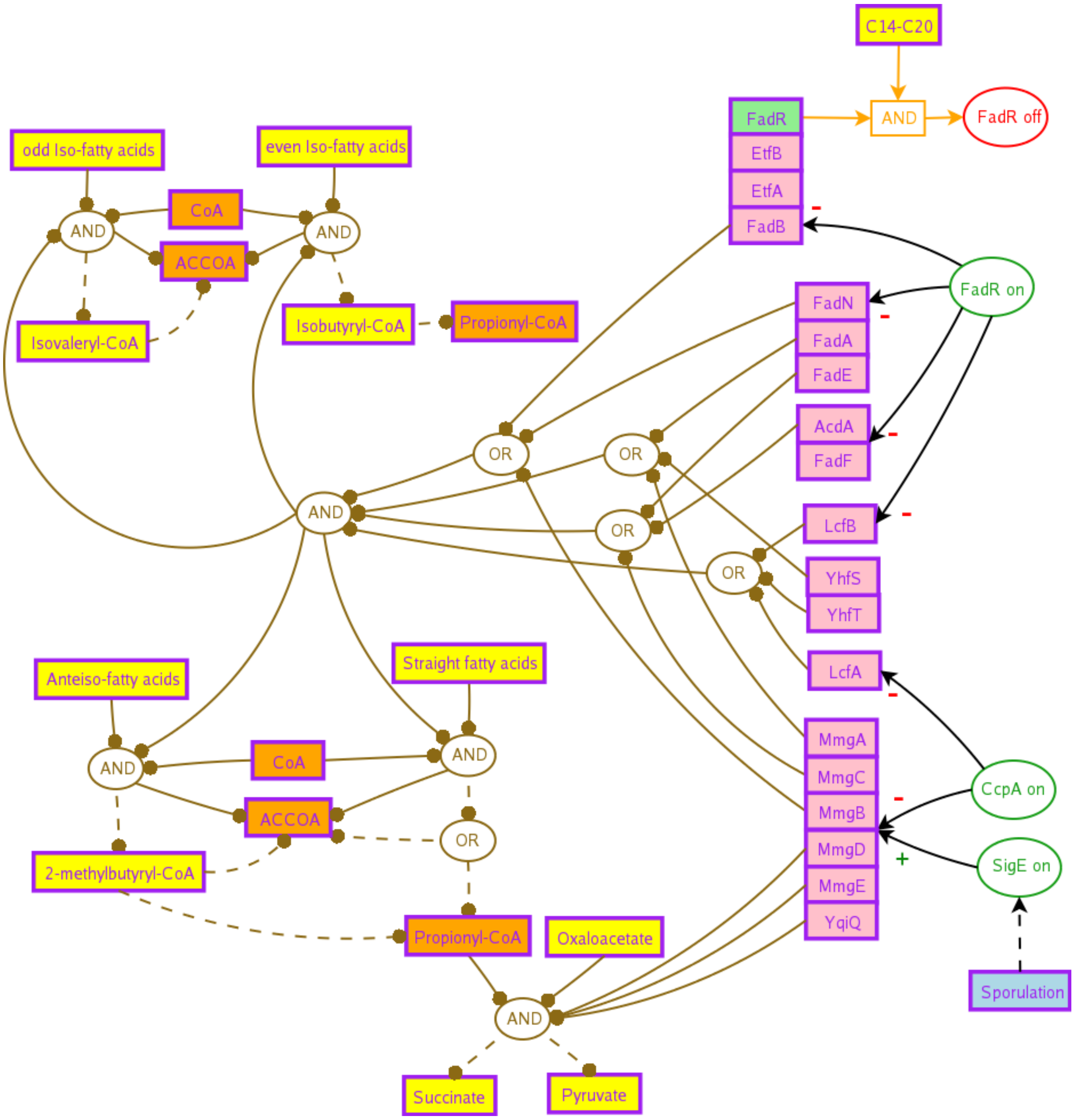

Figure 58: Regulation of fatty-acids degradation
