# Supplementary material for: Reconstruction and analysis of the genetic and metabolic regulatory networks of the central metabolism of Bacillus subtilis
Source: BMC Syst Biol. 2008 Feb 26;2:20. doi: 10.1186/1752-0509-2-20 (PMC2311275; doi:10.1186/1752-0509-2-20)
Supplement: Additional File 7 — Mathematical definition of module. The file presents the study of the steady-state behaviour of the main control structures identified in metabolic pathways. [file 1752-0509-2-20-S7.pdf]

Some mathematical results related to the definition of local and global regulation

In this Additional file, we present the method and the material used for identifying the local and global regulations. The identification method is based on the sensitivity analysis. It is then related to classical tools used in the nonlinear analysis of controlled systems [1] and also to a classical techniques in the field of metabolic analysis : the MCA approach. The MCA analysis reveals that the equilibrium analysis of the metabolic pathways and the investigation of their sensitivities with respect to various parameters like flux or enzyme concentration variations pave the way to a better understanding of how a metabolic system works (see e.g. [5, 4, 6]).

The classical MCA analysis focus on a model of the enzymatic dynamics. In contrast with, our approach is based on a model of both the enzymatic dynamics and of the genetic dynamics.

Nevertheless, even if the enzymatic and genetic controllers of the different pathways are based on closely related structures, their detailed structure are actually different. The analysis is then necessary qualitative due to the important number of pathways.

We thus choose to illustrate the local regulation notion on one of the most common and well-known structure of metabolic pathway regulation: a linear metabolic pathway where both the genetic and the enzyme activity control involve the end product of the pathway. This control structure appears in the biosynthesis pathways of various amino acids like tryptophan, or branched-chain amino acids in *B. subtilis.* Thanks to the analysis of the control structure properties, we are able to explain how such sub-systems could be represented by elementary functional modules.

Finally, we illustrate that the important properties of the linear pathway controlled by the end product are also obtained in other pathways with different control structures.

Linear pathway control by the end product as an example of local regulation

We consider a linear metabolite reaction with many enzymatic reactions. So let us consider many metabolites in a linear cascade such that :

$$\nu_1 \longrightarrow X_1 \xrightarrow{\mathbb{E}_1} X_2 \xrightarrow{\mathbb{E}_2} \cdots \xrightarrow{\mathbb{E}_{i-1}} X_i \xrightarrow{\mathbb{E}_i} \cdots \xrightarrow{\mathbb{E}_{n-1}} X_n \xrightarrow{\mathbb{E}_n} \nu_n$$

where each X_i is a metabolite and $\mathbb{E}_i$ corresponds to an enzyme. We assume that pool X_1 of the first metabolite has an input flux ν_1 and pool X_n of the last one has an output flux ν_n.

We then associate to each enzyme, $\mathbb{E}_i$, its reaction rate, f_i which leads to

[1] As for example in the gain-scheduling framework see [2].

describe the linear pathway through this classical model:

$$\begin{cases} \dot{x}_1(t) = \nu_1(t) - E_1(t)f_1(x_1(t), x_n(t)) \\ \dot{x}_2(t) = E_1(t)f_1(x_1(t), x_n(t)) - E_2(t)f_2(x_2(t)) \\ \vdots \\ \dot{x}_n(t) = E_{n-1}(t)f_{n-1}(x_{n-1}(t)) - E_n(t)f_n(x_n(t)) \end{cases} \tag{1}$$

where x_i is for each $i \in \{1, \cdots, n\}$, the concentration of X_i and where E_i corresponds to the concentration of $\mathbb{E}_i$ for each $i \in \{1, \cdots, n\})$. Finally ν_1 and ν_n are two fluxes.
We then assume that for each $i \in \{2, \cdots, n\}$, $f_i(x) \geq 0$ for any $x \geq 0$ and $f_i(0) = 0$ and that $f_i(x_i)$ is an increasing function of x_i. Moreover, $f_1(x_1, x_n) \geq 0$ for any $x_1 \geq 0$ and any $x_n \geq 0$ and $f_1(0, x_n) = 0$ for any $x_n \geq 0$. Finally, for any given $x_1 \geq 0$ and any $x_n \geq 0$, $f_1(x_1, x_n)$ is nonincreasing function of x_n.

We then now assume that all the enzymes of the metabolic pathway belong to the same operon. That means the required genes are organized in a series of genes transcribed in a single mRNA. Thus, we can assume in this case that the enzymes of these pathways are such that their amounts are equal [7] which leads to assume that

$$E_1(t) = E_2(t) = \cdots E_{n-1}(t) \stackrel{\Delta}{=} E(t)$$

and by definition, $E_n(t)$ is an unrelated quantity. Typically, in case of amino-acids, $\mathbb{E}_n$ often corresponds to a tRNA synthase and its concentration is setting by an unrelated mechanism. For this specific structure, ν_n is thus defined by

$$\nu_n(t) = E_n(t)f(x_n(t))$$

and corresponds to the flux of the end product provided to the next module.

It remains to describe the genetic control by the end product. To this purpose, we then assume that the instantaneous production of enzymes concentration is described by $g(x_n(t))$, a nonincreasing function of x_n. Furthermore, g is assumed to be positive and bounded, *i.e* there exits $M > 0$ such that for any $x_n \geq 0$, we have $g(x_n) \leq M$ and $g(x_n) \geq 0$ for any $x_n \geq 0$. We finally assume that there exits $x^b > 0$ such that $g(x^b) = 0$.

Stationary phase

In this section, we consider that the bacteria population is in stationary phase. Then, from above assumptions on the enzyme production, the dynamic of enzyme production is given by the following differential equation[2]:

$$\dot{E}(t) = g(x_n(t)). \tag{2}$$

So, the model associated to the feedback-loop between the metabolic path-

[2] In order to keep the explanation simple, we assume here that the degradation of enzymes could be neglected.

way and the genetic control is then given by:

$$\begin{cases} \dot{x}_1(t) = \nu_1 - E(t) f_1(x_1(t), x_n(t)) \\ \dot{x}_2(t) = E(t) f_1(x_1(t), x_n(t)) - E(t) f_2(x_2(t)) \\ \vdots \\ \dot{x}_n(t) = E(t) f_{n-1}(x_{n-1}(t)) - E_n(t) f_n(x_n(t)) \\ \dot{E}(t) = g(x_n(t)). \end{cases} \tag{3}$$

We want to characterize the steady-state regime of the previous dynamical system assuming that pool X_1 has a constant concentration x_1 and that the concentration E_n of enzyme $\mathbb{E}_n$, which allows to manage the flux demand, is also assumed to be constant. Actually, the following proposition can be easily proved:

Proposition 1 *For any given $E_n > 0$ and $x_1 > 0$, system (3) has a unique steady-state regime. Moreover, the equilibrium regime is the unique solution of these equations:*

$$\begin{cases} \bar{E} = \dfrac{E_n f_n(x^b)}{f_1(x_1, \bar{x}^b)} \\ f_i(\bar{x}_i) = f_1(x_1, \bar{x}^b) \ \textit{for}\ i \in \{2, ..., n\} \end{cases} \tag{4}$$

This proposition is a consequence of the fact that $\bar{x}_n = x^b$ in order to ensure that $\dot{E}(t) = g(x_n(t)) = 0$. The other terms are then easily deduced since x_n is now given.

This result allows to deduce that in stationary phase, the end product concentration of the pathway is setting by the genetic control and corresponds to the zero of the function g. We consequently note that the end product concentration is independent of the flux demand $\nu_n = E_n f(x_n)$ and it is also independent of the concentration of pool X_1 (until $x_1 > 0$). By contrast, the concentration of enzymes is a linear function of the flux demand and it has also to change in order to adapt the pathway to a variation of the initial pool X_1. In conclusion, this result implies that the rejection of a constant flux variation (or a constant variation of the concentration of pool X_1) is only made at the genetic level. This control structure is quite well-known in the control field since it corresponds to a nonlinear integral controller ([3, 1]).

Growth phase and the volume variation problem

In this section, we show that during growth phase, the behavior of the control structure is completely different from the one obtained during stationary phase.

So in the sequel, we assume that the growth rate of the population is constant and equal to μ. Then, from the above assumptions on the enzyme production, it is not difficult to show that the enzyme production is now described by the following differential equation:

$$\dot{E}(t) = g(x_n(t)) - \mu E(t). \tag{5}$$

where $\mu E(t)$ could be interpreted as a dilution effect due to the volume variation.

As in the stationary phase, we want to analyze the steady-state regime of the closed-loop system described by this dynamical system:

$$\begin{cases} \dot{x}_1(t) = \nu_1 - E(t)f_1(x_1(t), x_n(t)) \\ \dot{x}_2(t) = E(t)f_1(x_1(t), x_n(t)) - E(t)f_2(x_2(t)) \\ \vdots \\ \dot{x}_n(t) = E(t)f_{n-1}(x_{n-1}(t)) - E_n(t)f_n(x_n(t)) \\ \dot{E}(t) = g(x_n(t)) - \mu E(t) \end{cases} \tag{6}$$

Proposition 2 *For any given $E_n > 0$ and $x_1 > 0$, system (6) has a unique steady-state regime. Moreover, the equilibrium regime is the unique solution of these equations:*

$$\begin{aligned} \bar{E} &= \frac{g(\bar{x}_n)}{\mu}, \\ f_1(x_1, \bar{x}_n)\frac{g(\bar{x}_n)}{\mu} &= E_n f_n(\bar{x}_n), \end{aligned} \tag{7}$$

and $\bar{x}_{i=2,\dots,n-1}$ are such that :

$$\bar{x}_i = f_i^{-1}\left(\frac{E_n f_n(\bar{x}_n)\mu}{g(\bar{x}_n)}\right), \quad i = 2, \dots, n-1$$

Proposition 2 indicates that the steady-state of the end product $\bar{x}_n$ only depends on the function f_1 and g. Then, the steady-state sensitivity to a constant perturbation of the flux demand only depends on the effect of the end product on the activity of the first enzyme and on the control of enzymes production. Hence, the prediction of the metabolic pathway behavior is dramatically simplified even though the pathway is composed by many enzymatic reactions. The pathway can then be now considered (in a steady-state regime) as a simple nonlinear and static relation which links the output concentration of the pathway to the the input concentration and the flux demand.

Even if the properties obtained in the previous proposition are obtained through various (and quite strong) assumptions, we show in the sequel that most of these assumptions could be relaxed and the modular aspect is nevertheless kept. Indeed, in most of the considered cases, the relaxation of assumptions leads to describe the properties of the system by a nonlinear relation which often involves other reaction rate functions of the pathway. But, in all the cases, we always obtained a relation allowing to dramatically simplify the analysis of the system during growth phase.

Remove the operon assumption We assume in this section that the enzymes catalyzing the intermediate reactions of the pathway can be separately regulated. We consider a pathway described by:

$$\begin{cases} \dot{x}_1(t) = \nu_1(t) - E_1(t)f_1(x_1, x_n(t)) \\ \dot{x}_2(t) = E_1(t)f_1(x_1(t), x_n(t)) - E_2(t)f_2(x_2(t)) \\ \vdots \\ \dot{x}_n(t) = E_{n-1}(t)f_{n-1}(x_{n-1}(t)) - E_n(t)f_n(x_n(t)) \\ \dot{E}_1(t) = g(x_n(t)) - \mu E_1(t) \end{cases} \tag{8}$$

where each enzyme $\mathbb{E}_i$ with $i \in \{2, \cdots, n-1\}$ has given and constant concentration. We moreover assume that each rate reaction is bounded and that for any $i \in \{2, \cdots, n-1\}$ there exits K_i, such that

$$\lim_{x_i \to +\infty} f_i(x_i) = K_i. \tag{9}$$

Proposition 3 *For any given $E_n > 0$ and $x_1 > 0$, there exists a unique constant steady-state $(\bar{x}_2, \cdots, \bar{x}_n, \bar{E}_1)$ for (8) such that:*

$$\begin{cases} \bar{E}_1 = \dfrac{g(\bar{x}_n)}{\mu}, \\ f_1(x_1, \bar{x}_n)\dfrac{g(\bar{x}_n)}{\mu} = E_n f_n(\bar{x}_n), \end{cases} \tag{10}$$

if and only if for any $i \in \{2, \cdots, n-1\}$, we have

$$\bar{E}_i > \frac{E_n f(\bar{x}_n)}{K_i}. \tag{11}$$

Furthermore,

$$\bar{x}_i = f_i^{-1}\left(\frac{E_n f_n(\bar{x}_n)}{E_i}\right), \quad i = 2, \ldots, n-1 \tag{12}$$

When there exists $i \in \{2, \cdots, n-1\}$ such that condition (11) is not satisfied then the ith enzyme is saturated. We then recover for $\bar{x}_n$, the result of proposition 2 when the enzyme concentrations of the pathway are compatible with the flux $\nu_n = E_n f(\bar{x}_n)$.

Reversible vs irreversible enzymes Implicitly, in system (1), we have considered that all the enzymes of the pathway are irreversible: the flux through each enzyme only depends on its substrate and not on its product. We then investigate hereafter the consequence of this strong assumption on the steady-state regime of the system. If we assume that that all the enzymes of the pathway are reversible and belong to the same operon, then the system is given by:

$$\begin{cases} \dot{x}_1(t) = \nu_1(t) - E(t) f_1(x_1(t), x_2(t), x_n(t)) \\ \dot{x}_2(t) = E(t) f_1(x_1(t), x_2(t), x_n(t)) - E(t) f_2(x_2(t), x_3(t)) \\ \vdots \\ \dot{x}_n(t) = E(t) f_{n-1}(x_{n-1}(t), x_n(t)) - E_n(t) f_n(x_n(t)) \\ \dot{E}(t) = g(x_n(t)) - \mu E(t) \end{cases} \tag{13}$$

where the functions $f_i(x_i, x_{i+1})$ for $i = 2...n-1$ are increasing in x_i and decreasing in x_{i+1} and the function $f_1(x_1, x_2, x_n)$ is increasing in x_1 and decreasing in x_2 and x_n. We have to consider two main cases:

1. The first enzyme is assumed irreversible.
In this case, it is easy to show that $\bar{x}_n$ has the same behavior as in the irreversible case. Indeed, if the function f_1 does not depend on the metabolite X_2 then $\bar{x}_n$ satisfies (7).

2. The first reaction is reversible.
In this case, $\bar{x}_n$ depends of the other reaction rates. Indeed, at the steady-state, we have

$$g(\bar{x}_n) f_1(x_1, \bar{x}_2, \bar{x}_n) = \bar{E}_n f_n(\bar{x}_n). \tag{14}$$

Then in order to compute $\bar{x}_n$, we have to compute $\bar{x}_2$. To this purpose, we note that we have:

$$g(\bar{x}_n)f_{n-1}(\bar{x}_{n-1}, \bar{x}_n) = \bar{E}_n f_n(\bar{x}_n) \tag{15}$$

and since f_{n-1} is increasing function of x_{n-1} and f_{n-1} and g are two decreasing function of x_n then we deduce that $\bar{x}_{n-1}$ is an increasing function of $\bar{x}_n$. The same occurs for $\bar{x}_{n-2}$ and so on. Finally, we obtain $\bar{x}_2$ as an increasing function $h(\bar{x}_n)$ of $\bar{x}_n$ where h depends on reaction rates $f_2, \ldots, f_{n-1}$. Finally, from (14), $\bar{x}_n$ is thus as follows:

$$g(\bar{x}_n)f_1(x_1, h(\bar{x}_n), \bar{x}_n) = \bar{E}_n f_n(\bar{x}_n). \tag{16}$$

Moreover, if we assume that the ith reaction is irreversible then the steady-state $\bar{x}_2$ only depends on functions $f_2, ..., f_i$ and is completely independent of functions $f_{i+1}, ..., f_{n-1}$. Therefore, the irreversible step is a determinant step for $\bar{x}_n$ since the nature of the enzymatic reactions after the irreversible step does not impact $\bar{x}_n$.

Isoenzyme structure We consider that two isoenzymes E and E' catalyze the first reaction: one is regulated by the end product while the other one is not. In this case, we have the following system:

$$\left\{ \begin{array}{l} \dot{x}_1(t) = \nu_1(t) - E(t)f_1(x_1(t), x_n(t)) - E'(t)f_1'(x_1(t)) \\ \dot{x}_2(t) = E(t)f_1(x_1(t), x_n(t)) + E'(t)f_1'(x_1(t)) - E(t)f_2(x_2(t)) \\ \vdots \\ \dot{x}_n(t) = E(t)f_{n-1}(x_{n-1}(t)) - E_n(t)f_n(x_n(t)) \\ \dot{E}_1(t) = g(x_n(t)) - \mu E_1(t) \end{array} \right. \tag{17}$$

In steady-state, $\bar{x}_n$ satisfies the following equation:

$$f_1(x_1, \bar{x}_n)\frac{g(\bar{x}_n)}{\mu} + E_1^{'} f_1'(x_1) = E_n f_n(\bar{x}_n).$$

Thus the presence of an isoenzyme can be interpreted as a way to increase the flux in the pathway. So the flux can increase either if $\bar{x}_n$ decreases, either if $E_1^{'}$ increases.

Global regulation Now, we consider the same structure as in (6) and we assume that the first enzyme E_1 is also regulated by another factor of another pathway *i.e* we assume that the enzyme concentration E_1 is given by :

$$\dot{E}(t) = g(x_n(t), p(t)) - \mu E(t)$$

where $p(t)$ represents an external factor acting on the genetic control.

Through an adaptation of proposition 2 and assuming that $p(t)$ is constant and equal to $\bar{p}$, the steady state is given by:

$$\left\{ \begin{array}{l} \bar{E} = \dfrac{g(\bar{x}_n, \bar{p})}{\mu}, \\ f_1(x_1, \bar{x}_n)\dfrac{g(\bar{x}_n)}{\mu} = E_n f_n(\bar{x}_n), \end{array} \right. \tag{18}$$

and

$$\bar{x}_i = f_i^{-1}\left(\frac{E_n f_n(\bar{x}_n)\mu}{g(\bar{x}_n, \bar{p})}\right), \quad i \in \{2, \ldots, n-1\}.$$

The factor $\bar{p}$ modifies $\bar{E}$ and thus $\bar{x}_n$ and also all the $\bar{x}_i$.

Other control structures

Other control structures can be found in metabolic regulatory network.

Positive pathways Here, we consider that the enzymes of the pathway are regulated positively by the first metabolite and as in the previous section assume that the genes are structured in operon. Such a system can be described by:

$$\begin{cases} \dot{x}_1(t) = \nu_1(t) - E(t)f_1(x_1(t)) \\ \dot{x}_2(t) = E(t)f_1(x_1(t)) - E(t)f_2(x_2))(t))) \\ \vdots \\ \dot{x}_n(t) = E(t)f_{n-1}(x_{n-1}(t)) - E_n(t)f_n(x_n(t)) \\ \vdots \\ \dot{E}(t) = g(x_1(t)) - \mu E(t) \end{cases} \tag{19}$$

where the enzyme production function $g(x_1)$ is a nondecreasing function of the concentration x_1 of the first metabolite.

Proposition 4 *For $\nu_1 > 0$, system (19) has a unique steady-state regime. Moreover, we have*

$$\bar{E} = \frac{g(\bar{x}_1)}{\mu},$$

and

$$\frac{g(\bar{x}_1)}{\mu} f_1(\bar{x}_1) = \nu_1.$$

Moreover, $\bar{x}_i$ for $i \in \{2, \cdots, n\}$ is increasing function of ν_1.

For such a structure, the input flux ν_1 sets the concentration of all the metabolite pools.

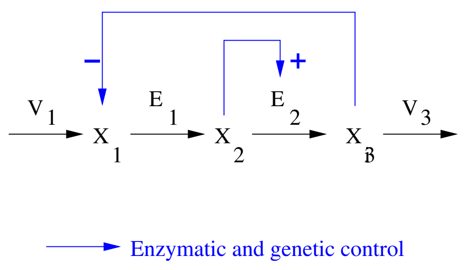

Figure 1: An example of a mixed feedback pathway

Mixed feedback pathways Many interesting situations can be obtained when negative and positive feedbacks are combined for the control of the pathway. Inspired by the biosynthesis pathway of cysteine in *B. subtilis*, we consider a metabolic pathway where the first enzyme is regulated by the end product through both genetic and enzymatic activity controls and one intermediate enzyme is regulated by a positive genetic control (see figure 1). This system is described by:

$$\left\{\begin{array}{l} \dot{x}_1(t) = \nu_1(t) - E_1(t)f_1(x_1(t), x_3(t)), \\ \dot{x}_2(t) = E_1 f_1(x_1(t), x_3(t)) - E_2 f_2(x_2(t)), \\ \dot{x}_3(t) = E_2 f_2(x_2(t)) - E_3 f_3(x_3(t)), \\ \dot{E}_1(t) = g_1(x_3(t)) - \mu E_1(t), \\ \dot{E}_2(t) = g_2(x_2(t)) - \mu E_2(t), \end{array}\right. \tag{20}$$

where g_1 is nonincreasing function of x_3, g_2 is nondecreasing function of its argument. We moreover assume that $\dfrac{\partial f_1}{\partial x_1}(x_1, x_3) > 0\,,\ \dfrac{\partial f_1}{\partial x_3}(x_1, x_3) < 0\,.$ Finally, f_3 is assumed to be nondecreasing function of x_3 and x_1 is constant. From theses conditions, we can prove that for any $E_3 > 0$ and $x_1 > 0$, the steady-state exists and is given by:

$$\left\{\begin{array}{l} \dfrac{g_1(\bar{x}_3)}{\mu} f_1(x_1, \bar{x}_3) = E_3 f_3(\bar{x}_3), \\ \dfrac{g_2(\bar{x}_2) f_2(\bar{x}_2)}{\mu} = E_3 f_3(\bar{x}_3), \\ \bar{E}_1 = \dfrac{g_1(\bar{x}_3)}{\mu}, \quad \bar{E}_2 = \dfrac{g_2(\bar{x}_2)}{\mu}. \end{array}\right. \tag{21}$$

In this case, it can be proved that $\bar{x}_3$ evolves like $\bar{x}_n$ in the control structure of the end product control.

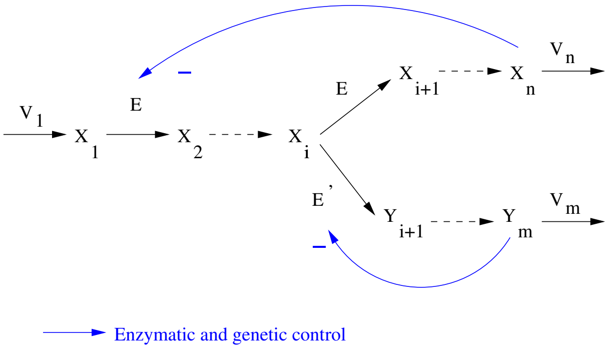

Figure 2: An example of a branched feedback pathway

Branched feedback pathways In this section, we consider a simple case of a branched pathway where the end product X_n is able to inhibit the activity of the first enzyme of the pathway. Moreover, the other branch is only able to inhibit its own synthesis without affecting the other pathway neither at the genetic level nor at the enzymatic activity level. The first enzyme $E_i^{'}$ of the second branch

is regulated by its end product Y_m at the enzymatic and the genetic levels (see figure 2). All these assumptions lead to describe the metabolic system with the following system of differential equations:

$$\begin{cases} \dot{x}_1(t) = \nu_1(t) - E_1(t)f_1(x_1(t), x_n(t)) \\ \dot{x}_2(t) = E(t)f_1(x_1(t), x_n(t)) - E(t)f_2(x_2(t)) \\ \dot{x}_3(t) = E(t)f_2(x_2(t)) - E(t)f_3(x_3(t)) \\ \vdots \\ \dot{x}_i(t) = E(t)f_{i-1}(x_{i-1}(t)) - E(t)f_i(x_i(t)) - E'(t)f'_i(x_i(t), y_m(t)) \\ \dot{x}_{i+1}(t) = E(t)f_i(x_i(t)) - E(t)f_{i+1}(x_{i+1}(t)) \\ \vdots \\ \dot{x}_n(t) = E(t)f_{n-1}(x_{n-1}(t)) - E_n(t)f_n(x_n(t)) \\ \dot{y}_{i+1}(t) = E'(t)f'_i(x_i(t), y_m(t)) - E'(t)f'_{i+1}(y_{i+1}(t)) \\ \vdots \\ \dot{y}_m(t) = E'(t)f_{i+m-1}(y_{i+m-1}(t)) - E'_m(t)f_m(y_m(t)) \\ \dot{E}(t) = g(x_n(t)) - \mu E(t) \\ \dot{E}'(t) = g'(y_m(t)) - \mu E'(t). \end{cases} \quad (22)$$

and the various functions satisfy the same assumption than the one used in section .

We then have

Proposition 5 *For any given $E_n > 0, E'_m > 0$ and any $x_1 > 0$, system (22) has a unique steady-state regime. Moreover,*

$$\bar{E} = \frac{g(\bar{x}_n)}{\mu} \text{ and } \bar{E}' = \frac{g'(\bar{y}_m)}{\mu} \quad (23)$$

where $\bar{x}_n$ and $\bar{y}_m$ are the solution of this system of equation:

$$\begin{cases} \frac{g(\bar{x}_n)}{\mu} f_1(x_1, \bar{x}_n) = E_n f_n(\bar{x}_n) + E'_m f'_m(\bar{y}_m), \\ f'_i\left(f_i{}^{-1}\left(\frac{E_n f_n(\bar{x}_n)}{E}, \bar{y}_m\right)\right) = \frac{E'_m f'_m(\bar{y}_m)}{E'} \end{cases} \quad (24)$$

Due to the monotonicity of the various functions, this proposition leads to a simple characterization of the steady-state behavior of a branched pathway as a relation of the two flux demands and the level of the first metabolite.

References

[1] V. Fromion and G. Scorletti. Performance and robustness analysis of nonlinear closed loop systems using μ_{nl} analysis: Applications to nonlinear PI controllers. In Proc. IEEE Conf. on Decision and Control, Las Vegas, USA, December 2002.

[2] V. Fromion and G. Scorletti. A theoritical framework for gain scheduling. *Int. J. Robust and Nonlinear Control*, 13(6):951–982, 2003.

[3] V. Fromion, G. Scorletti, and G. Ferreres. Nonlinear performance of a PID controlled missile: an explanation. *Int. J. Robust and Nonlinear Control*, 9(8):485–518, 1999.

[4] R. Heinrich and T. A. Rapoport. Mathematical analysis of multienzyme systems. III. Steady-state and transient control. *BioSystems*, 7:130–136, 1975.

[5] H. Kacser and J. A. Burns. The control of flux. *Symposia of the Society for Experimental Biology*, 27:65–104, 1973.

[6] C. Reder. Metabolic control theory; a structural appproach. *Journal of Theoretical Biology*, 135:175–201, 1988.

[7] A. Zaslaver, A. Mayo, M. Ronen, and U. Alon. Optimal gene partition into operons correlates with gene functional order. *Physical biology*, 3:183–189, 2006.
